# Climatic and landscape changes as drivers of environmental feedback that influence rainfall frequency in the United States

**DOI:** 10.1111/gcb.15876

**Published:** 2021-09-23

**Authors:** Rachel A. Moore, Davide Martinetti, E. Keith Bigg, Brent C. Christner, Cindy E. Morris

**Affiliations:** ^1^ School of Earth and Atmospheric Sciences at the Georgia Institute of Technology Atlanta GA 30318; ^2^ BioSP INRAE Avignon France; ^3^ 11 Wesley St. Elanora Heights NSW Australia; ^4^ Department of Microbiology and Cell Science University of Florida Gainesville FL USA; ^5^ Pathologie Végétale INRAE Montfavet France

**Keywords:** climate change, land type, land use, land–atmosphere feedback, rainfall frequency

## Abstract

Previous studies have identified regions where the occurrence of rainfall significantly increases or decreases the probability for subsequent rainfall over periods that range from a few days to several weeks. These observable phenomena are termed “rainfall feedback” (RF). To better understand the land–atmosphere interactions involved in RF, the behavior of RF patterns was analyzed using data from 1849 to 2016 at ~3000 sites in the contiguous United States. We also considered changes in major land‐use types and applied a geographically weighted regression model technique for analyzing the predictors of RF. This approach identified non‐linear and spatially non‐stationary relationships between RF, climate, land use, and land type. RF patterns in certain regions of the United States are predictable by modeling variables associated with climate, season, and land use. The model outputs also demonstrate the extent to which the effect of precipitation, temperature, and land use on RF depend on season and location. Specifically, major changes were observed for land use associated with agriculture in the western United States, which had negative and positive influences on RF in summer and winter, respectively. In contrast, developed land in the eastern United States correlated with positive RF values in summer but with negative ones in winter. We discuss how changes in climate and land use would be expected to affect land–atmosphere interactions, as well as the possible role that physical mechanisms and rain‐enhanced bioaerosol emissions may play in the spatiotemporal changes observed for historical patterns of rainfall frequency in the United States.

## Graphical abstract

This study analyzed the influence of rainfall on subsequent rainfall (i.e., rainfall feedback) from 1960 to 2010 at ~3000 sites in the contiguous United States using a geographically weighted regression model. The model outputs demonstrate the extent to which the effect of precipitation, temperature, and land use on rainfall feedback depend on season and location.

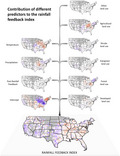

## 1 INTRODUCTION

By the late 15th century, North America was inhabited by ~7 million humans, and their activities were responsible for substantially modifying native land cover (Denevan, 1992). The ensuing period of European colonization and population expansion resulted in the conversion of large expanses of forests, grassland, and shrubland into roads, settlements, and agricultural ecosystems (Goldewijk et al., 2011). By 1850, the human population in North America was ~23 million, with westward expansion in the United States after 1860 increasing the number of farms to six million over a span of 20 years (Shannon, 1989). According to the 2010 census, the ~2 million farms in the United States comprise the largest category of land use, representing 44% of the total (USDA). Moreover, human activities that modify landscapes in North America have accelerated during the last century (Yu & Lu, 2018), have been linked to significant changes in regional climate (Pitman et al., 2011), and have increased the likelihood of extreme weather events (Findell et al., 2017; Lejeune et al., 2018; Teuling et al., 2010).

Land‐cover changes can directly or indirectly affect climate by influencing surface properties such as albedo, roughness, soil moisture, and the efficiency of evapotranspiration, as well as the partitioning of latent and sensible heat, wind speed, and atmospheric turbulence (de Noblet‐Ducoudré et al., 2012; Nair et al., 2011; Zhang et al., 2015). While the specific landscape changes and tipping points necessary for altering such land–atmosphere interactions have not been thoroughly constrained, the climatic effect of changing land cover is well documented. For example, measurable changes in precipitation and climate are observed in recently urbanized and deforested regions (Chambers & Artaxo, 2017; Khanna et al., 2017; Pathirana et al., 2014; Seino et al., 2018).

Alteration of the Bowen ratio (i.e., ratio of sensible to latent heat), soil moisture, and surface albedo are all effects that have been shown to be associated with feedback on rainfall that have durations of several days (Eltahir, 1998; Tuttle & Salvucci, 2016). For example, decreased surface albedo can increase absorption of incident radiation and surface temperature, which in turn leads to more evaporation from the surface. Vegetated land cover also increases evapotranspiration, thereby increasing atmospheric moisture (Gerken et al., 2018) and enhancing the probability of afternoon precipitation (Findell et al., 2011). Afforestation‐enhanced surface roughness may also be associated with enhanced evapotranspiration and precipitation (Yosef et al., 2018). Conversely, changing a heavily vegetated forest to agricultural land use has been shown to reduce evapotranspiration, cloudiness, and precipitation (Werth & Avissar, 2002). Wind speed also effects evapotranspiration and can be altered by changes in land use (Li et al., 2017).

In addition to the direct physical effects of vegetation and soil on air masses, land‐use changes also alters the sources and emission (Morris et al., 2014; Suski et al., 2018) of aerosols, including bioaerosols that consist of microorganisms, biological propagules, and organic detritus (Wery et al., 2018). Aerosols have important direct and indirect effects on climate, and the indirect roles of bioaerosols emitted from terrestrial environments may have a role in the feedback between the land and atmospheric system (Morris et al., 2014). For example, the properties of certain bioaerosols allow them to affect meteorological processes by acting as cloud condensation or ice nuclei, with the potential to influence cloud formation, cloud lifetime, cloud structure, precipitation formation, and radiative fluxes (Andreae & Rosenfeld, 2008; Bigg et al., 2015; Morris et al., 2004).

Time series analyses have revealed an association between rainfall and the frequency of rain events that subsequently occur 20 days after a rain event (Morris et al., 2017). A statistical indicator for the measurable influence of rainfall on subsequent rainfall, termed the rainfall feedback (RF) index, was quantified and mapped at 2940 sites across the continental USA using available data sets. The distribution and range of positive and negative RF index values across the United States appear to be associated with specific geographical and climate regions, as well as landscape types (Morris et al., 2017). Due to the cloud‐active properties of certain bioaerosols, Morris et al. (2016) considered various phenomena (e.g., changes in soil moisture, Bowen ratio, evapotranspiration, and surface albedo) that could explain how landscape processes could affect meteorological conditions several weeks after a precipitation event. They concluded that changes in the emission fluxes of cloud‐active bioaerosols was the most plausible explanation for variation in the RF index among different land‐cover and land‐use types. However, studies have also shown that soil moisture “memory” can extend land–atmosphere feedback that effect precipitation for periods of several weeks (Dirmeyer et al., 2009; McColl et al., 2019), suggesting that physical mechanisms could provide a plausible explanation for the RF patterns observed.

Given that vegetation exerts a multifaceted influence on hydrologic cycling, we analyzed RF variability to test the hypothesis that land use and type are predictors of the amplitude and type of feedback (i.e., a negative or positive RF index). Assuming that the effects may be nonlinear and vary spatially (i.e. spatially nonstationary), we used season, climate, land use, and topography as variables for geographically weighted regression (GWR) models that predict the RF index. The model outputs were used to assess the effect of changes in climate and land‐use proportions in the past and indicate that rainfall patterns in regions of the United States correlate well with human‐induced changes in landscape from 1849 to 1960 ce. To contribute to pertinent experimental design for data acquisition to evaluate hypotheses about underlying mechanisms, we examine the range of land–atmosphere interactions that can explain these trends and discuss the implications of continued land‐use changes for altering rainfall patterns in the United States.

## 2 MATERIALS AND METHODS

### 2.1 Model construction

To explore spatial nonstationarity and varying relationships between RF and decisive factors, a multivariate GWR model was used. The GWR is an extension of ordinary least square (OLS) regression analysis (Fotheringham et al., 2002). An advantage of this method is that GWR models are sensitive to nonstationary interactions between the dependent and independent variables, whereas linear models are limited in their capacity to resolve the spatial autocorrelation of the regression residuals (see Table S1).

The GWR model estimates a local coefficient for each location and explanatory variable and is defined by the equation:

(1) $$Y_{i}=\beta_{i0}+\sum_{k=1}^{m}\beta_{\mathrm{ik}}x_{\mathrm{ik}}+\varepsilon_{i},i\;\in \left\{1,\ldots ,n\right\}$$

where *Y_i_* is the dependent variable at location *i*, *x_ik_* is the *k*th independent variable at location *i*, *m* is the number of independent variables, *β_i_*_0_ is the intercept parameter at location *i*, *β_ik_* is the local regression coefficient for the *k*th independent variable at location *i*, and *ε_i_* is the random error at location *i*. The local coefficients were obtained via an OLS estimation by incorporating a sample of observations weighted according to their proximity to location *i*, which was based on a distance‐decay Gaussian weight function. The number of observations used to estimate each local coefficient was determined by an adaptive bandwidth that was based on minimizing the drop‐1 cross‐validation criteria (Bivand et al., 2017; Fotheringham et al., 2002). Thus, the relationship between the independent variables and RF index is linear locally (i.e., within the estimated bandwidth). At the global scale, the effect of a single predictor does not contribute linearly to the dependent variables.

### 2.2 Dependent variable and study area

RF indices were calculated previously (Morris et al., 2017) and are based on analysis of an average of 103 years of rainfall measurement (range of 17 to 163 year per site) from 2940 meteorological stations within the contiguous United States of America (available at the Rainfall Feedback Maps database; w3.avignon.inra.fr/rainfallfeedback/index.html ). This metric is based on the number of rain events 20 days after a “key day,” which is defined as an above average rain event (range of 0.13 to 74 mm of rainfall among the sites; Bigg et al., 2015; Morris et al., 2017). For each site, the RF index was calculated by a cumulative sum of differences (CD) across every key day for which data are available using the following equation (Bigg et al., 2015; Morris et al., 2016):

(2) $${\text{CD}}_{j}=\sum_{j^{\prime }=1}^{j}\left\{\left(\frac{1}{n}\sum_{k\;\in \;\text{key}\;\text{days}}d_{k+j^{\prime }}\right)‐\left(\frac{1}{n}\sum_{k\;\in \;\text{key}\;\text{days}}d_{k‐j^{\prime }}\right)\right\}$$

An individual rainfall event is represented by *d_k_* on key day *k*, *d_k_*_+_ *_j_*_′_ is a rainfall at *j*′ days after the key day, *d_k_*_‐_ *_j_*_′_ is rainfall *j*′ days before the key day, and *n* is the number of key days in the total collection of rainfall data for that site. A negative or positive value for the RF index indicates a decrease or increase, respectively, in the number of days before subsequent rainfall when compared to the time preceding a key day rainfall.

### 2.3 Independent variables

A group of 73 candidate variables were selected to identify those most important in RF prediction (data sources in Table S2). Raster data for the candidate variables were cropped to 10‐km diameter buffers that intersected with watersheds around each meteorological station. Continuous data (e.g., climate variables) were averaged within the buffer, whereas discrete variables (i.e., land use and landscape) were extracted for each buffer and transformed into area proportions (value between 0 and 1). Land‐use variables were parameterized by determining the proportion of US land (in 1970 ce and the difference between 1970 and 2011 ce) that classified into seven broad categories: developed land, barren land, forest (mixed, deciduous, and woody wetland), evergreen forest, shrubland, agriculture and herbaceous, and wetland. Climate variables included precipitation (seasonal average; minimum and maximum rainfall per month) and temperature data in 1950 (seasonal average: minimum and maximum temperature per month), and the change in precipitation and temperature data from 1950 and 2000 (Table S2). The dates of 1950 and 2000 were chosen based on raster data resolution and availability (Tables S2 and S3).

### 2.4 Model specification

To differentiate the effects of short‐term (e.g., season, canopy density, and the presence of cultivated crops) from long‐term trends attributable to land use and land‐use change, data collected from spring to summer and fall to winter were modeled separately. For simplicity, the seasonally parsed data are hereafter referred to as summer (April to September) and winter (October to March). The models derived for summer and winter are identical in their relationship between the dependent and independent variables. For ease of presentation, the subscript *i*, representing the location, has been omitted (see Equation 1):

(3) $$\begin{array}{cc} {\text{RF}}_{>1960} & =\beta_{0}+\sum_{k=1}^{m}\beta_{{\text{clim}}_{k}}{\text{clim}}_{k}+\sum_{k=1}^{m}\beta_{\Delta {\text{clim}}_{k}}\Delta {\text{clim}}_{k}\\ & \;+\sum_{j=1}^{p}\beta_{{\text{LU}}_{j}}{\text{LU}}_{j}+\sum_{j=1}^{p}\beta_{\Delta {\text{LU}}_{j}}\Delta {\text{LU}}_{j}\\ & \;+\beta_{{\text{RF}}_{<1960}}{\text{RF}}_{<1960}+\beta_{W_{15}*{\text{RF}}_{<1960}}W_{15}*{\text{RF}}_{<1960}+\beta_{\text{Alt}}\text{Alt}+\varepsilon \end{array}$$

This equation describes the RF index from 1960 to 2016 (${\text{RF}}_{>1960}$) as a function of: (i) the RF index from 1849 to 1960 (${\text{RF}}_{<1960};\;\text{number}\;\text{of}\;\text{rain}\;\text{events}\;\text{following}\;\text{key}\;\text{day}$) and its spatially smoothed correspondent, $W_{15}*{\text{RF}}_{<1960}$, computed using a weighting scheme of the first 15 nearest observations ($W_{15}$); (ii) altitude (m), Alt; (iii) 1950 climatic variables, ${\text{Clim}}_{k}$ plus their seasonal change between 1950 and 2000, $\Delta {\text{Clim}}_{k}$ (temp °C; precipitation mm); and (iv) 1970 land‐use proportions, ${\text{LU}}_{j}$, and their change from 1970 and 2011, $\Delta {\text{LU}}_{j}$. As this study is both spatial and temporal, the relation with past observations for a given observation needed to account for both past and past near observations. The inclusion of the RF index from 1849 to 1960 (RF_<1960_) and its spatially smoothed correspondent controlled for potential spatio‐temporal autocorrelation with the RF index from 1960 to 2016 (${\text{RF}}_{>1960}$). In other words, we included the value of RF at the same location at time (*t* − 1) and the spatially smoothed values of RF at neighboring locations at time (*t* − 1) to explain the value of RF at a given location *x* at time *t*.

### 2.5 Model fitting and statistical analysis

Before fitting the GWR models to the summer and winter data, a feature selection step was performed using the maximum‐relevance minimum‐redundancy algorithm in order to identify a subset of both relevant and complementary features (De Jay et al., 2013). The coefficient parameters for the GWR models were estimated using the gwr function of the spgwr library in R, and their fit was compared with that for two OLS models. Local *T*‐values were generated to determine coefficient significance (1.96 ≤ *T* ≤ −1.96, *p* ≤ 0.05). Data extraction [sp (Pebesma, Bivand, Rowlingson, et al., 2020), sf (Pebesma, Bivand, Racine, et al., 2020), and raster (Hijmans et al., 2020)], feature selection [mlr (Bischl et al., 2016) and mRMRe (De Jay et al., 2013)], model fitting [spgwr (Bivand et al., 2020)], and plot generation [ggplot2 (Wickham, 2016)] were performed in RStudio 1.1.447 with R version 3.5.0.

## 3 RESULTS

### 3.1 Descriptive statistics for the region of study

Descriptive statistics for all variables used in the summer and winter GWR models are listed in Table S3. US landscapes in 1970 were dominated by agricultural and herbaceous land covers (48.6%) and mixed forest (20.6%; Table S3). The largest land‐use changes from 1970 to 2011 were agriculture and evergreen forests, which decreased an average of 6.1% and 1.6%, respectively, and developed land, which increased by 5.5%. Gains and losses for other land‐use types were <1% between 1970 and 2011. Since long‐term climate records tend to exist for weather stations in proximity to cities and agricultural regions, rather than natural areas, there is an inherent geographic bias in the data available. As such, caution should be applied to interpreting the results based on the land‐use estimates for regions of the United States where data are sparse and temporally limited (e.g., southwestern USA). Additionally, because there is not a weather or land‐use data set for the United States that covers the time period from 1849 to 2011, we had to use the PRISM data set (PRISM Gridded Climate Data, 2021) for periods before 1960 and the WorldClim data set (Fick & Hijmans, 2017) for periods after 1960. As such, it is difficult to predict how differences in the methods used to create these data sets may have affected the capacity of our models to differentiate independent variables that correlate to the RF index.

### 3.2 Geographically weighted regression model outputs and contribution of variables to RF

**FIGURE 1:**
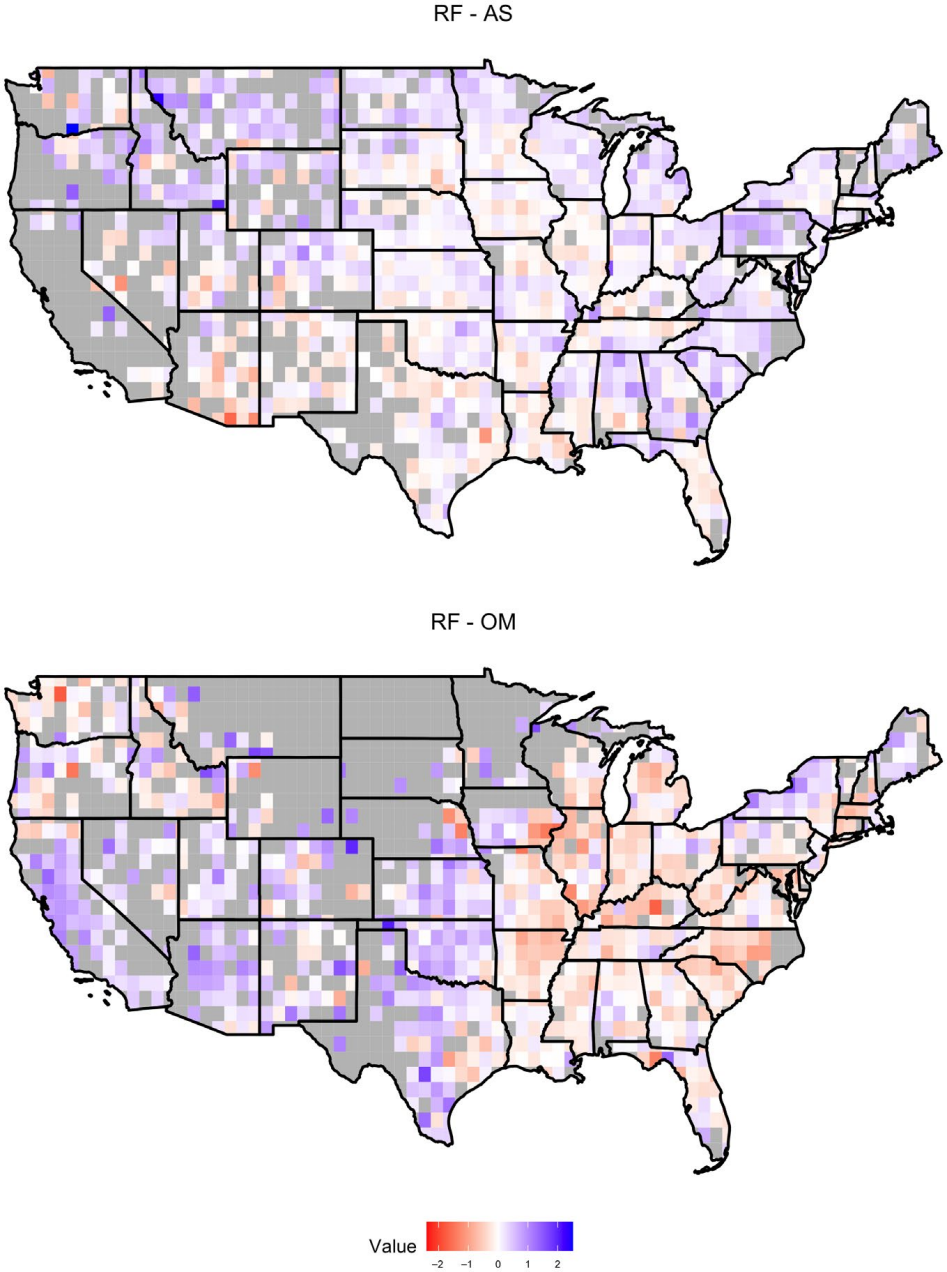
Rainfall feedback 1960–2010 for summer and winter

For all sites during the 1960–2016 period, the average RF index was positive in summer (0.11, median) and negative in winter (−0.024, median; Figure 1). RF index values for the Northern Rockies and the plains during winter could not be calculated due to lack of rainfall in these regions during that period. The coefficient of determination (*R* ^2^) averaged 0.40 for the entire period (Table S1) but varied by site and season (*R* ^2^ = 0.14 for AS, 0.38 for OM; Figure S1; Table S1). The RF index pre‐1960 did not correlate with post‐1960 RF, yet it improved the predictive power of the model (i.e., from a global *R* ^2^ of 0.39 to *R* ^2^ of 0.40 for the entire period).

Because the value of the GWR coefficients varies with location, their regression coefficients are less straightforward to interpret than those derived for conventional linear models. For this reason, the contribution of a predictor to the dependent variable was evaluated by examining the product of the coefficient and corresponding variable (Figures 3, 4, 5), which directly represents the value that each variable contributes to the predicted RF index at a given site.

#### 3.2.1 Effect of seasonal climate

**FIGURE 2:**
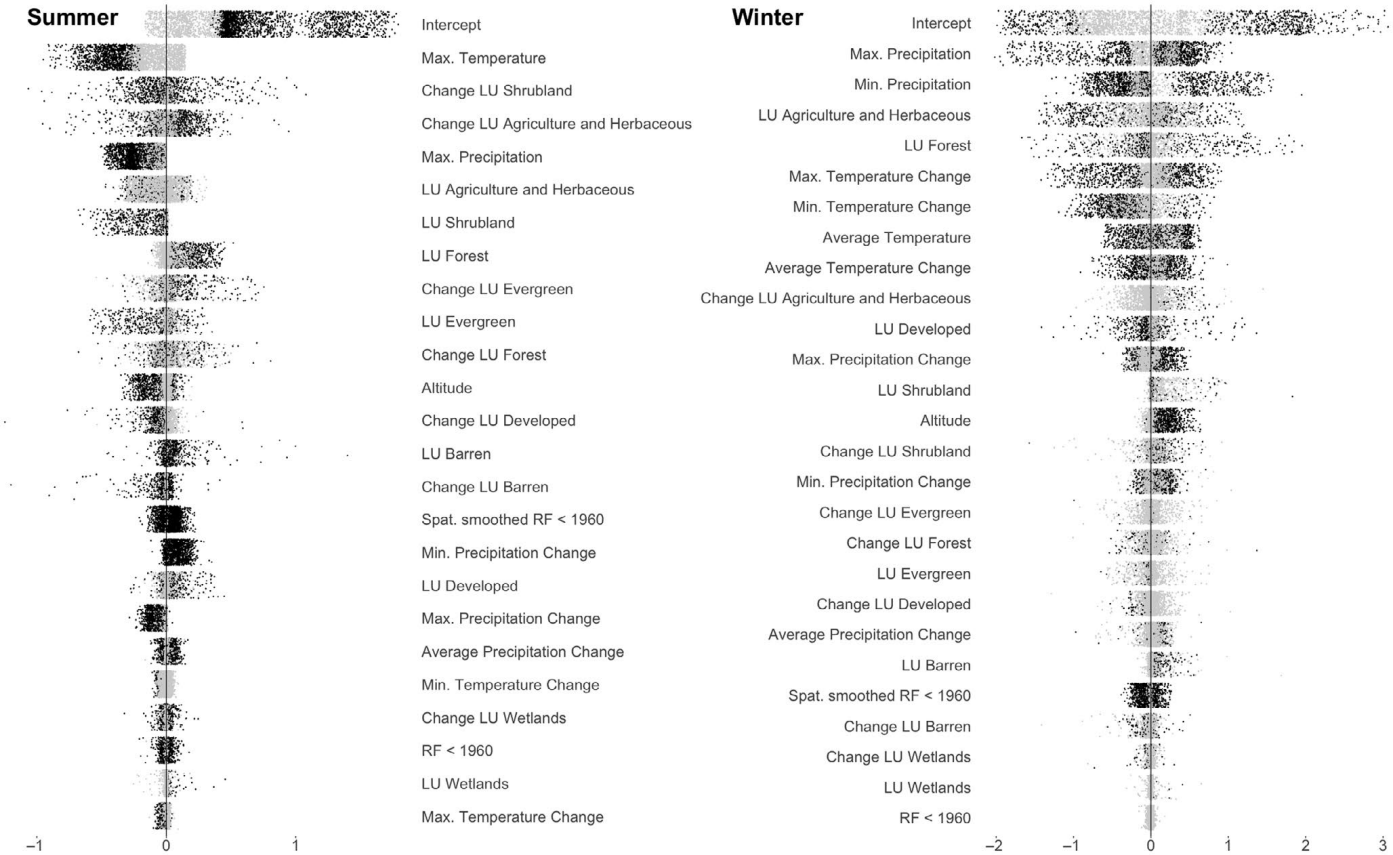
Effect of independent variables on RF for summer and winter. Horizontal axes represent the effect of the variable on RF (GWR coefficient × variable value). Black points correspond to which GWR coefficients were significantly different from 0, whereas gray points were non‐significant

**FIGURE 3:**
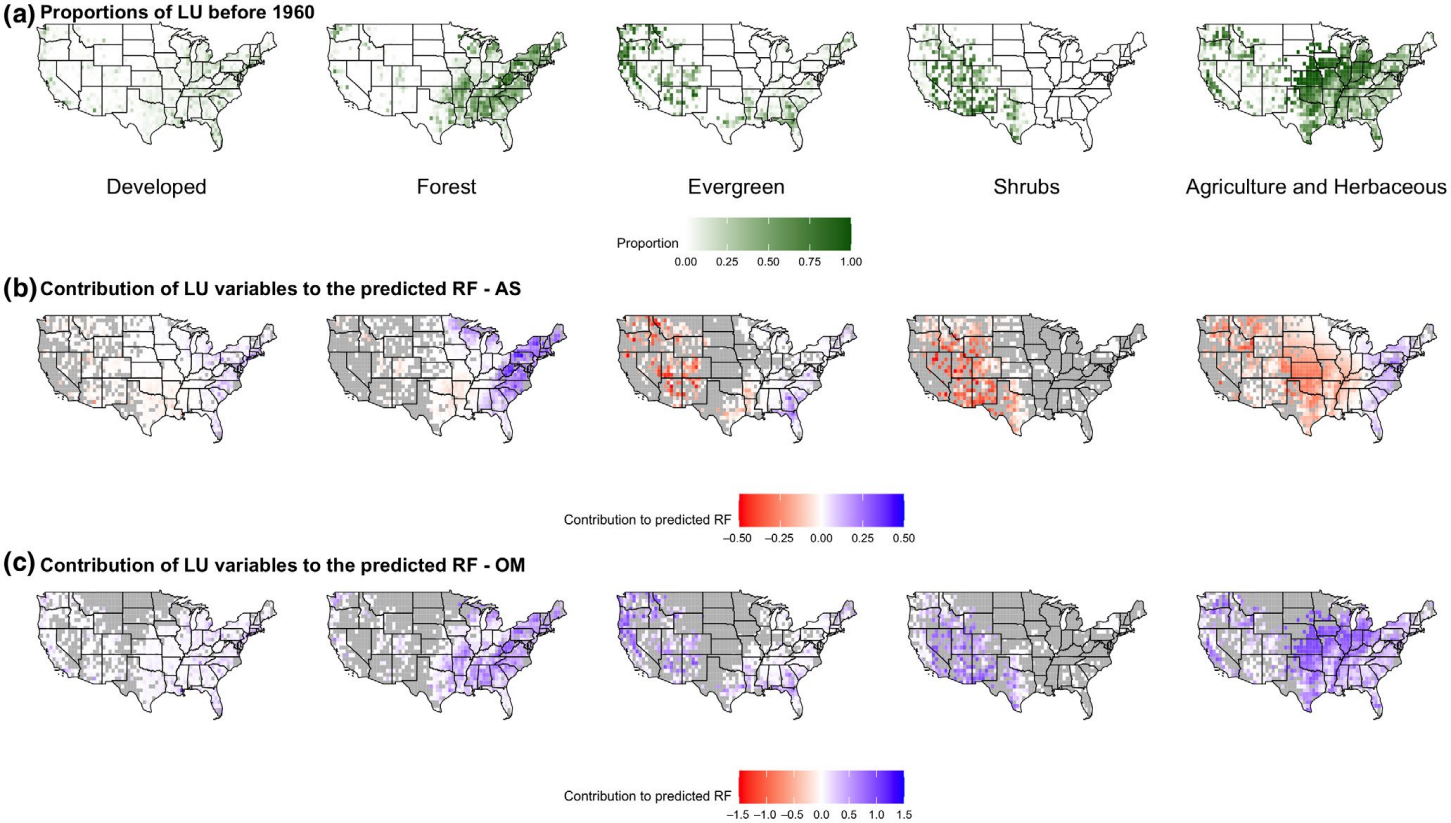
Contribution of non‐land‐use predictor variables to the RF index in summer and winter

The most strongly correlated and statistically significant predictors of the RF index are minimum precipitation change, maximum precipitation, and temperature (Figure 2; Supplemental files S1 and S2). In summer, maximum temperature, maximum precipitation, and maximum precipitation change are negatively correlated with the RF index for 83%, 100%, and 100%, respectively, of the sites (Figures 2 and 3; Table S4; Supplemental file S1). The period between April and September (i.e., summer) is markedly different from winter (Figures 2 and 3; Supplemental file S1), with most of the variables creating both positive and negative effects, depending on the site and its location (spatial nonstationarity).

#### 3.2.2 Effect of land use and land‐use change

**FIGURE 4:**
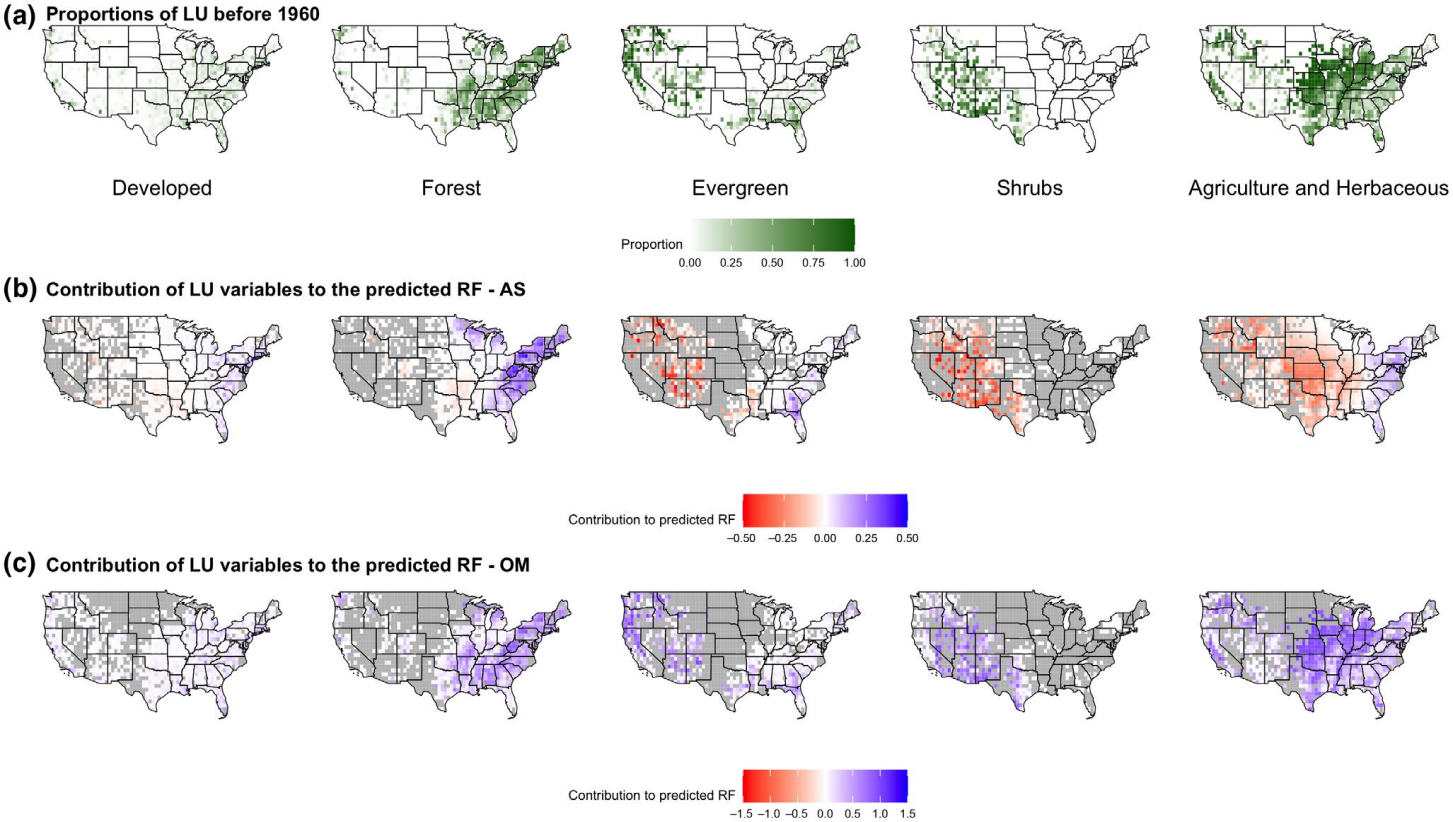
Response of RF to land‐use: proportion of that land‐use type by site (a), effect of that land‐use type in summer (b), and winter (c)

Of all the land‐use variables, forest, barren land, developed land, shrubland, and agriculture had the largest significant effects on summer and winter RF index values (Figure 2, Supplemental files S1 and S2). Fifty‐four percent of mixed forest sites had positive RF index values in summer, but less than half of these (21% of the sites) were statistically significant (Table S5; Supplemental file S1). Locations with a high proportion of developed land use (>20%) tended to coincide with positive RF index values, particularly along the east coast (Figure 4b; Table S5). Overall, effects associated with developed land use in summer were positive in the northeast and negative in the west and southwest (Figure 4). Shrubland (97% of sites overall), agriculture (66% of sites overall), and evergreen forest (69% of sites overall) negatively affected the RF index in the west and southwest regions (Figure 4), whereas evergreen forest produced significant positive effects in the southeast (Figure 4; Table S5; Supplemental file S1).

**FIGURE 5:**
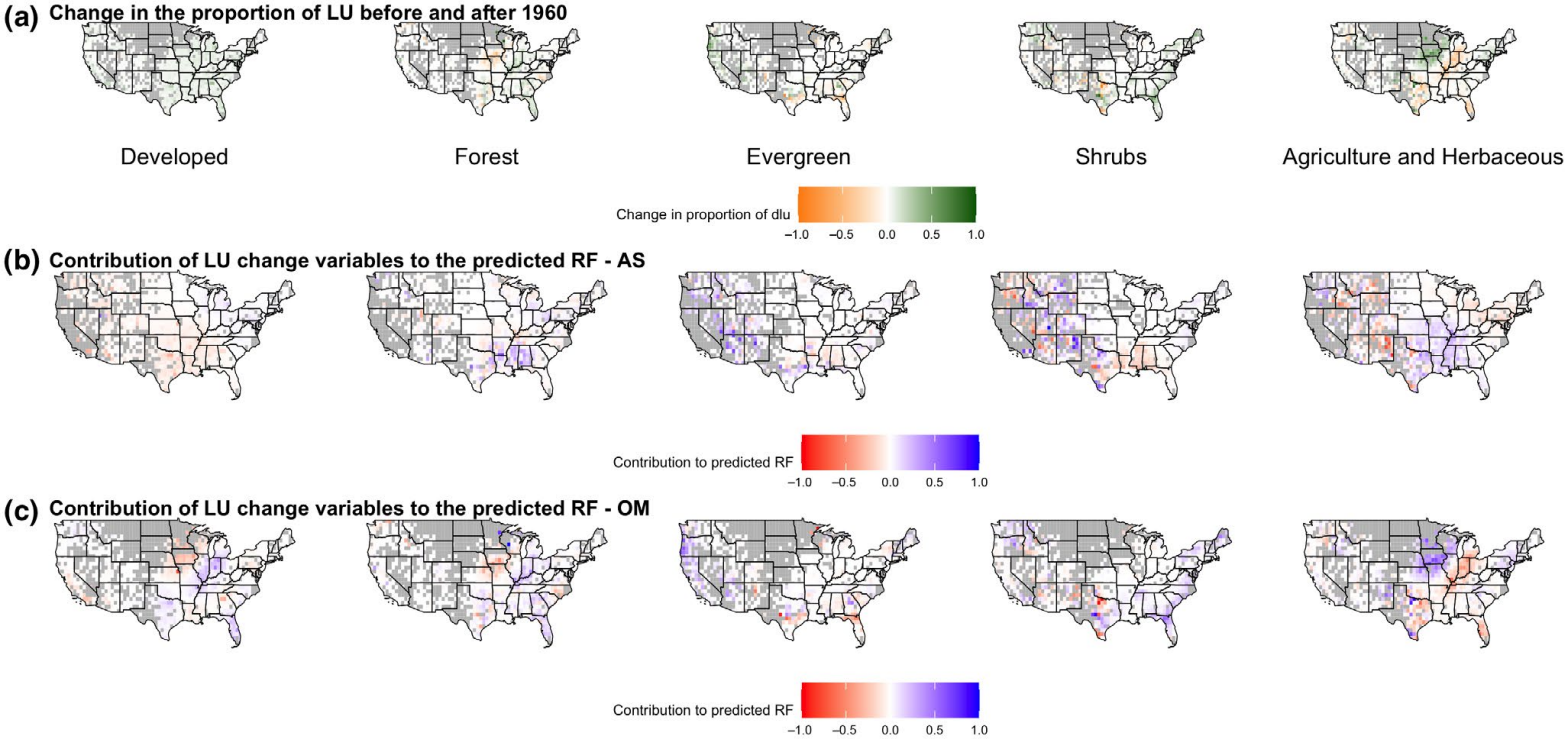
Response of RF to changes in land‐use from 1960 to 2010: proportional change of that land‐use type by site (a), effect land‐use change had in summer (b), and winter (c)

Land‐use change (i.e., change between 1970 and 2011) produced significant negative and positive effects on the RF index in summer (Table S6; Supplemental file S1). Decreases in forest were significantly positively correlated with the RF index in parts of the south (54% of sites positive, 22% significant; Figure 5; Table S6; Supplemental file S1). Decreases in evergreen forest in the western half of the United States significantly influenced the RF index positively (Figure 5; Table S6; Supplemental file S1). Changes in shrubland, agriculture, and developed land had significant effects on the RF index in the west (Figure 5; Table S6; Supplemental file S1), with 53% of sites associated with a negative effect in summer (Table S6; Supplemental file S1).

In winter, mixed forest sites in the Midwest and Southwest had a significant negative effect on the RF index (60% of sites, Figure 4c; Table S5; Supplemental file S2). Developed land use in winter also produced significant negative effects on RF in the Midwest and southeast (Figure 4c; Table S5; Supplemental file S2). Developed land, agriculture, and forest produced significant positive effects on the RF index in the northeast (Figure 4; Table S5; Supplemental file S2). Shrubland (70% of sites) and evergreen forest produced positive effects in winter for the southwest, but they were not statistically significant (Figure 4; Table S5; Supplemental file S2). Increases in developed land produced significant negative effects in the Midwest (53% of sites; Table S6; Supplemental file S2). Increases in shrubland and mixed forest in Florida produced significantly positive effects on the RF index (Figure 5; Table S6; Supplemental file S2).

## 4 DISCUSSION

Albedo, surface roughness, partitioning of latent and sensible heat, evapotranspiration, wind speed, and atmospheric turbulence are land‐surface properties well known to affect atmospheric water availability and temperature. Several studies have shown clear trends between soil moisture and rainfall probability (Taylor et al., 2011; Tuttle & Salvucci, 2016). Of the physical parameters likely to influence rainfall for periods of up to several weeks, feedback related to soil moisture remain a possible explanation. Short‐ and long‐term soil moisture memory has been documented to occur anywhere from 24‐h (Tuttle & Salvucci, 2016) to several months (Dirmeyer et al., 2009; Kumar et al., 2019; McColl et al., 2019; Nicolai‐Shaw et al., 2016). For instance, McColl et al. (2019) characterized short‐ and long‐term soil moisture memory using satellite observations. They found that soil moisture memory typically lasts for <10 days in the United States. Dirmeyer et al. (2009) found evidence for longer periods of soil moisture memory (~30–70 days). However, this was mainly positively correlated with evapotranspiration in the Great Plains during winter and spring months (i.e., December–February and March–May).

Bioaerosols produced as a result of rainfall have also been implicated in persistent RF where perceptible increases in cumulative precipitation events have occurred over a 3‐week period (Morris et al., 2017). Importantly, although trends consistent with feedback involving interactions between the landscape and atmosphere are readily discernable from climatic data analysis, our results do not provide information on the coupled abiotic‐biotic mechanisms amplifying these effects. Hence, the objective of this study is to provide the rationale for designing experiments that consider land use to test specific hypotheses on the physical and biological mechanisms that influence RF.

### 4.1 Potential biotic effects on rainfall feedback

There are multiple ways that biological processes could exert influence on meteorology and RF patterns. The response of organisms to environmental change and properties associated with the bioaerosols they produce provide opportunities for ecosystems to exert influence on synoptic conditions that promote or suppress rainfall. Increased moisture in plant and soil ecosystems from rain stimulates microbial growth (Hirano et al., 1996), and the emission of cloud‐active bioaerosol particles [i.e., giant cloud‐condensation nuclei (GCCN) and ice‐nucleating particles (INPs)] has been observed to increase over a several week period following rainfall (Bigg et al., 2015; Huffman et al., 2013; Iwata et al., 2019).

For example, increased moisture can facilitate the germination and growth of fungal rust urediospores, and Morris et al. (2013) showed that urediospores are effective INPs. Because urediospores are large (20–50 μm long‐axis diameter), hygroscopic particles, they also probably serve as efficient GCCN. Germination of urediospores triggered by elevated moisture and new spore production as part of its life cycle should occur over an interval of a week or two (Huffman et al., 2013; Teng & Close, 1978). Thus, rainfall may affect subsequent rainfall by enhancing physical conditions (e.g., increased soil moisture, evaporation, and rain splash; Anderegg et al., 2019; Eltahir, 1998) that can persist in the local environment for several days (Tuttle & Salvucci, 2016). Like GCCN, biological INPs with freeze‐catalyzing activities at >−15°C are rare aerosols (DeMott & Prenni, 2010; Murray et al., 2012), so rain‐induced changes in their atmospheric concentrations could have important meteorological consequences. In general, the more GCCN and INPs within a cloud, the more precipitation that is formed, and vice versa (DeMott et al., 2010). However, high concentrations of GCCN and INPs may form small cloud droplets and/or freeze a large portion of them, making the formation of precipitation less efficient (Fan et al., 2009; Khain et al., 2008).

Bigg et al. (2015) concluded that microbial life cycles influenced by moisture availability were the most probable explanation for the increase in the cumulative INP concentration of air after rain in the wheat belts of western and south‐eastern Australia (Bigg et al., 2015). In this scenario, positive RF patterns may result from conditions that promote biological productivity, growth, and subsequent emission of cloud‐active bioaerosols. Invoking biotic processes to explain negative RF is less straightforward since it could be the consequence of having too few or many cloud‐active particles in the atmosphere. Cloud‐seeding experiments have explored the effect of too many INPs for precipitation formation. For example, Neyman et al. (1969) observed reduced rainfall due to the reduced size of ice crystals caused by competition for water vapor, with many of the ice crystals evaporated before reaching the ground. A similar effect was observed in experiments attempting to enhance rain using the coalescence process by adding salt particles (Murty et al., 2000). When GCCN were added to relatively shallow clouds, the clouds dissipated due to fallout and evaporation of the many small drops produced.

Large increases in aerosols, including INPs and GCCNs, have been documented in boundary layer air from a North American forest ecosystem during and in the hours directly following rain (Huffman et al., 2013; Iwata et al., 2019; Prenni et al., 2013), but the increases are short‐lived (~1 to 12 h) compared with the timescale that RF is observed (Bigg et al., 2015; Morris et al., 2017). However, cumulative changes in INP concentration with durations of 20 days have been reported in regions of southeastern and southwestern Australia (Bigg et al., 2015). The timeframe for elevated emissions is in the range for observed RF indices following rain (Morris et al., 2017) and may be due to rainfall increasing soil moisture that fosters microbial growth in the source ecosystem. Although rainfall induces an observable biotic response with a duration from hours to days, more data are needed to better understand the temporal production of bioaerosols by different vegetation types following rain.

Biological processes are not easily incorporated into land–atmosphere models and are not typically represented, which has hampered understanding of the interactions and feedback between biota and climate. For example, plant physiological traits related to water transport have been shown to be important predictors of land‐surface feedback that affect drought intensification (Anderegg et al., 2019). However, studies that have examined plants as effectors of atmospheric conditions are limited to behaviors such as evapotranspiration or the release of volatile organic compounds (Anderegg et al., 2018; Franks et al., 2017; Kim, 2005) and have not considered the full spectrum of aerosols they emit. Previous studies hypothesized that RF is related to land‐use type and the cloud‐active bioaerosols emitted by the vegetation (Bigg et al., 2015; Morris et al., 2016, 2017). To disentangle tightly coupled processes in the land–atmosphere system and enhance understanding of the underlying biological interactions potentially at play, we used a modeling approach to identify the spatial influence of predictor variables that contribute to the observed patterns of RF (Figure 1). In addition, we provide a rationale to generate hypotheses for future studies that seek to identify the mechanisms that produce the RF patterns observed.

### 4.2 Explanatory variables with the largest influence on predicting rainfall feedback

Of the 24 explanatory variables examined at sites in the United States from 1960 to 2016 (Table S3), those with the largest effect on the RF index (Figure 2) were associated with temperature and precipitation. A decrease in maximum temperature change (∆ ~1°C) had a significant influence on negative RF in the southeast, potentially due to cooler summers that were also wetter on average (Figures 2 and 3; Supplemental file S1). This contrasts with observations of cooler average temperatures and wetter conditions in winter that generally corresponded with positive RF (Figure 2). The homeostatic tendency that extremes in precipitation have on the RF index, whereby increased aridity increases the probability for rainfall, and vice versa, is similar to observations in previous studies from Africa (Taylor et al., 2011) and other regions with relatively heterogenous soil moisture (Guillod et al., 2015; Hsu et al., 2017; Taylor et al., 2012; Zhou et al., 2021). The summer RF index was positively correlated to the minimum precipitation change and negatively correlated to maximum precipitation. However, in winter, lower values for minimum precipitation were associated with a positive effect on RF for most of the United States, with sites in southern states being the exception (Figures 2 and 3). Higher maximum precipitation values generally coincided with negative effects on RF (Figure 2), except for the northeast. This could be due to the increase in soil moisture following rainfall, which alters the surface energy balance, dampening atmospheric instability, and inhibiting the vertical movement of air parcels (Cook et al., 2006; Tuttle & Salvucci, 2016). However, this is unlikely to be sole explanation as such conditions do not persist for the upper‐bound duration of observed RF (~20 days; McColl et al., 2019). Nevertheless, our results indicate that precipitation can create unsuitable conditions for future rainfall, similar to the observations of Yang et al. (2018) for certain land types, locations, and seasons.

Although differences in temperature and precipitation can partially explain the dependence of RF on season, the relationships between land‐use variables on RF vary across space (i.e., intrinsic spatial nonstationarity; Figures 2, 3, 4, 5), implying land‐use drivers that operate at smaller spatial and/or temporal scales than the modelled climate. Plants influence atmospheric processes in multiple and dynamic ways (Hartley et al., 2016). For instance, plant physiological responses to water stress that decrease rates of evapotranspiration influence local weather (Anderegg et al., 2018, 2019). And as atmospheric sources of cloud‐active bioaerosols (Ziemba et al., 2016), vegetated landscapes also have the potential to influence cloud development and precipitation (Morris et al., 2014). In this study, mixed forest, developed land use, and agricultural biomes had a positive influence on the summer RF index, whereas it was negatively affected at shrub and evergreen forest sites (Figure 4a,b). The trends for the effect of land‐use variables on RF were similar to those for changes in land use (between 1970 and 2011). For example, the modest increase in area of evergreen and mixed forest during this period (0.97% and 4.3%, respectively) nevertheless produced a positive and negative effect on RF, respectively (Figure 5; Table S6). Conifer leaves have not been shown to be a significant source of microbial INPs (Lindow et al., 1978); however, conifer pollen can serve as CCN and are also associated with ice nucleation active macromolecules (Amato et al., 2007; Pummer et al., 2012; Steiner et al., 2015). Hence, this possibility means that a biotic response by the vegetation itself (e.g., pollen formation), and not just members of its associated microbiome, may have direct involvement in atmospheric processes that effect RF.

The influence of agriculture on the summer RF index from 1960–2010 was generally positive on the east coast and negative west of the Mississippi River (Figure 4b). Most of the cropland area is located in the western United States (USDA & NASS, 2012; sites with >60% agricultural land use; Figure 4a), and the RF index regression coefficients negatively correlate with the total proportion of land used for agriculture (Figure S2). This observation suggests that more expansive and contiguous farms may influence land–atmosphere interactions in ways that reduce rainfall frequency in summer and that there is potentially a threshold effect. Western sites in agricultural regions had a positive influence on RF in winter, while those from the Midwestern Corn Belt to the east coast produced a negative effect (Figure 4c). Intriguingly, the negative effect overlaps with the cultivation of soft red winter wheat in the Midwest. Since the early 1900s, crops in the wheat belt, and in particular in the Midwest, suffer regular epidemics of disease caused by the rust fungi *Puccinia* (Eversmeyer & Kramer, 2000; Hamilton & Stakman, 1967). Urediospores of rusts are ice nucleation active at relatively warm temperatures (warmer than −10°C) and specifically adapted for aerial dissemination and transport over long distances at altitudes of several kilometers (Amato et al., 2005; Bauer et al., 2002; Bowers et al., 2009). Rusts are capable of co‐transporting microorganisms that inhabit the surfaces of wheat leaves and remain attached to the spores, including ice nucleation active strains of *Pseudomonas syringae* (Morris et al., 2013). The positive effects in the south and northeast coincide with the end of the growing season and loss of crop canopy (Figure 4c). These results indicate that certain land‐use types are significant predictors of RF at particular sites, with the influence of season and associated changes in vegetation strongly implicating a role for plant ecosystems in this interaction.

In addition to the monocultures associated with agricultural land use, natural and managed forest ecosystems also represent large sources of aerosolized microbial INPs and GCCN (Garcia et al., 2012; Huffman et al., 2013; Suski et al., 2018). Developed regions associated with forests (Nowak et al., 2001) (i.e., urban forest) have similar canopy coverage to that of mixed forest (U.S. Department of Agriculture, 2012). Urban forests in the south and northeast US coast with a high percentage of tree cover (>50%; Figure 4) coincided with locations that had positive effects on RF during the summer (Figure 4b).

A key observation from this study is the unearthing of a potential threshold effect on the RF index for developed land use. Sites with >40% development were positively correlated with the RF index in summer (Figure S3), whereas those with <40% were negatively correlated. In summer, the stimulation of growth and emission of bioaerosols from the urban forest canopy following rain could promote sufficient atmospheric fluxes of GCCN and/or INPs, thus contributing to an effect on RF (Huffman et al., 2013). When the deciduous canopy is absent in winter, the flux of aerosolized particles from the ground surface becomes the more dominant aerosol source to the atmosphere (Nguyen et al., 2015). The RF effect change in winter is consistent with a lack of leaves as a source and with plant processes that differ between growing and dormant seasons. These results provide motivation to generate new hypotheses, that when tested, can aid in better understanding the dynamics of land–vegetation–atmosphere interactions that affect rainfall frequency.

## 5 CONCLUSIONS

Analysis of historical data sets were used to appraise the role of land use and climate on RF patterns in the United States during the last three centuries. The results provide a context for designing field‐based studies that aim to determine the biotic and abiotic contributions to feedback that influence rainfall frequency. Based on this approach, we infer that climate and land use are the most important predictors of patterns in RF observed in the United States since 1960. The tendency of landscapes to have negative or positive effects on RF is dependent on the type of land use, proportion of land‐use type, and season (i.e., summer vs. winter). Although the way in which landscapes and their terrestrial biota exert specific control remains obscure, alteration of the emission of cloud‐active bioaerosols from sources associated with vegetation and soil ecosystems provides one plausible explanation for the patterns observed with changes in land use and season. Given that RF patterns are widely observable, but the underlying mechanisms are currently speculative, a fundamental understanding of the abiotic–biotic processes involved warrants more detailed investigation. For example, studies that examine the temporal effect of rainfall events on soil moisture memory versus bioaerosol emission fluxes from the different land‐use types and sites identified in this study (Figure 4) could provide the data needed to evaluate the contribution of physical versus biological mechanisms. Improving understanding of the linkages between RF, terrestrial biomes, and the atmospheric processes that affect rainfall provides the context necessary for predicting these behaviors in the 21st century climate system and for managing land use in ways that optimize water use and contribute to food security.

## CONFLICT OF INTEREST

The authors declare no conflict of interest.

## Supporting information

Fig S1

Fig S2

Fig S3

Table S1‐6

Supplementary Material

Supplementary Material

## Data Availability

The data that support the findings of this study are available at w3.avignon.inra.fr/rainfallfeedback/, mrlc.gov/data/nlcd‐2011‐land‐cover‐conus‐0, prism.oregonstate.edu/, worldclim.org/version2, https://doi.org/10.1890/13‐1245.1, and ipcc‐data.org/sim/gcm_monthly/SRES_TAR/index.html.

## References

[gcb15876-bib-0001] Amato, P. , Ménager, M. , Sancelme, M. , Laj, P. , Mailhot, G. , & Delort, A. M. (2005). Microbial population in cloud water at the Puy de Dôme: Implications for the chemistry of clouds. Atmospheric Environment, 39(22), 4143–4153. 10.1016/j.atmosenv.2005.04.002

[gcb15876-bib-0002] Amato, P. , Parazols, M. , Sancelme, M. , Laj, P. , Mailhot, G. , & Delort, A. M. (2007). Microorganisms isolated from the water phase of tropospheric clouds at the Puy de Dôme: Major groups and growth abilities at low temperatures. FEMS Microbiology Ecology, 59, 242–254. 10.1111/j.1574-6941.2006.00199.x 17328765

[gcb15876-bib-0003] Anderegg, W. R. L. , Konings, A. G. , Trugman, A. T. , Yu, K. , Bowling, D. R. , Gabbitas, R. , Karp, D. S. , Pacala, S. , Sperry, J. S. , Sulman, B. N. , & Zenes, N. (2018). Hydraulic Diversity of Forests Regulates Ecosystem Resilience during Drought. Nature. Nature Publishing Group, 10.1038/s41586-018-0539-7. 30232452

[gcb15876-bib-0004] Anderegg, W. R. L. , Trugman, A. T. , Bowling, D. R. , Salvucci, G. , & Tuttle, S. E. (2019). Plant functional traits and climate influence drought intensification and land–atmosphere feedbacks. Proceedings of the National Academy of Sciences of the United States of America, 116(28), 14071–14076. 10.1073/pnas.1904747116 31235581 PMC6628816

[gcb15876-bib-0005] Andreae, M. O. , & Rosenfeld, D. (2008). Aerosol–cloud–precipitation interactions. Part 1. The nature and sources of cloud‐active aerosols. Earth‐Science Reviews, 89(1‐2), 13–41. 10.1016/j.earscirev.2008.03.001

[gcb15876-bib-0006] Bauer, H. , Kasper‐Giebl, A. , Löflund, M. , Giebl, H. , Hitzenberger, R. , Zibuschka, F. , & Puxbaum, H. (2002). The contribution of bacteria and fungal spores to the organic carbon content of cloud water, precipitation and aerosols. Atmospheric Research, 64(1–4), 109–119. 10.1016/S0169-8095(02)00084-4

[gcb15876-bib-0007] Bigg, E. K. , Soubeyrand, S. , & Morris, C. E. (2015). Persistent after‐effects of heavy rain on concentrations of ice nuclei and rainfall suggest a biological cause. Atmospheric Chemistry and Physics, 15, 2313–2326. 10.5194/acp-15-2313-2015

[gcb15876-bib-0008] Bischl, B. , Lang, M. , Kotthoff, L. , Schiffner, J. , Richter, J. , Studerus, E. , Casalicchio, G. , & Jones, Z. M. (2016). Mlr: Machine learning in R. Journal of Machine Learning Research, 17. https://github.com/mlr‐org/mlr

[gcb15876-bib-0009] Bivand, R. , Danlin, Y. U. , Nakaya, T. , & Garcia‐Lopez, M.‐A. (2020). Geographically weighted regression [R package Spgwr]. Comprehensive R Archive Network (CRAN). https://cran.r‐project.org/package=spgwr

[gcb15876-bib-0010] Bivand, R. , Danlin, Y. U. , Tomoki, N. , & Miquel‐Angel, G.‐L. (2017). Geographically weighted regression [R package Spgwr version 0.6‐32]. https://cran.r‐project.org/package=spgwr

[gcb15876-bib-0011] Bowers, R. M. , Lauber, C. L. , Wiedinmyer, C. , Hamady, M. , Hallar, A. G. , Fall, R. , Knight, R. , & Fierer, N. (2009). Characterization of airborne microbial communities at a high‐elevation site and their potential to act as atmospheric ice nuclei. Applied and Environmental Microbiology, 75(15), 5121–5130. 10.1128/AEM.00447-09 19502432 PMC2725505

[gcb15876-bib-0012] Chambers, J. Q. , & Artaxo, P. (2017). Deforestation size influences rainfall. Nature Climate Change, 7(February), 175–176. 10.1038/nclimate3238

[gcb15876-bib-0013] Cook, B. I. , Bonan, G. B. , & Levis, S. (2006). Soil moisture feedbacks to precipitation in southern Africa. Journal of Climate, 19(17), 4198–4206. 10.1175/JCLI3856.1

[gcb15876-bib-0014] De Jay, N. , Papillon‐Cavanagh, S. , Olsen, C. , El‐Hachem, N. , Bontempi, G. , & Haibe‐Kains, B. (2013). MRMRe: An R package for parallelized MRMR ensemble feature selection. Bioinformatics, 29(18), 2365–2368. 10.1093/bioinformatics/btt383 23825369

[gcb15876-bib-0015] de Noblet‐Ducoudré, N. , Boisier, J.‐P. , Pitman, A. , Bonan, G. B. , Brovkin, V. , Cruz, F. , Delire, C. , Gayler, V. , van den Hurk, B. J. J. M. , Lawrence, P. J. , van der Molen, M. K. , Müller, C. , Reick, C. H. , Strengers, B. J. , & Voldoire, A. (2012). Determining robust impacts of land‐use‐induced land cover changes on surface climate over North America and Eurasia: Results from the first set of LUCID experiments. Journal of Climate, 25(9), 3261–3281. 10.1175/JCLI-D-11-00338.1

[gcb15876-bib-0016] DeMott, P. J. , & Prenni, A. J. (2010). New directions: Need for defining the numbers and sources of biological aerosols acting as ice nuclei. Atmospheric Environment, 44(15), 1944–1945. 10.1016/J.ATMOSENV.2010.02.032

[gcb15876-bib-0017] DeMott, P. J. , Prenni, A. J. , Liu, X. , Kreidenweis, S. M. , Petters, M. D. , Twohy, C. H. , Richardson, M. S. , Eidhammer, T. , & Rogers, D. C. (2010). Predicting global atmospheric ice nuclei distributions and their impacts on climate. Proceedings of the National Academy of Sciences of the United States of America, 107(25), 11217–11222. 10.1073/pnas.0910818107 20534566 PMC2895116

[gcb15876-bib-0018] Denevan, W. M. (1992). The pristine myth: The landscape of the Americas in 1492. Annals of the Association of American Geographers, 82(3), 369–385. 10.1111/j.1467-8306.1992.tb01965.x

[gcb15876-bib-0019] Dirmeyer, P. A. , Adam Schlosser, C. , & Brubaker, K. L. (2009). Precipitation, recycling, and land memory: An integrated analysis. Journal of Hydrometeorology, 10(1), 278–288. 10.1175/2008JHM1016.1

[gcb15876-bib-0020] Eltahir, E. A. B. (1998). A soil moisture‐rainfall feedback mechanism 1. Theory and observations. Water Resources Research, 34(4), 765–776. 10.1029/97WR03499

[gcb15876-bib-0021] Eversmeyer, M. G. , & Kramer, C. L. (2000). Epidemiology of wheat leaf and stem rust in the central great plains of the USA. Annual Review of Phytopathology, 38(1), 491–513. 10.1146/annurev.phyto.38.1.491 11701852

[gcb15876-bib-0022] Fan, J. , Yuan, T. , Comstock, J. M. , Ghan, S. , Alexander Khain, L. , Leung, R. , Li, Z. , Martins, V. J. , & Ovchinnikov, M. (2009). Dominant role by vertical wind shear in regulating aerosol effects on deep convective clouds. Journal of Geophysical Research, 114(D22), D22206. 10.1029/2009JD012352

[gcb15876-bib-0023] Fick, S. E. , & Hijmans, R. J. (2017). Worldclim 2: New 1‐km spatial resolution climate surfaces for global land areas. International Journal of Climatology, 37, 4302–4315. 10.1002/joc.5086

[gcb15876-bib-0024] Findell, K. L. , Berg, A. , Gentine, P. , Krasting, J. P. , Lintner, B. R. , Malyshev, S. , Santanello, J. A. , & Shevliakova, E. (2017). The impact of anthropogenic land use and land cover change on regional climate extremes. Nature Communications, 8(1), 10.1038/s41467-017-01038-w PMC5651924 29057878

[gcb15876-bib-0025] Findell, K. L. , Gentine, P. , Lintner, B. R. , & Kerr, C. (2011). Probability of afternoon precipitation in eastern United States and Mexico enhanced by high evaporation. Nature Geoscience, 4(7), 434–439. 10.1038/ngeo1174

[gcb15876-bib-0026] Fotheringham, A. S. , Brunsdon, C. , & Charlton, M. (2002). Geographically weighted regression: The analysis of spatially varying relationships. Wiley. https://books.google.com/books/about/Geographically_Weighted_Regression.html?id=9DZgV1vXOuMC&printsec=frontcover&source=kp_read_button#v=onepage&q&f=false

[gcb15876-bib-0027] Franks, P. J. , Berry, J. A. , Lombardozzi, D. L. , & Bonan, G. B. (2017). Stomatal function across temporal and spatial scales: Deep‐time trends, land‐atmosphere coupling and global models. Plant Physiology, 174(2), 583–602. 10.1104/pp.17.00287 28446638 PMC5462067

[gcb15876-bib-0028] Garcia, E. , Hill, T. C. J. , Prenni, A. J. , DeMott, P. J. , Franc, G. D. , & Kreidenweis, S. M. (2012). Biogenic ice nuclei in boundary layer air over two U.S. high plains agricultural regions. Journal of Geophysical Research: Atmospheres, 117(D18), n/a‐n/a. 10.1029/2012JD018343

[gcb15876-bib-0029] Gerken, T. , Bromley, G. T. , & Stoy, P. C. (2018). Surface moistening trends in the northern North American great plains increase the likelihood of convective initiation. Journal of Hydrometeorology, 19(1), 227–244. 10.1175/JHM-D-17-0117.1

[gcb15876-bib-0030] Goldewijk, K. , Kees, A. B. , Van Drecht, G. , & De Vos, M. (2011). The HYDE 3.1 spatially explicit database of human‐induced global land‐use change over the past 12,000 years. Global Ecology and Biogeography, 20(1), 73–86. 10.1111/j.1466-8238.2010.00587.x

[gcb15876-bib-0031] Guillod, B. P. , Orlowsky, B. , Miralles, D. G. , Teuling, A. J. , & Seneviratne, S. I. (2015). Reconciling spatial and temporal soil moisture effects on afternoon rainfall. Nature Communications 2015, 6(1), 1–6. 10.1038/ncomms7443 PMC4366536 25740589

[gcb15876-bib-0032] Hamilton, L. M. , & Stakman, E. C. (1967). Time of stem rust appearance on wheat in the western Mississippi basin in relation to the development of epidemics from 1921 to 1962. Phytopathology, 57(6), 609–614. https://www.cabdirect.org/cabdirect/abstract/19671103048

[gcb15876-bib-0033] Hartley, A. J. , Parker, D. J. , Garcia‐Carreras, L. , & Webster, S. (2016). Simulation of vegetation feedbacks on local and regional scale precipitation in west Africa. Agricultural and Forest Meteorology, 222(May), 59–70. 10.1016/J.AGRFORMET.2016.03.001

[gcb15876-bib-0034] Hijmans, R. J. , van Etten, J. , Sumner, M. , Cheng, J. , Baston, D. , Bevan, A. , Bivand, R. , Busetto, L. , Canty, M. , Fasoli, B. , Forrest, B. , Ghosh, A. , Golicher, D. , Gray, J. , Greenberg, J. A. , Hiemstra, P. , Hingee, K. , Karney, C. , & Institute for Mathematics Applied Geosciences . (2020). Geographic data analysis and modeling [R package raster]. Comprehensive R Archive Network (CRAN). https://cran.r‐project.org/package=raster

[gcb15876-bib-0035] Hirano, S. S. , Baker, L. S. , & Upper, C. D. (1996). Raindrop momentum triggers growth of leaf‐associated populations of *Pseudomonas syringae* on field‐grown snap bean plants. Applied and Environmental Microbiology, 62(7), 2560–2566. 10.1128/aem.62.7.2560-2566.1996 16535362 PMC1388900

[gcb15876-bib-0036] Hsu, H. , Lo, M.‐H. , Guillod, B. P. , Miralles, D. G. , & Kumar, S. (2017). Relation between precipitation location and antecedent/subsequent soil moisture spatial patterns. Journal of Geophysical Research: Atmospheres, 122(12), 6319–6328. 10.1002/2016JD026042

[gcb15876-bib-0037] Huffman, J. A. , Prenni, A. J. , DeMott, P. J. , Pöhlker, C. , Mason, R. H. , Robinson, N. H. , Fröhlich‐Nowoisky, J. , Tobo, Y. , Després, V. R. , Garcia, E. , Gochis, D. J. , Harris, E. , Müller‐Germann, I. , Ruzene, C. , Schmer, B. , Sinha, B. , Day, D. A. , Andreae, M. O. , Jimenez, J. L. , … Pöschl, U. (2013). High concentrations of biological aerosol particles and ice nuclei during and after rain. Atmospheric Chemistry and Physics, 13(13), 6151–6164. 10.5194/acp-13-6151-2013

[gcb15876-bib-0038] Iwata, A. , Imura, M. , Hama, M. , Maki, T. , Tsuchiya, N. , Kunihisa, R. , & Matsuki, A. (2019). Release of highly active ice nucleating biological particles associated with rain. Atmosphere, 10(10), 605. 10.3390/atmos10100605

[gcb15876-bib-0039] Khain, A. P. , BenMoshe, N. , & Pokrovsky, A. (2008). Factors determining the impact of aerosols on surface precipitation from clouds: An attempt at classification. Journal of the Atmospheric Sciences, 65(6), 1721–1748. 10.1175/2007JAS2515.1

[gcb15876-bib-0040] Khanna, J. , Medvigy, D. , Fueglistaler, S. , & Walko, R. (2017). Regional dry‐season climate changes due to three decades of Amazonian deforestation. Nature Climate Change, 7(3), 200–204. 10.1038/nclimate3226

[gcb15876-bib-0041] Kim, Y. (2005). Modeling seasonal vegetation variation and its validation against moderate resolution imaging spectroradiometer (MODIS) observations over north America. Journal of Geophysical Research, 110(D4), D04106. 10.1029/2004JD005436

[gcb15876-bib-0042] Kumar, S. , Newman, M. , Wang, Y. , & Livneh, B. (2019). Potential reemergence of seasonal soil moisture anomalies in north America. Journal of Climate, 32(10), 2707–2734. 10.1175/JCLI-D-18-0540.1

[gcb15876-bib-0043] Lejeune, Q. , Davin, E. L. , Gudmundsson, L. , Winckler, J. , & Seneviratne, S. I. (2018). Historical deforestation locally increased the intensity of hot days in northern mid‐latitudes. Nature Climate Change, 8(5), 386–390. 10.1038/s41558-018-0131-z

[gcb15876-bib-0044] Li, G. , Zhang, F. , Jing, Y. , Liu, Y. , & Sun, G. E. (2017). Response of evapotranspiration to changes in land use and land cover and climate in China during 2001–2013. Science of the Total Environment, 596–597(October), 256–265. 10.1016/j.scitotenv.2017.04.080 28433768

[gcb15876-bib-0045] Lindow, S. E. , Arny, D. C. , & Upper, C. D. (1978). Distribution of ice nucleation‐active bacteria on plants in nature. Applied and Environmental Microbiology, 36(6), 831–838. 10.1128/aem.36.6.831-838.1978 736541 PMC243154

[gcb15876-bib-0046] McColl, K. A. , He, Q. , Hui, L. U. , & Entekhabi, D. (2019). Short‐term and long‐term surface soil moisture memory time scales are spatially anticorrelated at global scales. Journal of Hydrometeorology, 20(6), 1165–1182. 10.1175/JHM-D-18-0141.1

[gcb15876-bib-0047] Morris, C. E. , Franz Conen, J. , Huffman, A. , Phillips, V. , Pöschl, U. , & Sands, D. C. (2014). Bioprecipitation: A feedback cycle linking earth history, ecosystem dynamics and land use through biological ice nucleators in the atmosphere. Global Change Biology, 20(2), 341–351. 10.1111/gcb.12447 24399753

[gcb15876-bib-0048] Morris, C. E. , Georgakopoulos, D. G. , & Sands, D. C. (2004). Ice nucleation active bacteria and their potential role in precipitation. Journal de Physique IV (Proceedings), 121(December), 87–103. 10.1051/jp4:2004121004

[gcb15876-bib-0049] Morris, C. E. , Samuel Soubeyrand, E. , Bigg, K. , Creamean, J. M. , & Sands, D. C. (2016). A framework to assess the contribution of bioaerosols to the outcome of meteorological contexts favorable for rainfall. BioRxiv, August, 070532. 10.1101/070532

[gcb15876-bib-0050] Morris, C. E. , Samuel Soubeyrand, E. , Bigg, K. , Creamean, J. M. , Sands, D. C. , Morris, C. E. , Samuel Soubeyrand, E. , Bigg, K. , Creamean, J. M. , & Sands, D. C. (2017). Mapping rainfall feedback to reveal the potential sensitivity of precipitation to biological aerosols. Bulletin of the American Meteorological Society, 98(6), 1109–1118. 10.1175/BAMS-D-15-00293.1

[gcb15876-bib-0051] Morris, C. E. , Sands, D. C. , Glaux, C. , Samsatly, J. , Asaad, S. , Moukahel, A. R. , Gonçalves, F. L. T. , & Bigg, E. K. (2013). Urediospores of rust fungi are ice nucleation active at > −10°C and harbor ice nucleation active bacteria. Atmospheric Chemistry and Physics, 13(8), 4223–4233. 10.5194/acp-13-4223-2013

[gcb15876-bib-0052] Murray, B. J. , O’Sullivan, D. , Atkinson, J. D. , & Webb, M. E. (2012). Ice nucleation by particles immersed in supercooled cloud droplets. Chemical Society Reviews, 41(19), 6519. 10.1039/c2cs35200a 22932664

[gcb15876-bib-0053] Murty, A. S. R. , Selvam, A. M. , Devara, P. C. S. , Krishna, K. , Chatterjee, R. N. , Mukherjee, B. K. , Khemani, L. T. , Momin, G. A. , Reddy, R. S. , Sharma, S. K. , Jadhav, D. B. , Vijayakumar, R. , Raj, P. E. , Manohar, G. K. , Kandalgaonkar, S. S. , Paul, S. K. , Pillai, A. G. , Parasnis, S. S. , Londhe, A. L. , … Tinmaker, M. I. R. (2000). 11‐Year warm cloud seeding experiment in Maharashtra State, India. The Journal of Weather Modification, 32(1), 10–20. http://journalofweathermodification.org/index.php/JWM/article/view/239

[gcb15876-bib-0054] Nair, U. S. , Wu, Y. , Kala, J. , Lyons, T. J. , Pielke, R. A. , & Hacker, J. M. (2011). The role of land use change on the development and evolution of the west coast trough, convective clouds, and precipitation in southwest Australia. Journal of Geophysical Research Atmospheres, 116(7), D07103. 10.1029/2010JD014950

[gcb15876-bib-0055] Neyman, J. , Scott, E. , & Smith, J. A. (1969). Areal spread of the effect of cloud seeding at the whitetop experiment. Science, 163(3874), 1445–1449. 10.1126/science.163.3874.1445 17840325

[gcb15876-bib-0056] Nguyen, T. , Xinxiao, Y. U. , Zhang, Z. , Liu, M. , & Liu, X. (2015). Relationship between types of urban forest and PM2.5 capture at three growth stages of leaves. Journal of Environmental Sciences, 27(January), 33–41. 10.1016/j.jes.2014.04.019 25597660

[gcb15876-bib-0057] Nicolai‐Shaw, N. , Gudmundsson, L. , Hirschi, M. , & Seneviratne, S. I. (2016). Long‐term predictability of soil moisture dynamics at the global scale: Persistence versus large‐scale drivers. Geophysical Research Letters, 43(16), 8554–8562. 10.1002/2016GL069847

[gcb15876-bib-0058] Nowak, D. J. , Noble, M. H. , Sisinni, S. M. , & Dwyer, J. F. (2001). Assessing the US urban forest resources. Journal of Forestry, 99(3), 37–42.

[gcb15876-bib-0059] Pathirana, A. , Denekew, H. B. , Veerbeek, W. , Zevenbergen, C. , & Banda, A. T. (2014). Impact of urban growth‐driven landuse change on microclimate and extreme precipitation—A sensitivity study. Atmospheric Research, 138(March), 59–72. 10.1016/j.atmosres.2013.10.005

[gcb15876-bib-0060] Pebesma, E. , Bivand, B. , Rowlingson, B. , Gomez‐Rubio, V. , Hijmans, R. , Sumner, M. , MacQueen, D. , Lemon, J. , Obrien, J. , & Orourke, J. (2020). Classes and Methods for Spatial Data [R Package Sp]. Comprehensive R Archive Network (CRAN). https://cran.r‐project.org/package=sp

[gcb15876-bib-0061] Pebesma, E. , Bivand, R. , Racine, E. , Sumner, M. , Cook, I. , Keitt, T. , Lovelace, R. , Wickham, H. , Ooms, J. , Müller, K. , Pedersen, T. L. , Baston, D. , & Dunnington, D. (2020). Simple features for R [R package Sf]. Comprehensive R Archive Network (CRAN). https://cran.r‐project.org/package=sf

[gcb15876-bib-0062] Pitman, A. J. , Avila, F. B. , Abramowitz, G. , Wang, Y. P. , Phipps, S. J. , & De Noblet‐Ducoudré, N. (2011). Importance of background climate in determining impact of land‐cover change on regional climate. Nature Climate Change, 1(9), 472–475. 10.1038/nclimate1294

[gcb15876-bib-0063] Prenni, A. J. , Tobo, Y. , Garcia, E. , DeMott, P. J. , Huffman, J. A. , Hill, T. C. J. , McCluskey, C. S. , Prenni, J. E. , Franc, G. D. , Pöhlker, C. , Pöschl, U. , & Kreidenweis, S. M. (2013). Biological ice nuclei and the impact of rain on ice nuclei populations. AIP Conference Proceedings, 1527, 895–898. 10.1063/1.4803415

[gcb15876-bib-0064] PRISM Gridded Climate Data . (2021). PRISM climate group, Oregon State University.

[gcb15876-bib-0065] Pummer, B. G. , Bauer, H. , Bernardi, J. , Bleicher, S. , & Grothe, H. (2012). Suspendable macromolecules are responsible for ice nucleation activity of birch and conifer pollen. Atmospheric Chemistry and Physics, 12, 2541–2550. 10.5194/acp-12-2541-2012

[gcb15876-bib-0066] Seino, N. , Aoyagi, T. , & Tsuguti, H. (2018). Numerical simulation of urban impact on precipitation in Tokyo: How does urban temperature rise affect precipitation? Urban Climate, 23(March), 8–35. 10.1016/j.uclim.2016.11.007

[gcb15876-bib-0067] Shannon, F. A. (1989). The farmer’s last frontier agriculture 1860–1897. Routledge.

[gcb15876-bib-0068] Steiner, A. L. , Brooks, S. D. , Deng, C. , Thornton, D. C. O. , Pendleton, M. W. , & Bryant, V. (2015). Pollen as atmospheric cloud condensation nuclei. Geophysical Research Letters, 42(9), 3596–3602. 10.1002/2015GL064060

[gcb15876-bib-0069] Suski, K. J. , Hill, T. C. J. , Levin, E. J. T. , Miller, A. , Demott, P. J. , & Kreidenweis, S. M. (2018). Agricultural harvesting emissions of ice‐nucleating particles. Atmospheric Chemistry and Physics, 18, 13755–13771. 10.5194/acp-18-13755-2018

[gcb15876-bib-0070] Taylor, C. M. , de Jeu, R. A. M. , Guichard, F. , Harris, P. P. , & Dorigo, W. A. (2012). Afternoon rain more likely over drier soils. Nature, 489(7416), 423–426. 10.1038/nature11377 22972193

[gcb15876-bib-0071] Taylor, C. M. , Gounou, A. , Guichard, F. , Harris, P. P. , Ellis, R. J. , Couvreux, F. , & De Kauwe, M. (2011). Frequency of sahelian storm initiation enhanced over mesoscale soil‐moisture patterns. Nature Geoscience 2011, 4(7), 430–433. 10.1038/ngeo1173

[gcb15876-bib-0072] Teng, P. S. , & Close, R. C. (1978). Effect of temperature and uredinium density on urediniospore production, latent period, and infectious period of puccinia hordei otth. Journal of Agricultural Research, 21, 287–296. 10.1080/00288233.1978.10427413

[gcb15876-bib-0073] Teuling, A. J. , Seneviratne, S. I. , Stöckli, R. , Reichstein, M. , Moors, E. , Ciais, P. , Luyssaert, S. , van den Hurk, B. , Ammann, C. , Bernhofer, C. , Dellwik, E. , Gianelle, D. , Gielen, B. , Grünwald, T. , Klumpp, K. , Montagnani, L. , Moureaux, C. , Sottocornola, M. , & Wohlfahrt, G. (2010). Contrasting response of European forest and grassland energy exchange to heatwaves. Nature Geoscience, 3(10), 722–727. 10.1038/ngeo950

[gcb15876-bib-0074] Tuttle, S. , & Salvucci, G. (2016). Empirical evidence of contrasting soil moisture‐precipitation feedbacks across the United States. Science, 352(6287), 825–828. 10.1126/science.aaa7185 27174987

[gcb15876-bib-0075] U.S. Department of Agriculture, Forest Service . (2012). Future of America’s forest and rangelands: Forest service 2010 resources planning act assessment. 10.2737/WO-GTR-87

[gcb15876-bib-0076] USDA and NASS . (2012). 2012 census of agriculture. Ag Census Web Maps.

[gcb15876-bib-0077] Werth, D. , & Avissar, R. (2002). The local and global effects of Amazon deforestation. Journal of Geophysical Research, 107(D20), 8087. 10.1029/2001jd000717

[gcb15876-bib-0078] Wery, N. , Gales, A. , & Brunet, Y. (2018). Bioaerosol sources. In A.‐M. Delort & P. Amato (Eds.), Microbiology of aerosols (pp. 117–135). Wiley Blackwell.

[gcb15876-bib-0079] Wickham, H. (2016). Ggplot2: Elegant graphics for data analysis. Springer‐Verlag. https://cran.r‐project.org/web/packages/ggplot2/citation.html

[gcb15876-bib-0080] Yang, L. , Sun, G. , Zhi, L. U. , & Zhao, J. (2018). Negative soil moisture‐precipitation feedback in dry and wet regions. Scientific Reports, 8(1), 4026. 10.1038/s41598-018-22394-7 29507383 PMC5838231

[gcb15876-bib-0081] Yosef, G. , Walko, R. , Avisar, R. , Tatarinov, F. , Rotenberg, E. , & Yakir, D. (2018). Large‐scale semi‐arid afforestation can enhance precipitation and carbon sequestration potential. Scientific Reports, 8(1), 1–10. 10.1038/s41598-018-19265-6 29343760 PMC5772497

[gcb15876-bib-0082] Yu, Z. , & Lu, C. (2018). Historical cropland expansion and abandonment in the continental U.S. during 1850 to 2016. Global Ecology and Biogeography, 27(3), 322–333. 10.1111/geb.12697

[gcb15876-bib-0083] Zhang, K. E. , Kimball, J. S. , Nemani, R. R. , Running, S. W. , Hong, Y. , Gourley, J. J. , & Zhongbo, Y. U. (2015). Vegetation greening and climate change promote multidecadal rises of global land evapotranspiration. Scientific Reports, 5(October). 10.1038/srep15956 PMC4626800 26514110

[gcb15876-bib-0084] Zhou, S. , Williams, A. P. , Lintner, B. R. , Berg, A. M. , Zhang, Y. , Keenan, T. F. , Cook, B. I. , Hagemann, S. , Seneviratne, S. I. , & Gentine, P. (2021). Soil moisture–atmosphere feedbacks mitigate declining water availability in drylands. Nature Climate Change, 11(1), 38–44. 10.1038/s41558-020-00945-z

[gcb15876-bib-0085] Ziemba, L. D. , Beyersdorf, A. J. , Chen, G. , Corr, C. A. , Crumeyrolle, S. N. , Diskin, G. , Hudgins, C. , Martin, R. , Mikoviny, T. , Moore, R. , Shook, M. , Thornhill, K. L. , Winstead, E. L. , Wisthaler, A. , & Anderson, B. E. (2016). Airborne observations of bioaerosol over the southeast United States using a wideband integrated bioaerosol sensor. Journal of Geophysical Research: Atmospheres, 121(14), 8506–8524. 10.1002/2015JD024669

