# Supplementary figures and images for: Climatic and landscape changes as drivers of environmental feedback that influence rainfall frequency in the United States
Source: Glob Chang Biol. 2021 Sep 23;27(24):6381–93. doi: 10.1111/gcb.15876 (PMC9292682; doi:10.1111/gcb.15876)

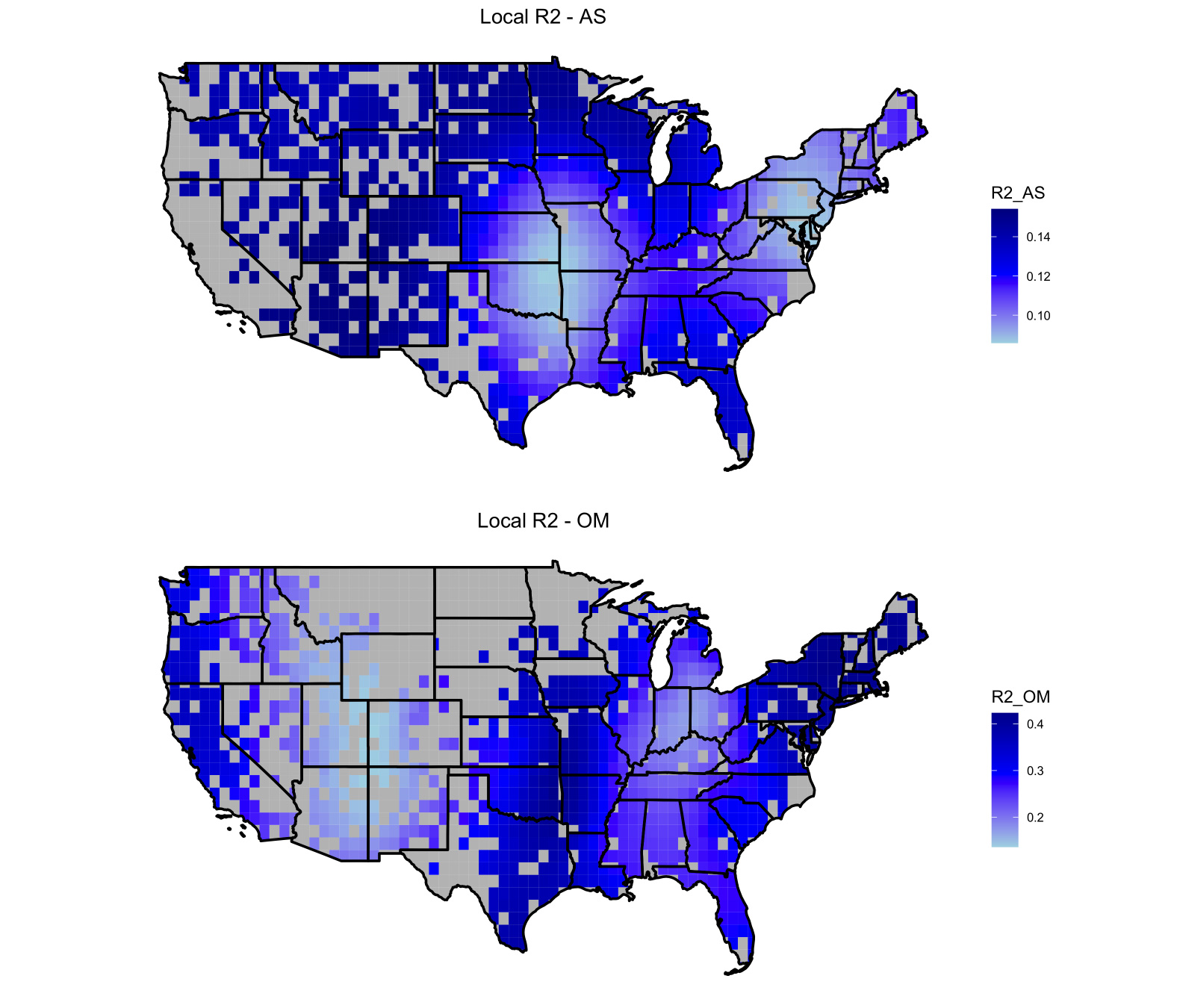

Supplement: Supplementary file 1 — Fig S1 [file GCB-27-6381-s003.jpg]

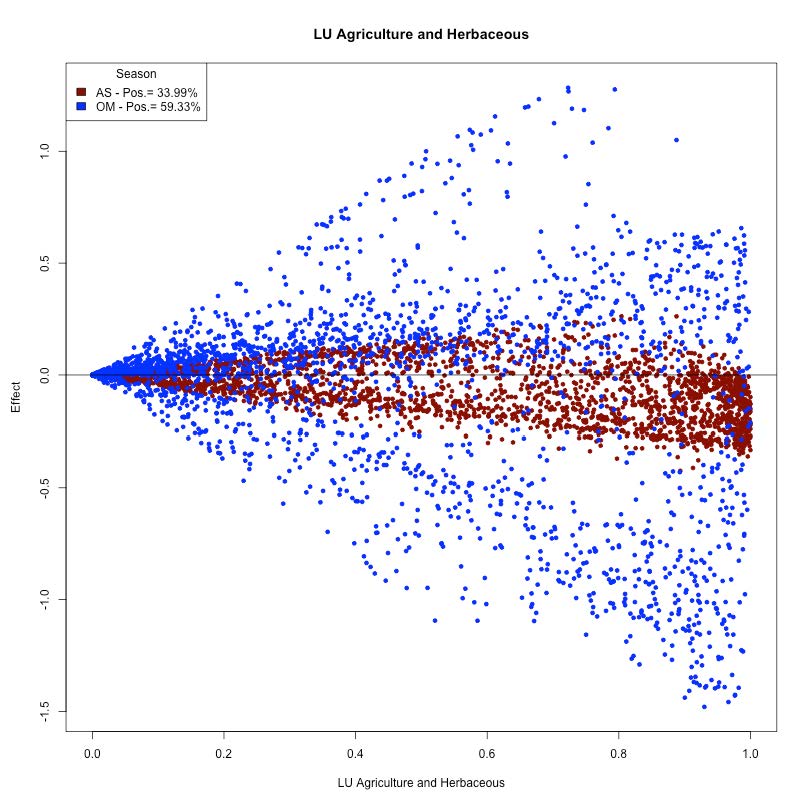

Supplement: Supplementary file 2 — Fig S2 [file GCB-27-6381-s001.jpg]

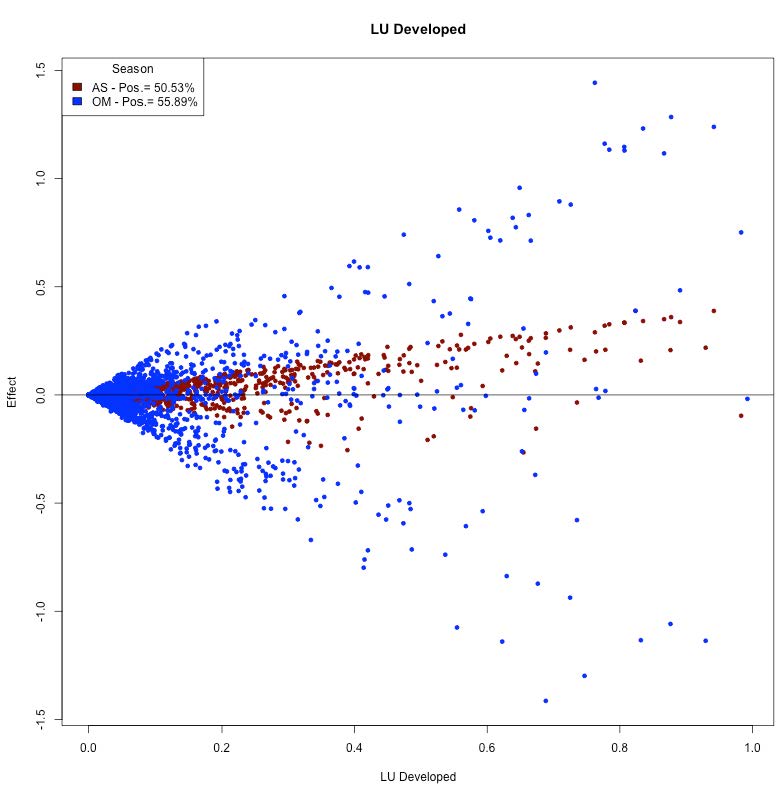

Supplement: Supplementary file 3 — Fig S3 [file GCB-27-6381-s005.jpg]
