# Supplementary material for: Climatic and landscape changes as drivers of environmental feedback that influence rainfall frequency in the United States
Source: Glob Chang Biol. 2021 Sep 23;27(24):6381–93. doi: 10.1111/gcb.15876 (PMC9292682; doi:10.1111/gcb.15876)
Supplement: Supplementary file 4 — Table S1‐6 [file GCB-27-6381-s006.docx]

Table S1. R^2^, global R^2^ with standard deviation (SD), root mean square error, Moran’s *I*, and the adaptive bandwidth selected for each summer, winter, and entire period (EP) model of rainfall feedback (Ordinary least squares: OLS; geographically weighted regression: GWR).

|  | R^2^ and Local R^2^ | | RMSE | | Moran’s *I* residuals  (p-value) | | Adaptive bandwidth (number of neighboring sites) |
| --- | --- | --- | --- | --- | --- | --- | --- |
|  | OLS | GWR | OLS | GWR | OLS | GWR |  |
| Summer | 0.08 | 0.14 | 0.02 | 0.02 | 0.16  (<1×10^-11^) | 0.12  (0.01) | 455 of 2751 |
| Winter | 0.15 | 0.38 | 0.03 | 0.02 | 0.31  (<2×10^-16^) | 0.12  (0.01) | 119 of 2299 |
| EP | 0.15 | 0.40 | 0.01 | 0.01 | 0.27  (<2×10^-16^) | 0.07  (0.1) | 114 of 2889 |

Table S2. Rainfall feedback model data and sources

| Data | Dates | Source |
| --- | --- | --- |
| Rainfall feedback | 1849-2016 | Rainfall feedback maps  <http://w3.avignon.inra.fr/rainfallfeedback/> |
| Land use | 1970 | U.S. Geological Survey  <https://www.usgs.gov/core-science-systems/eros/lulc/data-tools> |
| Land use | 2011 | Multi-Resolution Land Characteristics Consortium  <https://www.mrlc.gov/data/nlcd-2011-land-cover-conus-0> |
| Weather  “before” | 1950 | PRISM Climate Group  <http://prism.oregonstate.edu> |
| Weather  “after” | 2000 | WorldClim Version2  <http://worldclim.org/version2> |

Table S3. Summary statistics of rainfall feedback and predictor variables.

| Category | Variable | Min. | 1% Quant. | Median | 99% Quant. | Max. |
| --- | --- | --- | --- | --- | --- | --- |
| Dependent variable: RF between 1960 and 2016 | April – Sept. | -0.1160 | -0.0396 | 0.0056 | 0.0550 | 0.2408 |
|  | Oct.- March | -0.1189 | -0.0642 | -0.0011 | 0.0758 | 0.14 |
| Past rainfall feedback  (before 1960) | RF April – Sept. | -0.2347 | -0.0657 | 0.0043 | 0.0809 | 0.2158 |
| Past rainfall feedback  (before 1960) | Spatially weighted RF April – Sept. | -0.034 | -0.0205 | 0.0048 | 0.0316 | 0.0428 |
|  | RF Oct. - March | -0.241 | -0.0878 | 0.0008 | 0.0929 | 0.2043 |
|  | Spatially weighted RF  Oct. - March | -0.055 | -0.0403 | 0.0004 | 0.4409 | 0.0568 |
| Landscape | Altitude | -57 | 2 | 291 | 2420 | 3424 |
| Land use before 1960 (%) | Water | 0.00 | 0.00 | 0.25 | 28.66 | 85.13 |
|  | Ice | 0.00 | 0.00 | 0.00 | 0.78 | 49.54 |
|  | Developed | 0.00 | 0.00 | 2.68 | 67.53 | 99.23 |
|  | Barren | 0.00 | 0.00 | 0.21 | 8.39 | 37.53 |
|  | Forest | 0.00 | 0.00 | 7.79 | 90.27 | 98.53 |
|  | Evergreen | 0.00 | 0.00 | 0.30 | 91.58 | 100.00 |
|  | Shrubs | 000 | 0.00 | 0.00 | 93.71 | 99.96 |
|  | Agriculture & Herbaceous | 0.00 | 0.00 | 46.56 | 99.66 | 100.00 |
|  | Wetland | 0.00 | 0.00 | 0.00 | 14.66 | 69.90 |
| Land use change between 1960 and 2016 (∆%) | ∆ Water | -20.20 | -2.03 | +0.25 | +5.77 | +53.81 |
|  | ∆ Ice | -44.76 | -0.77 | +0.00 | +0.00 | +0.04 |
|  | ∆ Developed | -34.69 | -3.41 | +4.38 | +28.82 | +74.59 |
|  | ∆ Barren | -29.53 | -6.81 | -0.05 | +6.74 | +45.31 |
|  | ∆ Forest | -80.24 | -38.92 | +0.97 | +29.05 | +49.73 |
|  | ∆ Evergreen | -65.45 | -43.27 | +0.01 | +24.81 | +53.96 |
|  | ∆ Shrub | -91.47 | -46.44 | +0.38 | +33.21 | +78.66 |
|  | ∆ Agriculture | -74.82 | -38.19 | -6.81 | +41.18 | +90.64 |
|  | ∆ Wetland | -25.93 | -4.45 | +0.10 | +6.74 | +24.91 |
| Climate  April – Sept. | Min. Temp (°C) | -3.48 | 0.99 | 10.54 | 22.01 | 24.74 |
|  | Mean Temp (°C) | 5.05 | 9.19 | 19.03 | 27.38 | 29.38 |
|  | Max. Temp (°C) | 9.74 | 14.61 | 23.82 | 30.25 | 34.34 |
|  | Min. Prec. (mm) | 0.00 | 2.74 | 63.19 | 106.99 | 140.40 |
|  | Mean Prec. (mm) | 1.79 | 12.87 | 90.69 | 154.08 | 177.68 |
|  | Max. Prec. (mm) | 4.48 | 20.71 | 112.61 | 213.34 | 257.37 |
| Climate  Oct. – March | Min. Temp (°C) | -13.35 | -10.58 | 1.01 | 16.51 | 20.87 |
|  | Mean Temp (°C) | -7.30 | -4.30 | 5.22 | 19.07 | 22.80 |
|  | Max. Temp (°C) | 0.99 | 4.70 | 13.98 | 24.16 | 26.39 |
|  | Min. Prec. (mm) | 1.82 | 5.43 | 51.74 | 140.32 | 321.62 |
|  | Mean Prec. (mm) | 5.07 | 10.89 | 72.80 | 229.34 | 397.26 |
|  | Max. Prec. (mm) | 9.07 | 17.17 | 92.38 | 332.25 | 513.59 |
| Change in Climate April - Sept. | Min. Temp (°C) | -3.01 | -1.74 | -0.76 | +0.41 | +1.51 |
|  | Mean Temp (°C) | -2.00 | -1.15 | -0.31 | +1.03 | +5.92 |
|  | Max. Temp (°C) | -1.47 | -0.72 | +0.11 | +1.87 | +4.63 |
|  | Min. Prec. (mm) | -95.59 | -52.85 | -13.47 | +6.07 | +28.84 |
|  | Mean Prec. (mm) | -41.24 | -13.97 | 2.91 | +17.09 | +42.17 |
|  | Max. Prec. (mm) | -8.43 | +3.06 | +20.01 | +51.10 | +81.53 |
| Change in Climate Oct. – March | Min. Temp (°C) | -3.63 | -2.91 | -1.66 | -0.07 | +1.15 |
|  | Mean Temp (°C) | -1.97 | -1.16 | -0.33 | +0.88 | +1.70 |
|  | Max. Temp (°C) | -1.06 | -0.11 | +1.04 | +2.61 | +3.33 |
|  | Min. Prec. (mm) | -307.2 | -91.22 | -12.55 | +11.89 | +33.21 |
|  | Mean Prec. (mm) | -123.9 | -37.32 | +3.85 | +27.66 | +52.96 |
|  | Max. Prec. (mm) | -74.56 | -8.73 | +19.83 | +59.44 | +81.61 |

Table S4. GWR model summary statistics for climate and climate change effects on RF for summer and winter models. Summary statistics depict minimum, first quartile, median, mean, third quartile, and maximum values of predictor variable effects (product of the coefficient and value) across the study area.

|  | Independent variables |  | Effect summary statistics | | | | | |  | % locations with positive/negative effects  (% significant coefficients) | |
| --- | --- | --- | --- | --- | --- | --- | --- | --- | --- | --- | --- |
|  |  | Min. | | 1^st^ Q. | Med. | Mean | 3rd Q. | Max. | Pos. | | Neg. |
| Summer | Max. temp. | -0.96 | | -0.39 | -0.24 | -0.24 | -0.08 | 0.16 | 17 (0) | | 83 (44) |
|  | Max. prec. | -0.50 | | -0.26 | -0.13 | -0.17 | -0.07 | -0.01 | 0 (0) | | 100 (67) |
|  | Min. ∆temp. | -0.10 | | -0.02 | 0.00 | -0.01 | 0.01 | 0.08 | 43 (3) | | 57 (0) |
|  | Max. ∆temp. | -0.12 | | -0.01 | 0.00 | 0.00 | 0.00 | 0.09 | 47 (9) | | 53 (0) |
|  | Mean ∆prec. | -0.16 | | -0.01 | 0.00 | 0.01 | 0.02 | 0.16 | 60 (29) | | 40 (0) |
|  | Min ∆prec. | -0.12 | | 0.02 | 0.05 | 0.06 | 0.08 | 0.35 | 93 (0) | | 7 (89) |
|  | Max ∆prec. | -0.29 | | -0.09 | -0.06 | -0.07 | -0.03 | 0.05 | 0 (0) | | 100 (74) |
| Winter | Max. prec. | -2.00 | | -0.20 | 0.10 | -0.02 | 0.29 | 1.05 | 61 (36) | | 39 (21) |
|  | Min. ∆temp. | -1.12 | | -0.43 | -0.24 | -0.23 | -0.06 | 0.84 | 17 (39) | | 83 (4) |
|  | Max. ∆temp. | -1.59 | | -0.12 | 0.05 | -0.01 | 0.17 | 0.90 | 61 (17) | | 39 (18) |
|  | Mean ∆prec. | -0.84 | | 0.00 | 0.02 | 0.03 | 0.06 | 0.67 | 70 (7) | | 30 (0) |
|  | Min. ∆prec. | -0.45 | | -0.02 | 0.02 | 0.04 | 0.09 | 0.82 | 65 (11) | | 35 (18) |
|  | Max. ∆prec. | -0.61 | | -0.10 | -0.03 | -0.01 | 0.06 | 0.53 | 40 (17) | | 60 (14) |

Table S5. GWR model summary statistics for land use effects on RF for summer and winter models.

|  | Independent variables |  | Effect summary statistics | | | | |  | % locations with positive/negative or zero effect  (% significant) | | | |
| --- | --- | --- | --- | --- | --- | --- | --- | --- | --- | --- | --- | --- |
|  |  | Min. | 1^st^ Q. | Med. | Mean | 3rd Q. | Max | Pos. | | 0 | Neg. |  |
| Summer | Developed | -0.27 | 0.00 | 0.00 | 0.01 | 0.01 | 0.39 | 51 (12) | | 3 | 49 (9) |  |
|  | Barren | -0.45 | 0.00 | 0.00 | 0.01 | 0.01 | 1.32 | 50 (34) | | 22 | 50 (21) |  |
|  | Forest | -0.12 | 0.00 | 0.00 | 0.05 | 0.07 | 0.46 | 54 (21) | | 21 | 46 (0) |  |
|  | Evergreen | -0.56 | 0.00 | 0.00 | -0.02 | 0.00 | 0.34 | 31 (6) | | 45 | 69 (28) |  |
|  | Shrubs | -0.61 | 0.00 | 0.00 | -0.04 | 0.00 | 0.01 | 3 (0) | | 68 | 97 (20) |  |
|  | Ag and herb | -0.42 | -0.13 | -0.03 | -0.06 | 0.01 | 0.26 | 34 (1) | | 2 | 66 (9) |  |
|  | Wetland | -0.24 | 0.00 | 0.00 | 0.00 | 0.00 | 0.42 | 17 (17) | | 68 | 83 (0) |  |
| Winter | Developed | -1.41 | -0.02 | 0.00 | 0.00 | 0.02 | 1.44 | 56 (12) | | 2 | 44 (21) |  |
|  | Barren | -0.17 | 0.00 | 0.00 | 0.03 | 0.02 | 1.67 | 66 (13) | | 18 | 34 (0) |  |
|  | Forest | -1.71 | -0.07 | 0.00 | -0.01 | 0.02 | 1.99 | 40 (10) | | 17 | 60 (20) |  |
|  | Evergreen | -0.91 | 0.00 | 0.00 | 0.00 | 0.00 | 0.75 | 30 (0) | | 36 | 70 (10) |  |
|  | Shrubs | -0.17 | 0.00 | 0.00 | 0.05 | 0.01 | 1.85 | 30 (18) | | 64 | 70 (4) |  |
|  | Ag and herb | -1.48 | -0.22 | 0.01 | -0.07 | 0.15 | 1.28 | 59 (12) | | 3 | 41 (17) |  |
|  | Wetland | -0.30 | 0.00 | 0.00 | 0.00 | 0.00 | 0.66 | 15 (2) | | 71 | 85 (1) |  |

Table S6. GWR model summary statistics for land use change effects on RF for summer and winter models.

|  | Independent variables |  | Effect summary statistics | | | | | | |  | | % locations with positive/negative or zero effect  (% significant) | | | |
| --- | --- | --- | --- | --- | --- | --- | --- | --- | --- | --- | --- | --- | --- | --- | --- |
|  |  | Min. | | 1^st^ Q. | Med. | Mean | 3rd Q. | | Max. | | Pos. | | 0 | Neg. |  |
| Summer | ∆Developed | -1.22 | | -0.05 | -0.02 | -0.03 | | 0.00 | 0.56 | | 31 (0) | | 1 | 69 (34) |  |
|  | ∆Barren | -1.13 | | -0.01 | 0.00 | -0.01 | | 0.00 | 0.38 | | 39 (28) | | 3 | 61 (25) |  |
|  | ∆Forest | -0.65 | | -0.02 | 0.00 | 0.01 | | 0.02 | 0.77 | | 46 (0) | | 1 | 54 (22) |  |
|  | ∆Evergreen | -0.52 | | 0.00 | 0.00 | 0.01 | | 0.01 | 0.75 | | 47 (0) | | 12 | 53 (23) |  |
|  | ∆Shrubland | -1.06 | | -0.01 | 0.00 | 0.00 | | 0.01 | 1.09 | | 47 (4) | | 10 | 53 (33) |  |
|  | ∆Agriculture | -0.96 | | -0.03 | 0.00 | 0.01 | | 0.06 | 0.93 | | 52 (0) | | 0 | 48 (35) |  |
|  | ∆Wetland | -0.29 | | 0.00 | 0.00 | 0.00 | | 0.00 | 0.22 | | 42 (24) | | 9 | 58 (32) |  |
| Winter | ∆Developed | -1.28 | | -0.02 | 0.00 | 0.00 | | 0.03 | 0.99 | | 47 (0) | | 0 | 53 (10) |  |
|  | ∆Barren | -1.41 | | -0.01 | 0.00 | -0.01 | | 0.00 | 0.50 | | 43 (9) | | 2 | 57 (0.5) |  |
|  | ∆Forest | -1.01 | | -0.02 | 0.00 | 0.01 | | 0.03 | 1.31 | | 54 (2) | | 1 | 46 (3) |  |
|  | ∆Evergreen | -0.96 | | -0.01 | 0.00 | 0.00 | | 0.01 | 0.86 | | 45 (0) | | 7 | 55 (3) |  |
|  | ∆Shrubland | -1.58 | | 0.00 | 0.00 | 0.02 | | 0.04 | 0.91 | | 63 (14) | | 7 | 37 (3) |  |
|  | ∆Agriculture | -0.97 | | -0.05 | 0.00 | 0.00 | | 0.04 | 1.44 | | 46 (0) | | 0 | 54 (3) |  |
|  | ∆Wetland | -0.82 | | 0.00 | 0.00 | 0.00 | | 0.00 | 0.31 | | 43 (0) | | 11 | 57 (8) |  |
