# Supplementary material for: Climatic and landscape changes as drivers of environmental feedback that influence rainfall frequency in the United States
Source: Glob Chang Biol. 2021 Sep 23;27(24):6381–93. doi: 10.1111/gcb.15876 (PMC9292682; doi:10.1111/gcb.15876)
Supplement: Supplementary file 5 — Supplementary Material [file GCB-27-6381-s004.pdf]

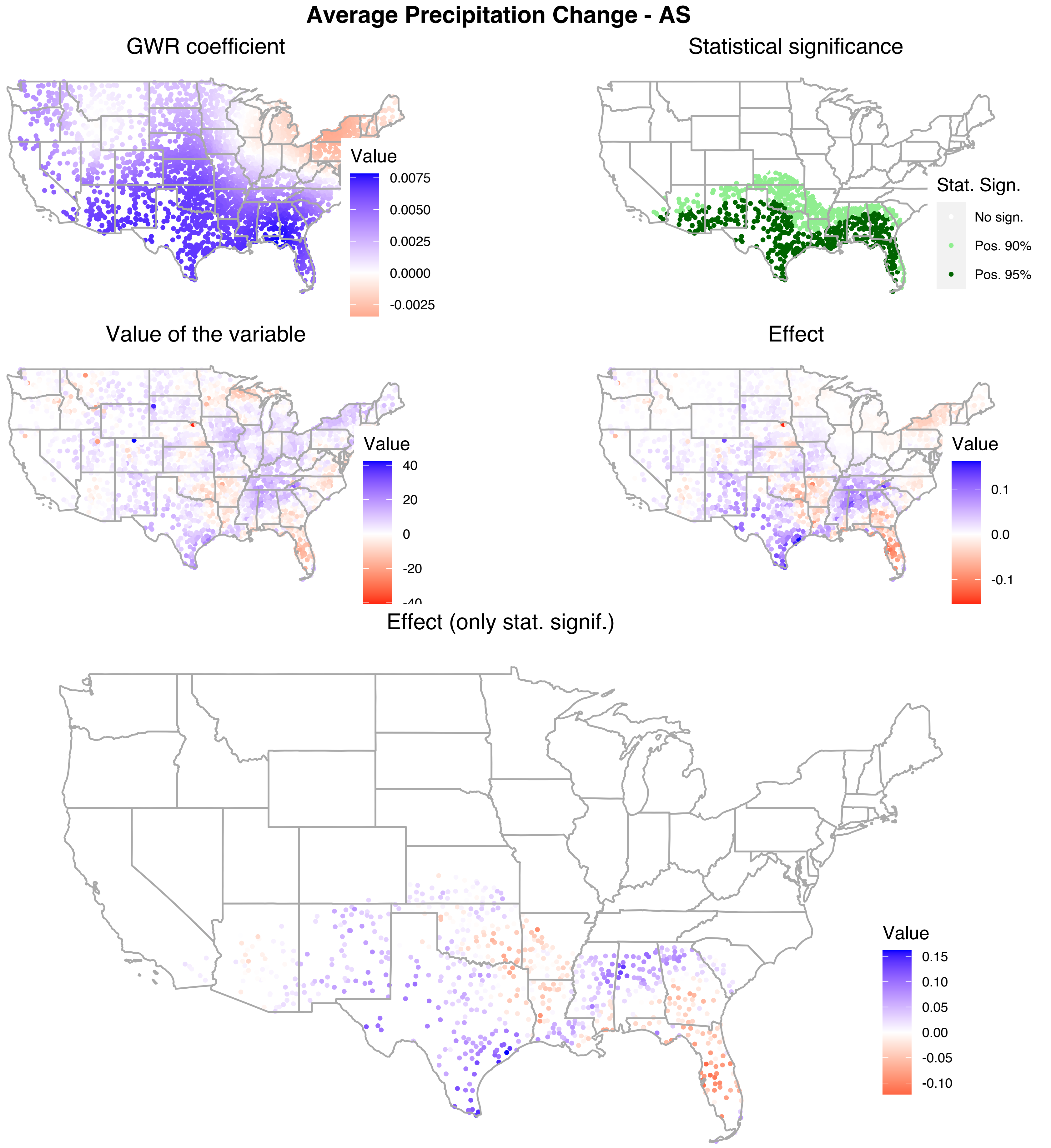
Average Precipitation Change - AS
GWR coefficient
Value
0.0075
0.0050
0.0025
0.0000
-0.0025
Statistical significance
Stat. Sign.
No sign.
Pos. 90%
Pos. 95%
Value of the variable
Value
40
20
0
-20
Effect
Value
0.1
0.0
-0.1
Effect (only stat. signif.)
Value
0.15
0.10
0.05
0.00
-0.05
-0.10

Change LU Agriculture and Herbaceous - AS

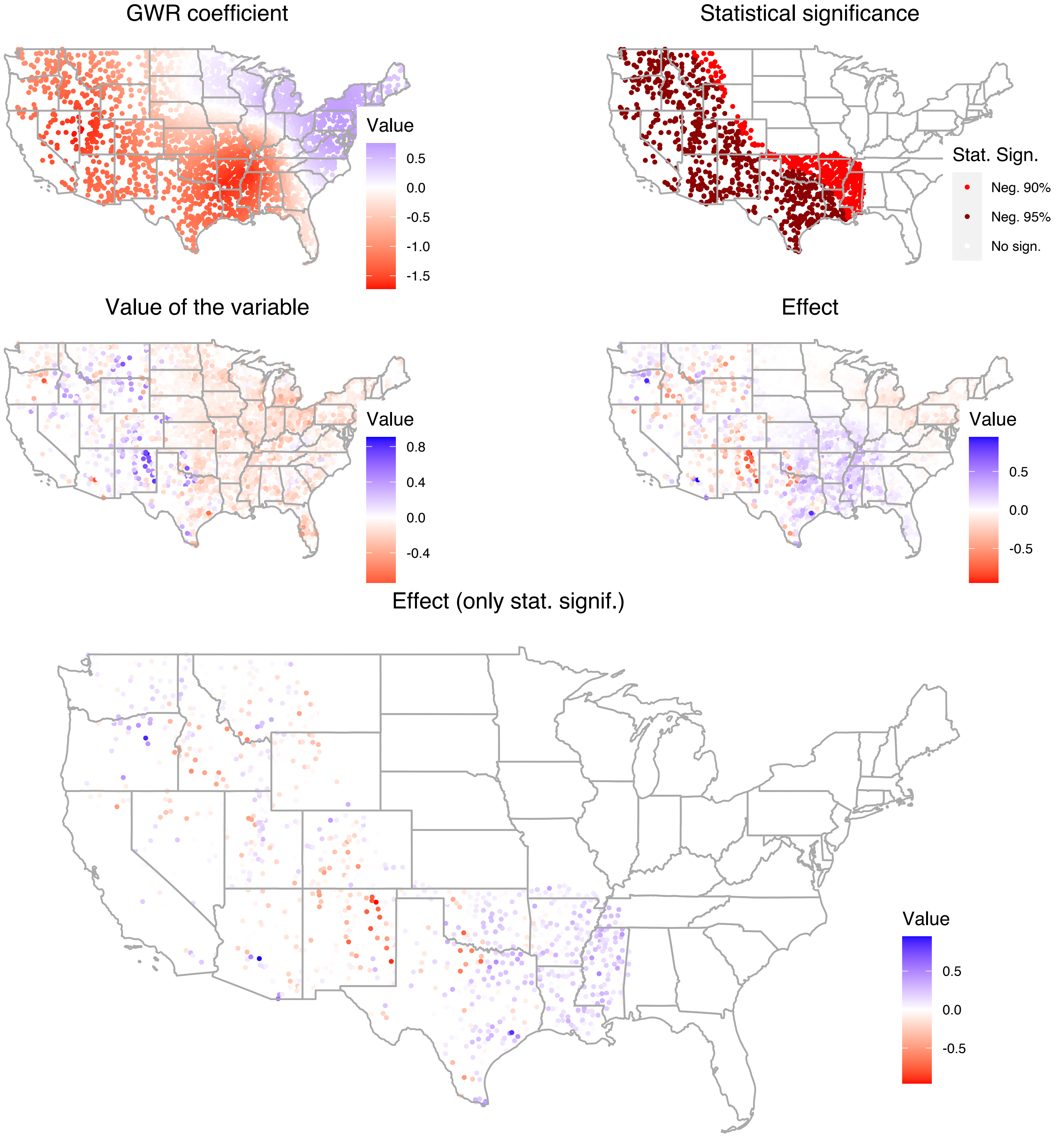

Change LU Barren - AS

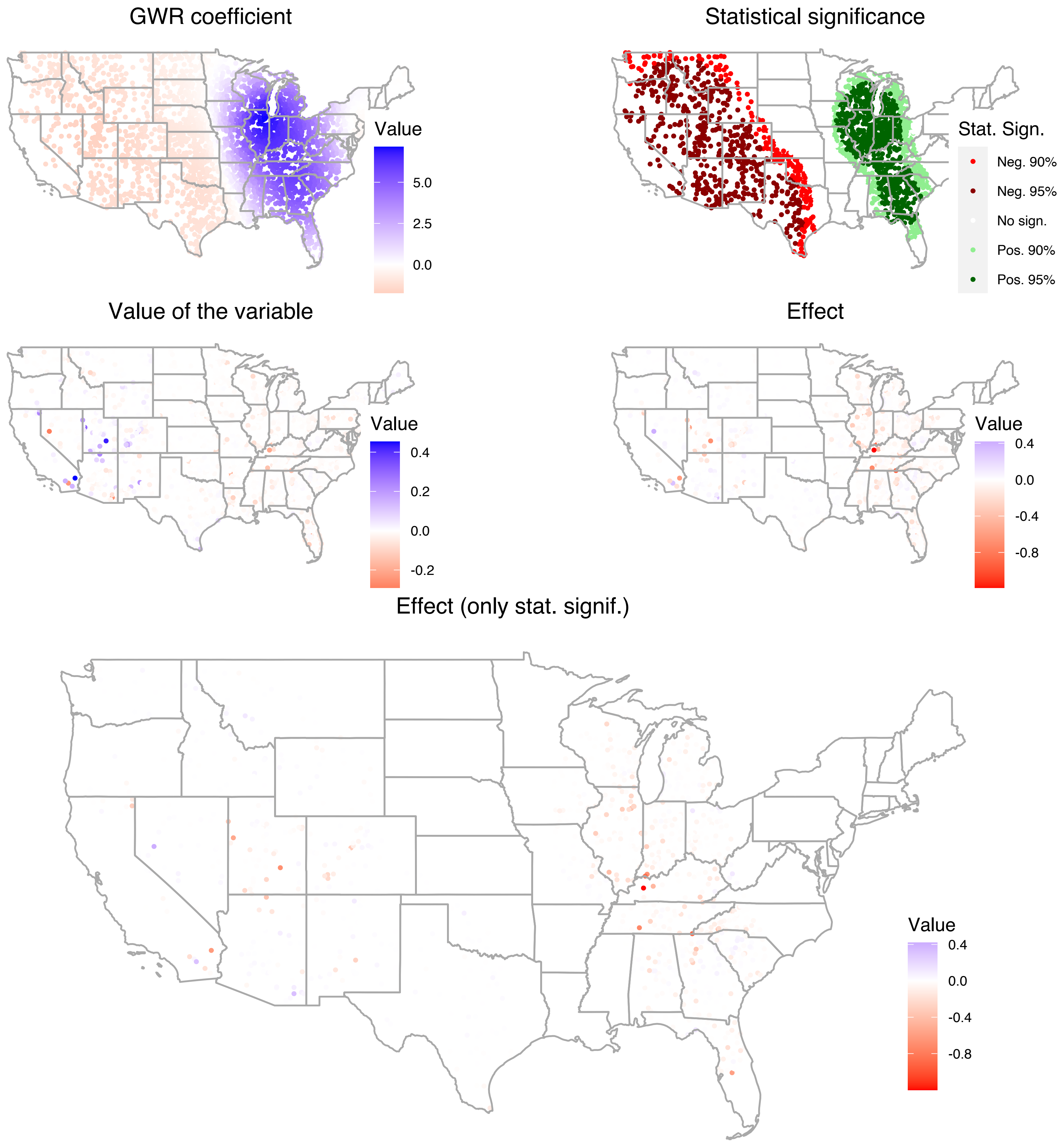

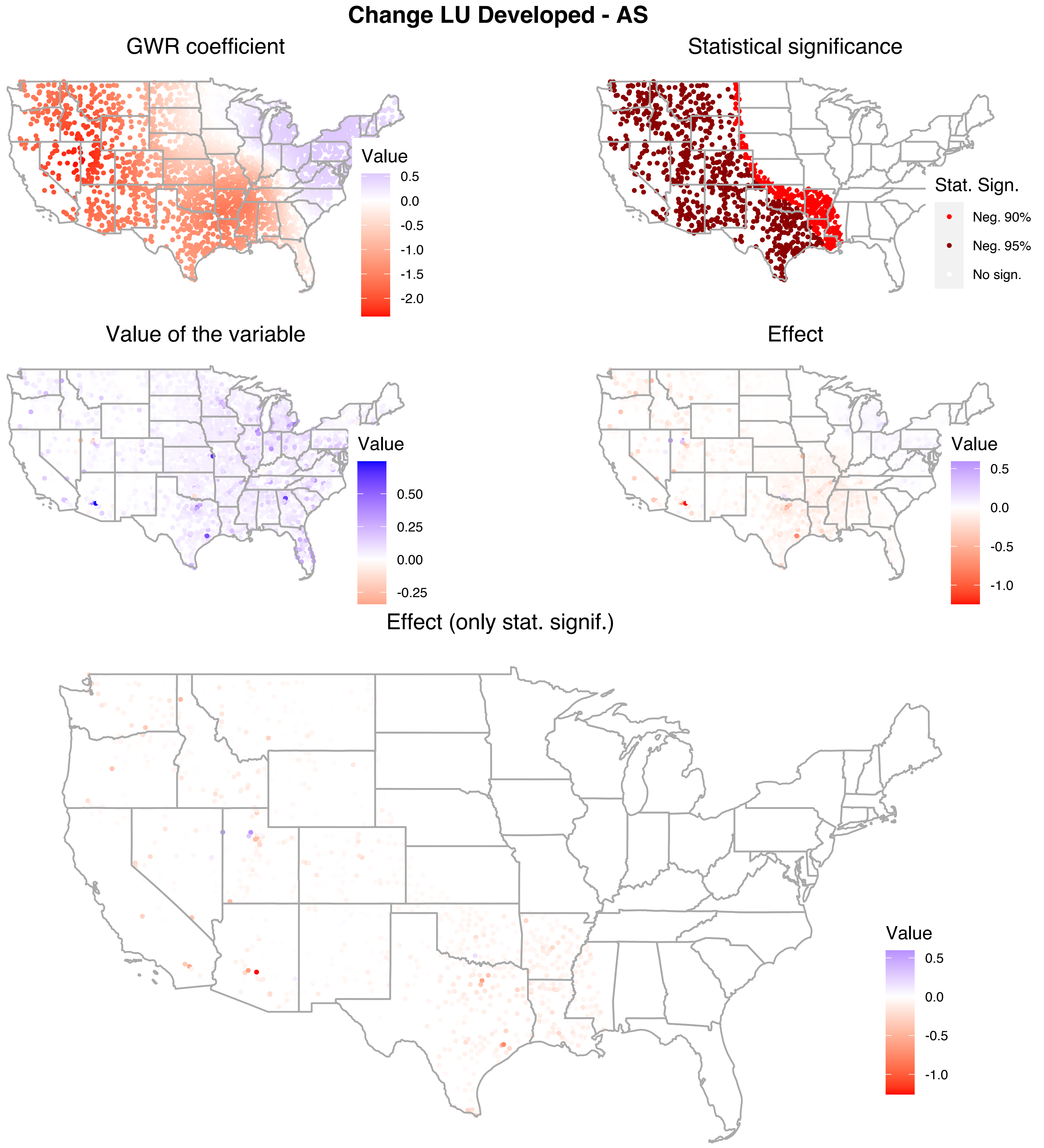
Change LU Developed - AS
GWR coefficient
Value
0.5
0.0
-0.5
-1.0
-1.5
-2.0
Statistical significance
Stat. Sign.
Neg. 90%
Neg. 95%
No sign.
Value of the variable
Value
0.50
0.25
0.00
-0.25
Effect
Value
0.5
0.0
-0.5
-1.0
Effect (only stat. signif.)
Value
0.5
0.0
-0.5
-1.0

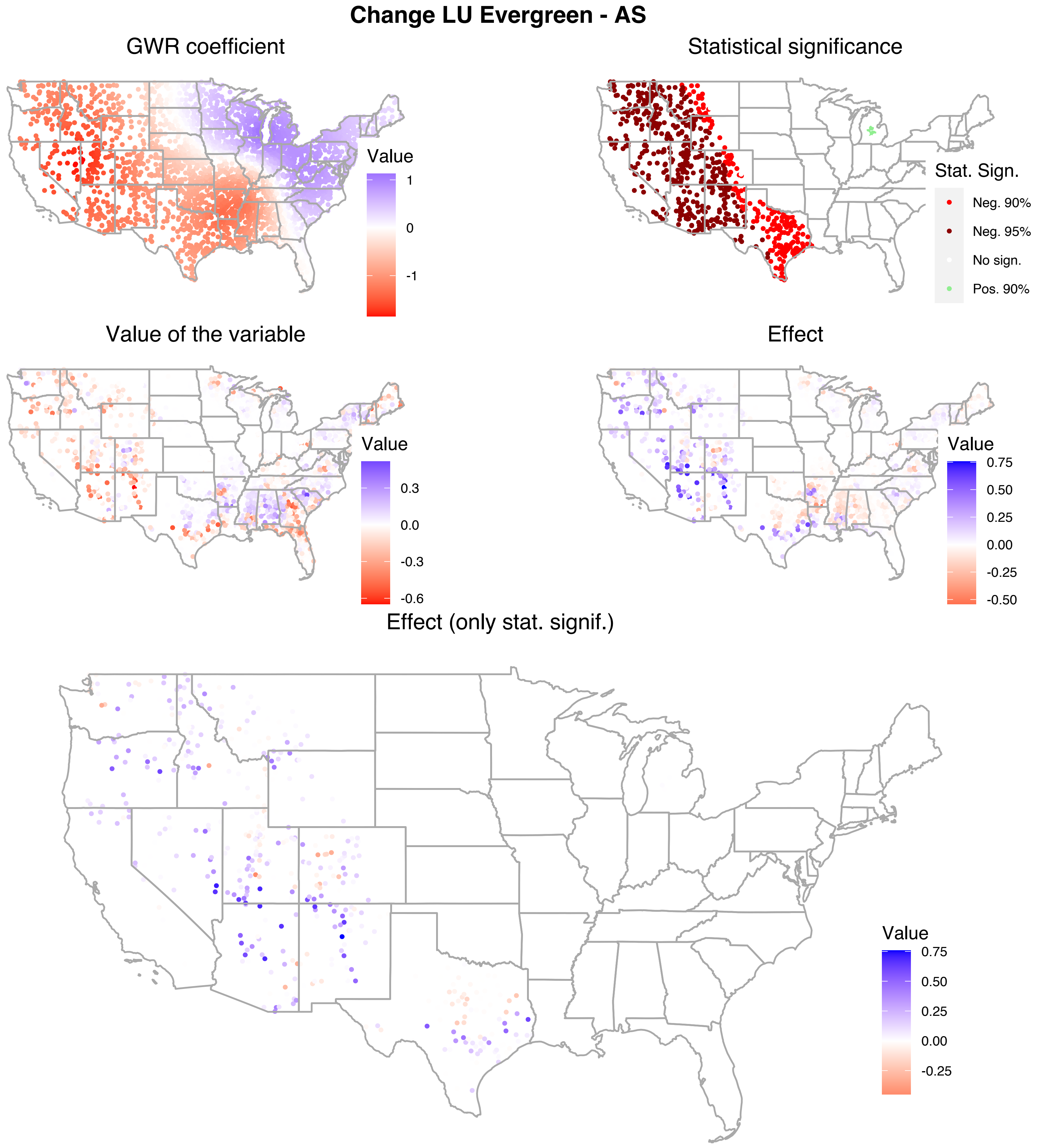
Change LU Evergreen - AS
GWR coefficient
Value
1
0
-1
Statistical significance
Stat. Sign.
Neg. 90%
Neg. 95%
No sign.
Pos. 90%
Value of the variable
Value
0.3
0.0
-0.3
-0.6
Effect
Value
0.75
0.50
0.25
0.00
-0.25
-0.50
Effect (only stat. signif.)
Value
0.75
0.50
0.25
0.00
-0.25

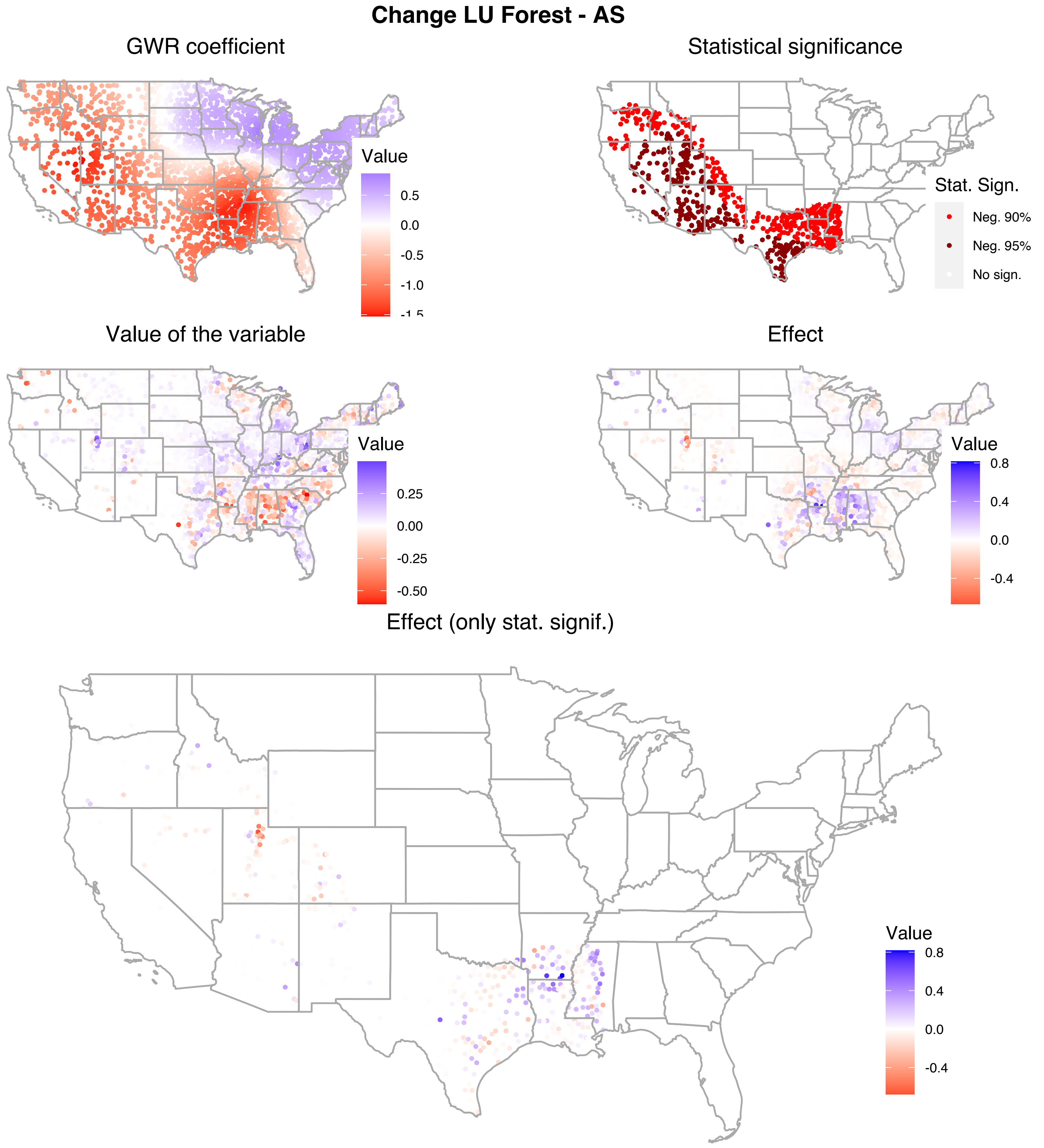
Change LU Forest - AS
GWR coefficient
Value
0.5
0.0
-0.5
-1.0
Statistical significance
Stat. Sign.
Neg. 90%
Neg. 95%
No sign.
Value of the variable
Value
0.25
0.00
-0.25
-0.50
Effect
Value
0.8
0.4
0.0
-0.4
Effect (only stat. signif.)
Value
0.8
0.4
0.0
-0.4

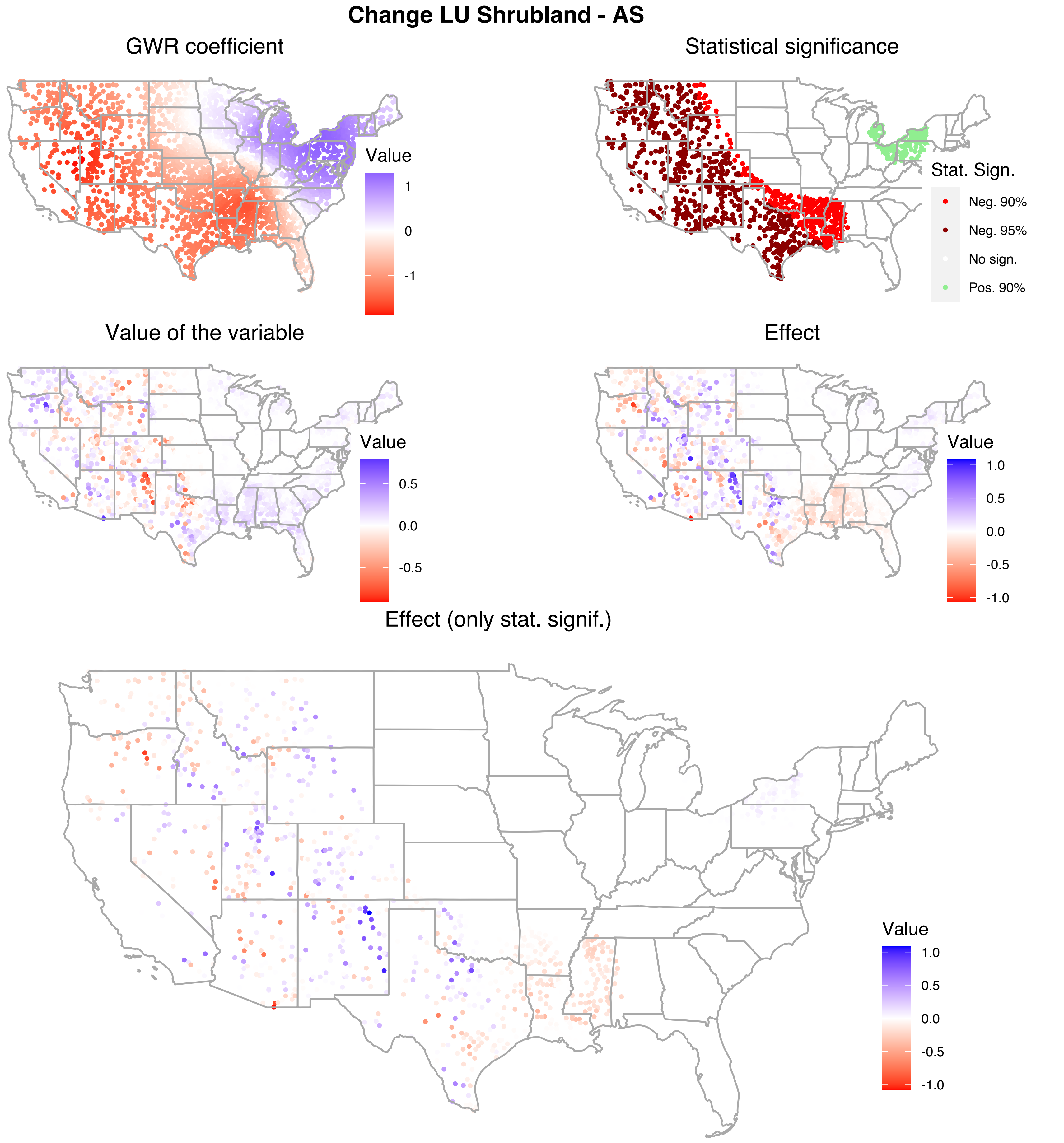

Change LU Shrubland - AS
GWR coefficient
Value
1
0
-1
Statistical significance
Stat. Sign.
Neg. 90%
Neg. 95%
No sign.
Pos. 90%
Value of the variable
Value
0.5
0.0
-0.5
Effect
Value
1.0
0.5
0.0
-0.5
-1.0
Effect (only stat. signif.)
Value
1.0
0.5
0.0
-0.5
-1.0

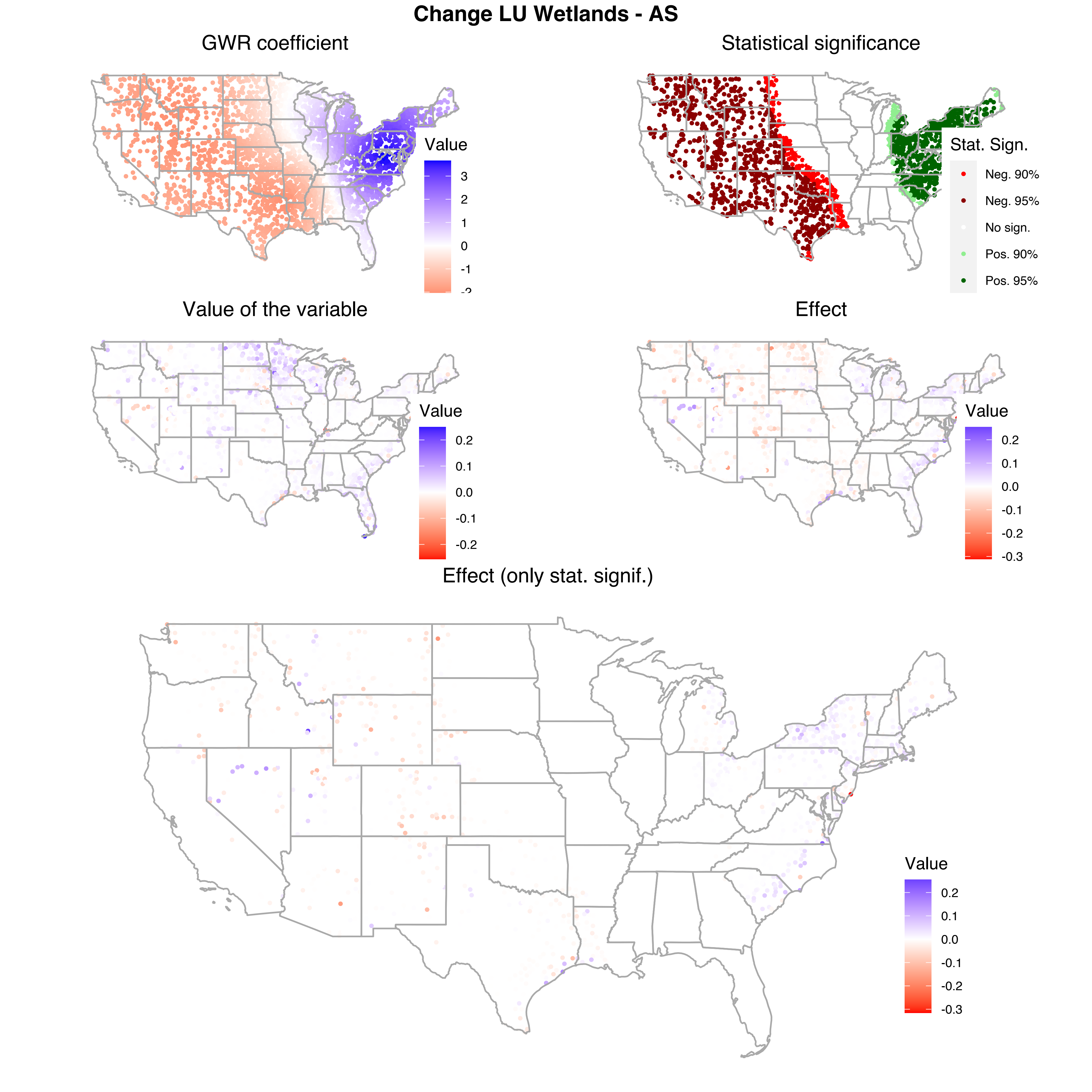

Change LU Wetlands - AS
GWR coefficient
Value
3
2
1
0
-1
Statistical significance
Stat. Sign.
Neg. 90%
Neg. 95%
No sign.
Pos. 90%
Pos. 95%
Value of the variable
Value
0.2
0.1
0.0
-0.1
-0.2
Effect
Value
0.2
0.1
0.0
-0.1
-0.2
-0.3
Effect (only stat. signif.)
Value
0.2
0.1
0.0
-0.1
-0.2
-0.3

LU Agriculture and Herbaceous - AS

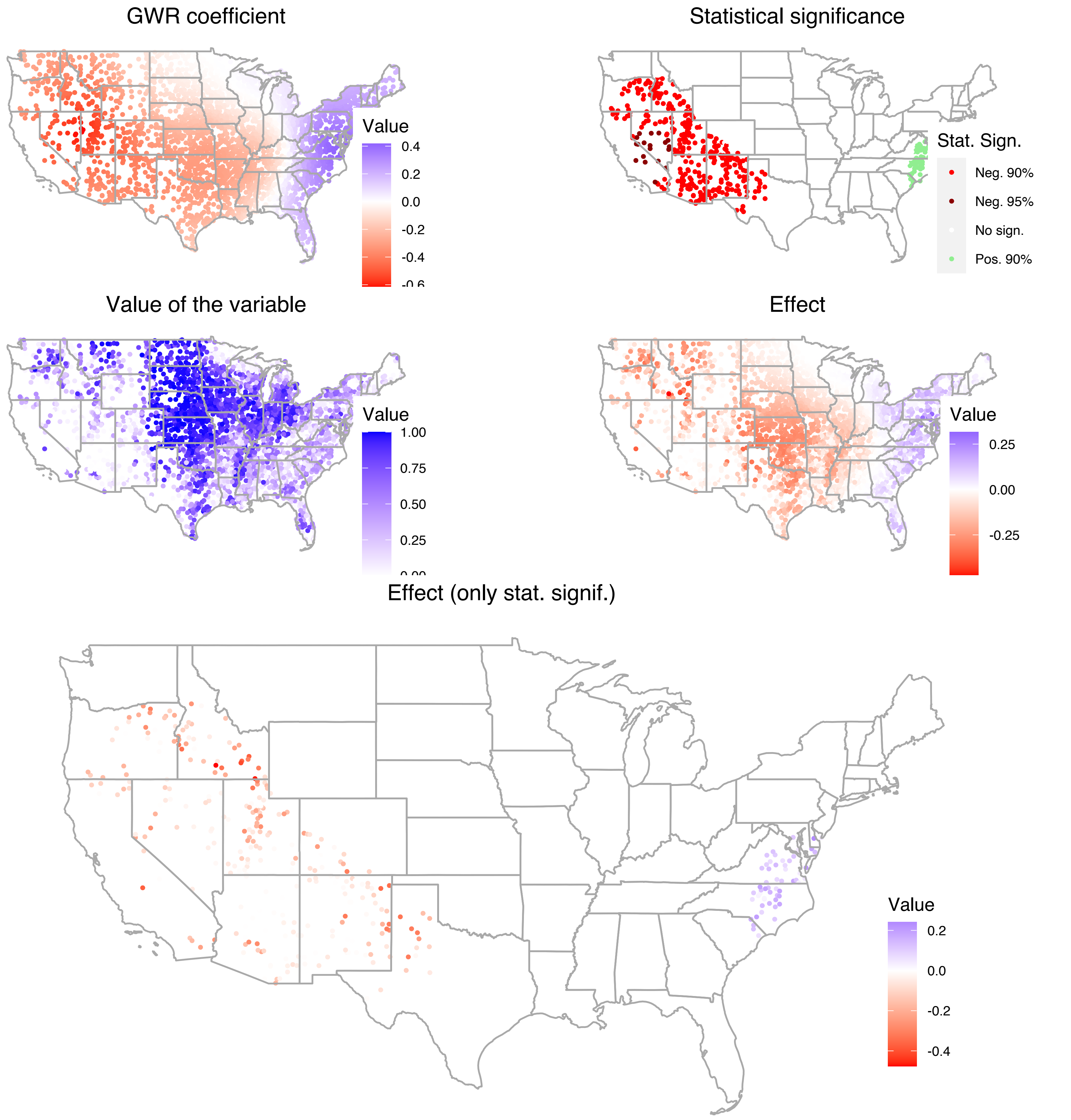

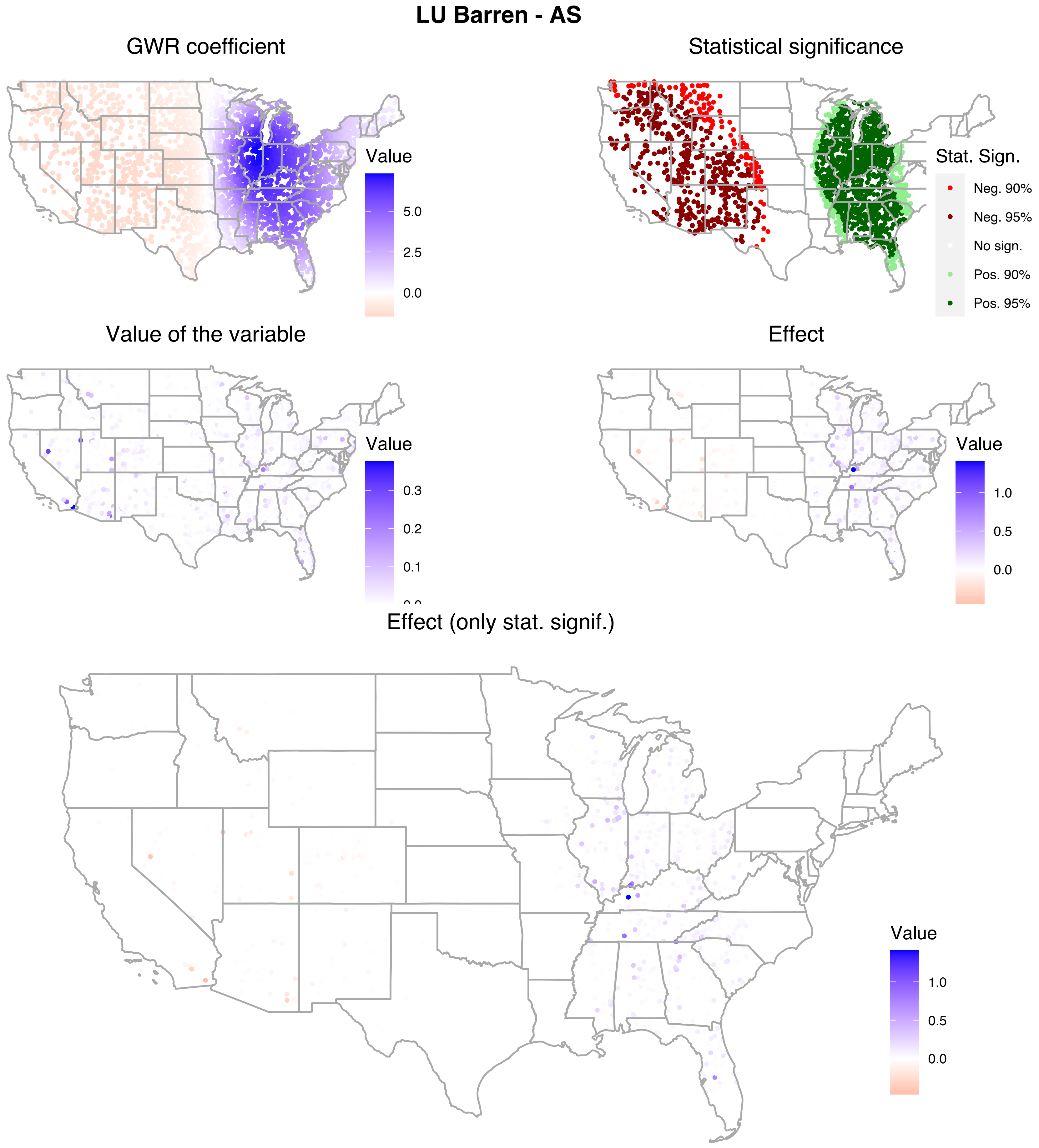
LU Barren - AS
GWR coefficient
Value
5.0
2.5
0.0
Statistical significance
Stat. Sign.
Neg. 90%
Neg. 95%
No sign.
Pos. 90%
Pos. 95%
Value of the variable
Value
0.3
0.2
0.1
Effect
Value
1.0
0.5
0.0
Effect (only stat. signif.)
Value
1.0
0.5
0.0

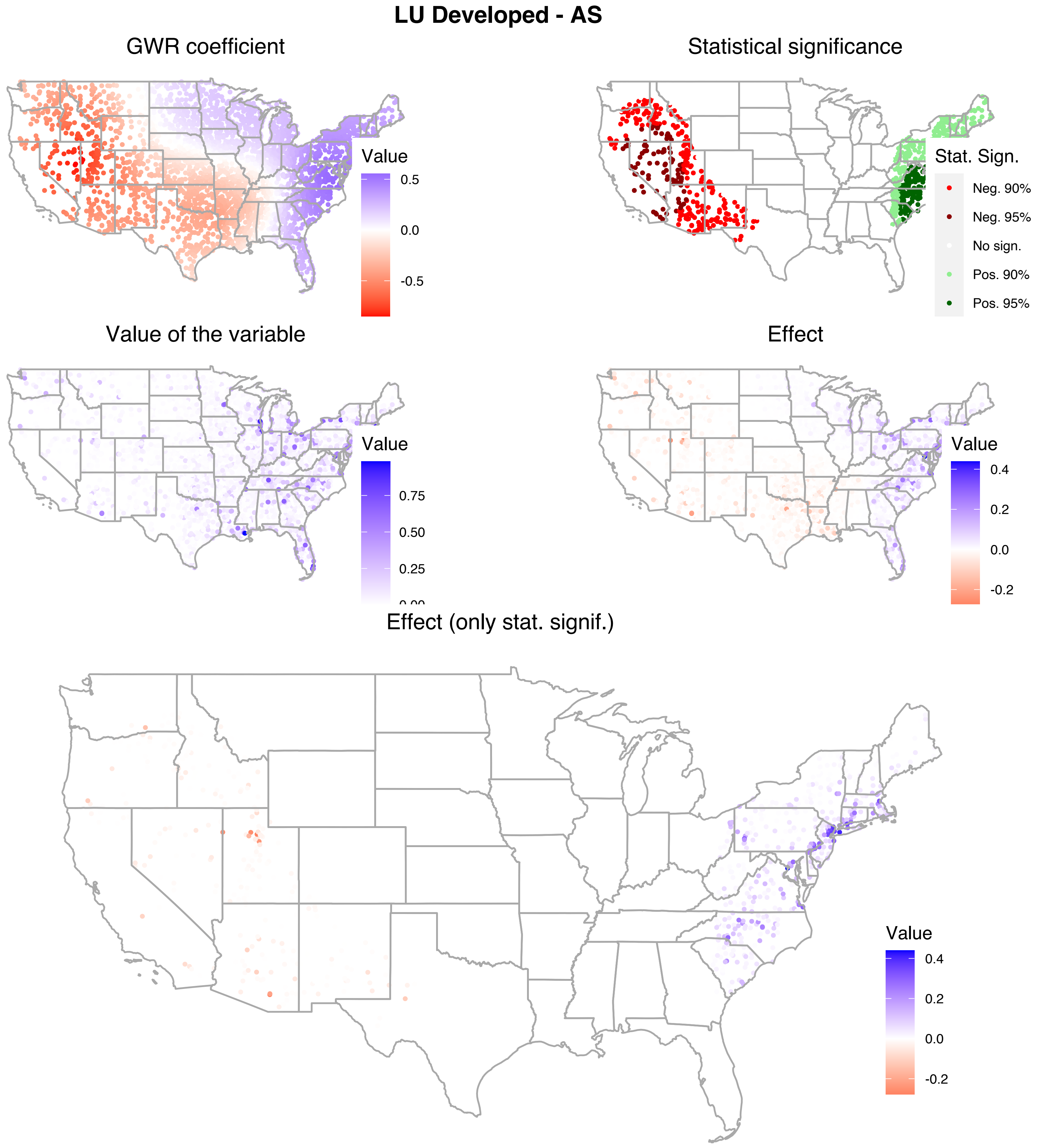

LU Developed - AS
GWR coefficient
Value
0.5
0.0
-0.5
Statistical significance
Stat. Sign.
Neg. 90%
Neg. 95%
No sign.
Pos. 90%
Pos. 95%
Value of the variable
Value
0.75
0.50
0.25
Effect
Value
0.4
0.2
0.0
-0.2
Effect (only stat. signif.)
Value
0.4
0.2
0.0
-0.2

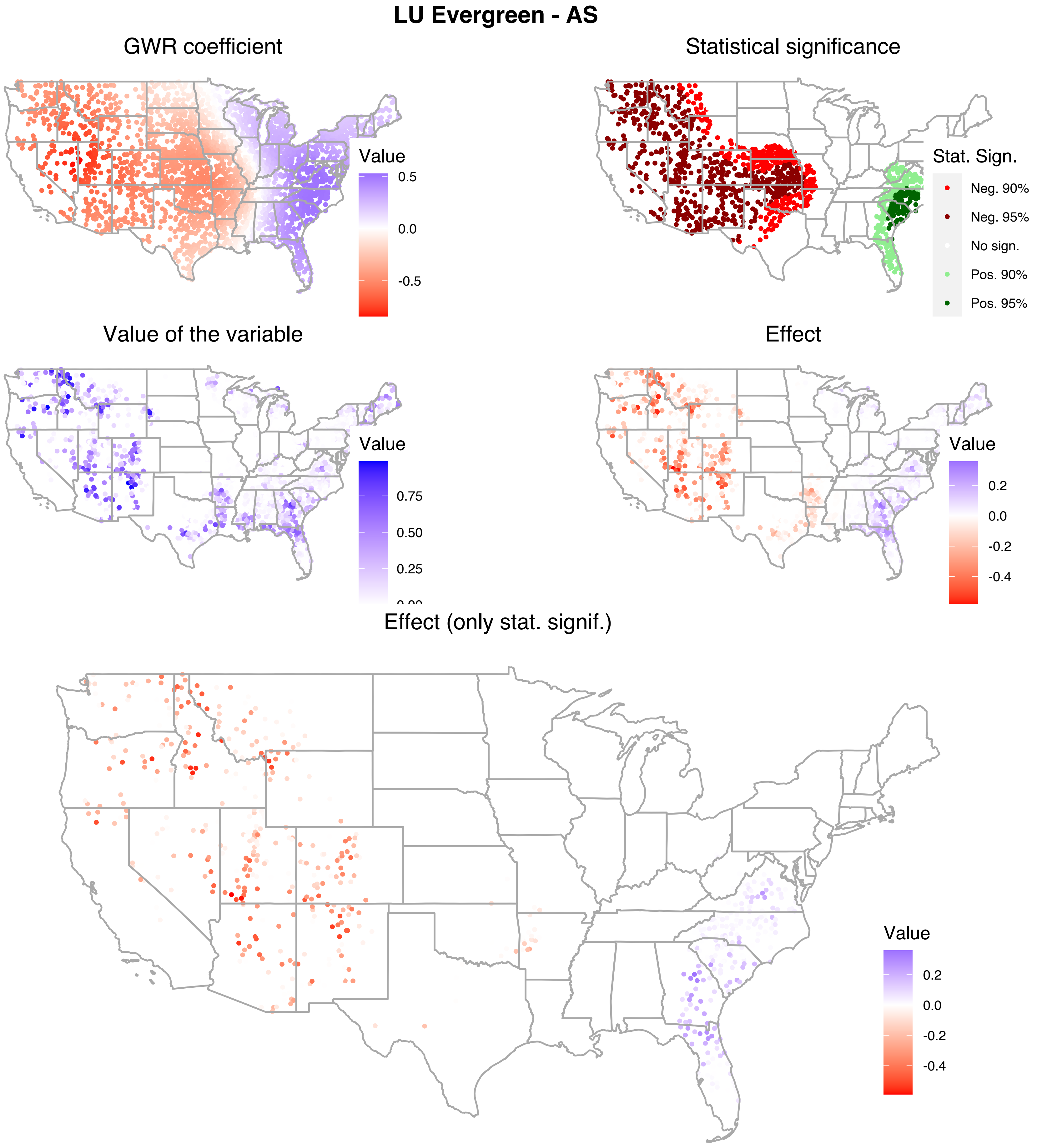
LU Evergreen - AS
GWR coefficient
Value
0.5
0.0
-0.5
Statistical significance
Stat. Sign.
Neg. 90%
Neg. 95%
No sign.
Pos. 90%
Pos. 95%
Value of the variable
Value
0.75
0.50
0.25
Effect
Value
0.2
0.0
-0.2
-0.4
Effect (only stat. signif.)
Value
0.2
0.0
-0.2
-0.4

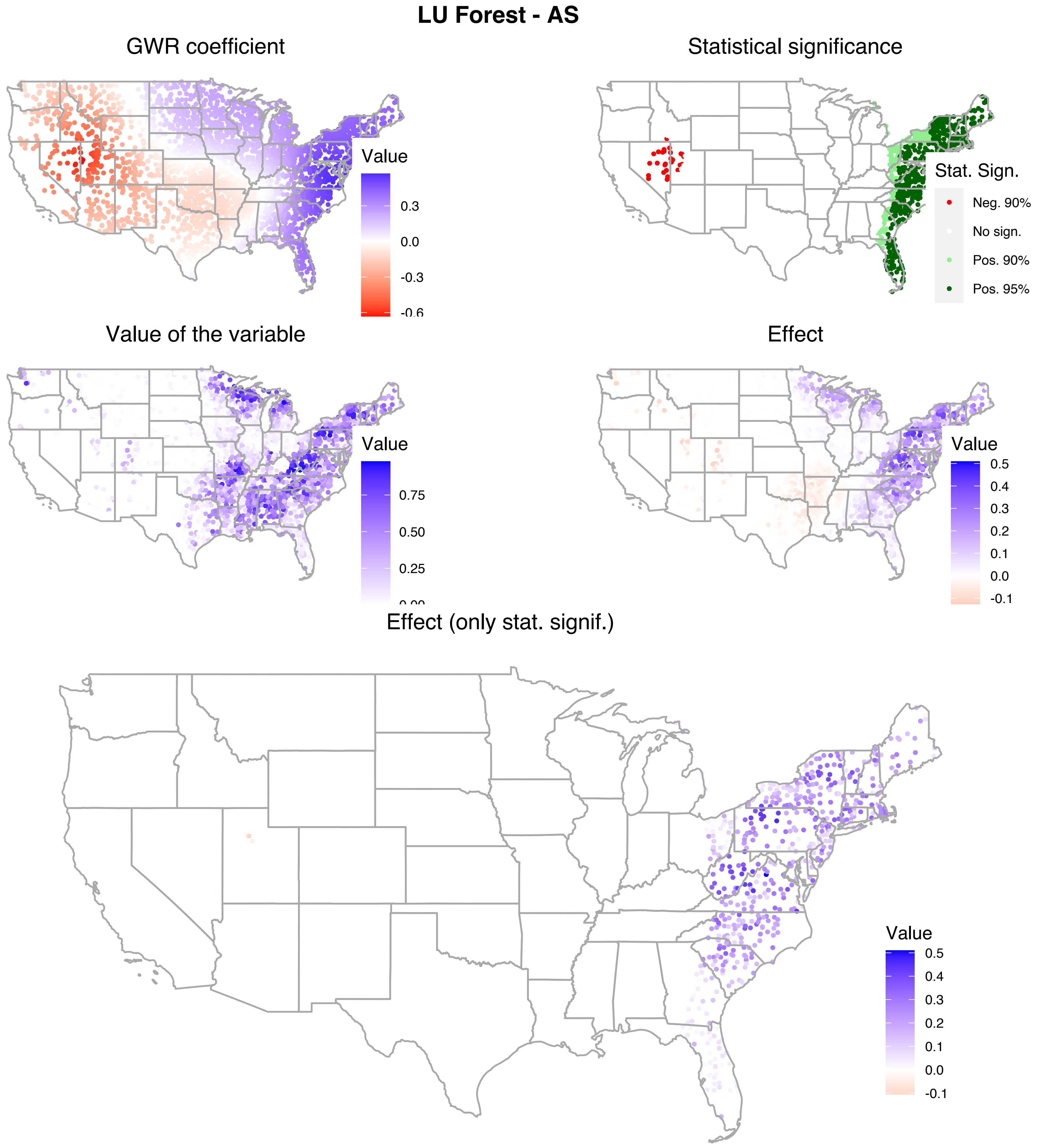
LU Forest - AS
GWR coefficient
Value
0.3
0.0
-0.3
-0.6
Statistical significance
Stat. Sign.
Neg. 90%
No sign.
Pos. 90%
Pos. 95%
Value of the variable
Value
0.75
0.50
0.25
Effect
Value
0.5
0.4
0.3
0.2
0.1
0.0
-0.1
Effect (only stat. signif.)
Value
0.5
0.4
0.3
0.2
0.1
0.0
-0.1

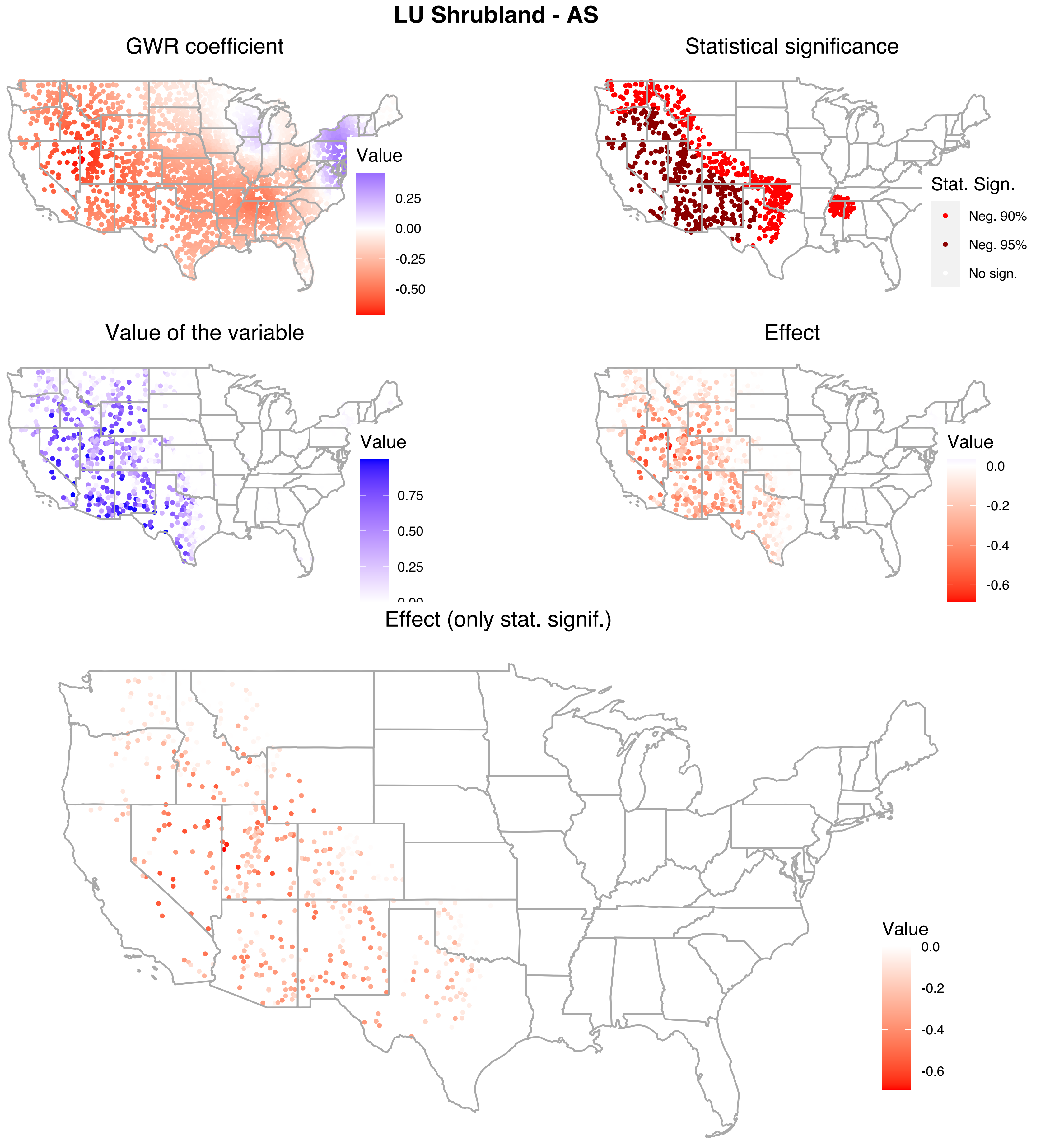

LU Shrubland - AS
GWR coefficient
Value
0.25
0.00
-0.25
-0.50
Statistical significance
Stat. Sign.
Neg. 90%
Neg. 95%
No sign.
Value of the variable
Value
0.75
0.50
0.25
Effect
Value
0.0
-0.2
-0.4
-0.6
Effect (only stat. signif.)
Value
0.0
-0.2
-0.4
-0.6

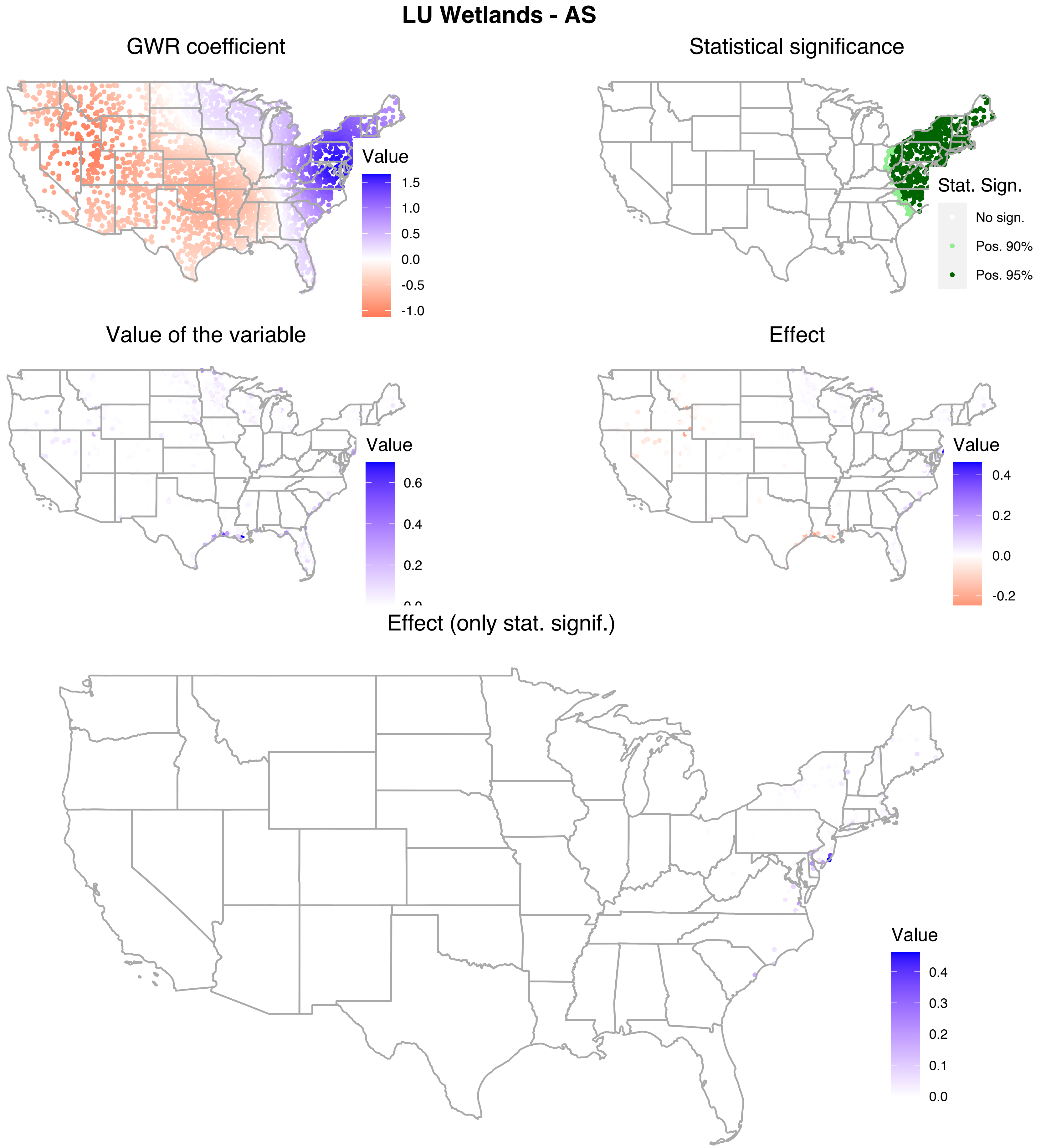

LU Wetlands - AS
GWR coefficient
Value
1.5
1.0
0.5
0.0
-0.5
-1.0
Statistical significance
Stat. Sign.
No sign.
Pos. 90%
Pos. 95%
Value of the variable
Value
0.6
0.4
0.2
Effect
Value
0.4
0.2
0.0
-0.2
Effect (only stat. signif.)
Value
0.4
0.3
0.2
0.1
0.0

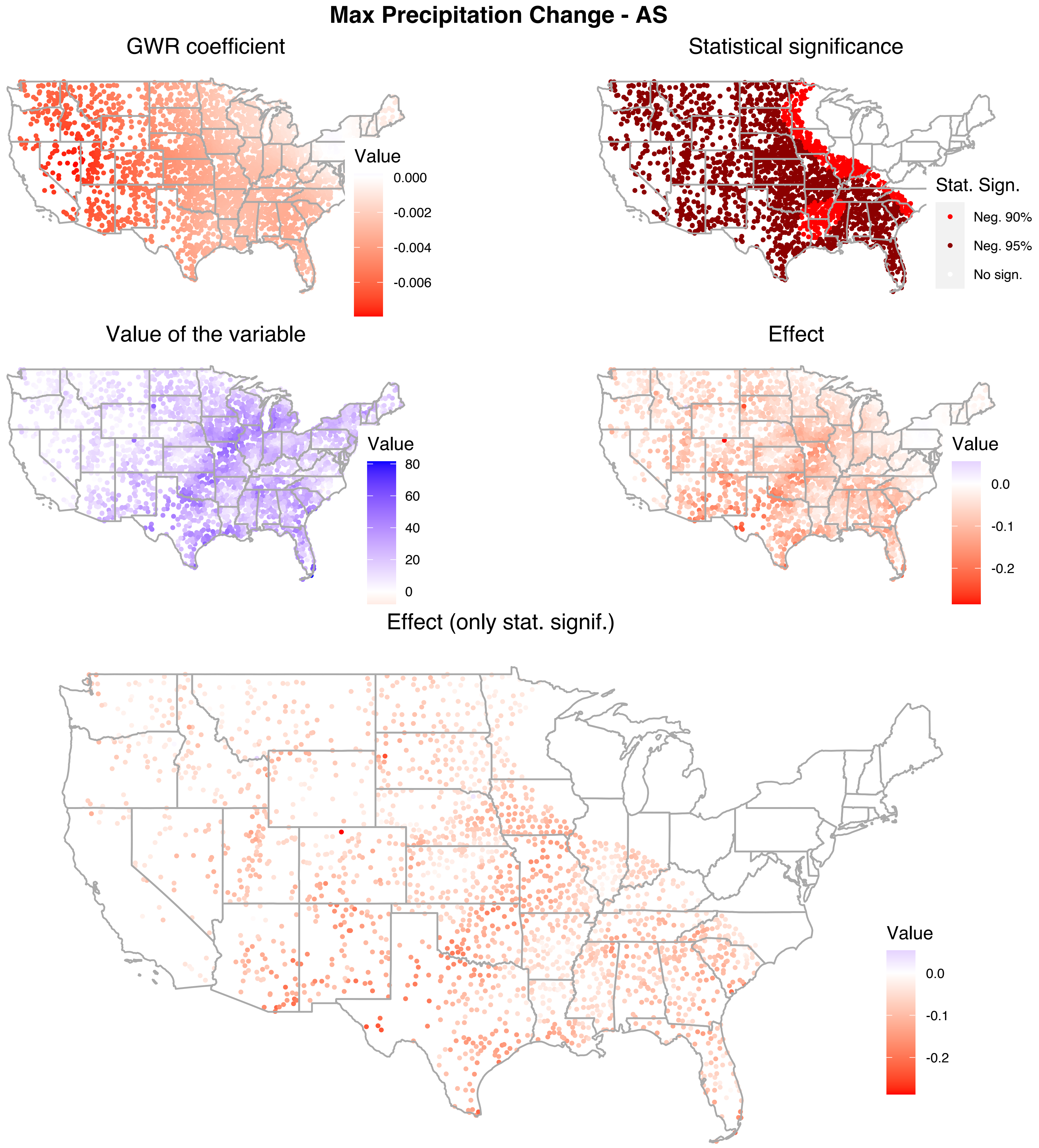

Max Precipitation Change - AS
GWR coefficient
Value
0.000
-0.002
-0.004
-0.006
Statistical significance
Stat. Sign.
Neg. 90%
Neg. 95%
No sign.
Value of the variable
Value
80
60
40
20
0
Effect
Value
0.0
-0.1
-0.2
Effect (only stat. signif.)
Value
0.0
-0.1
-0.2

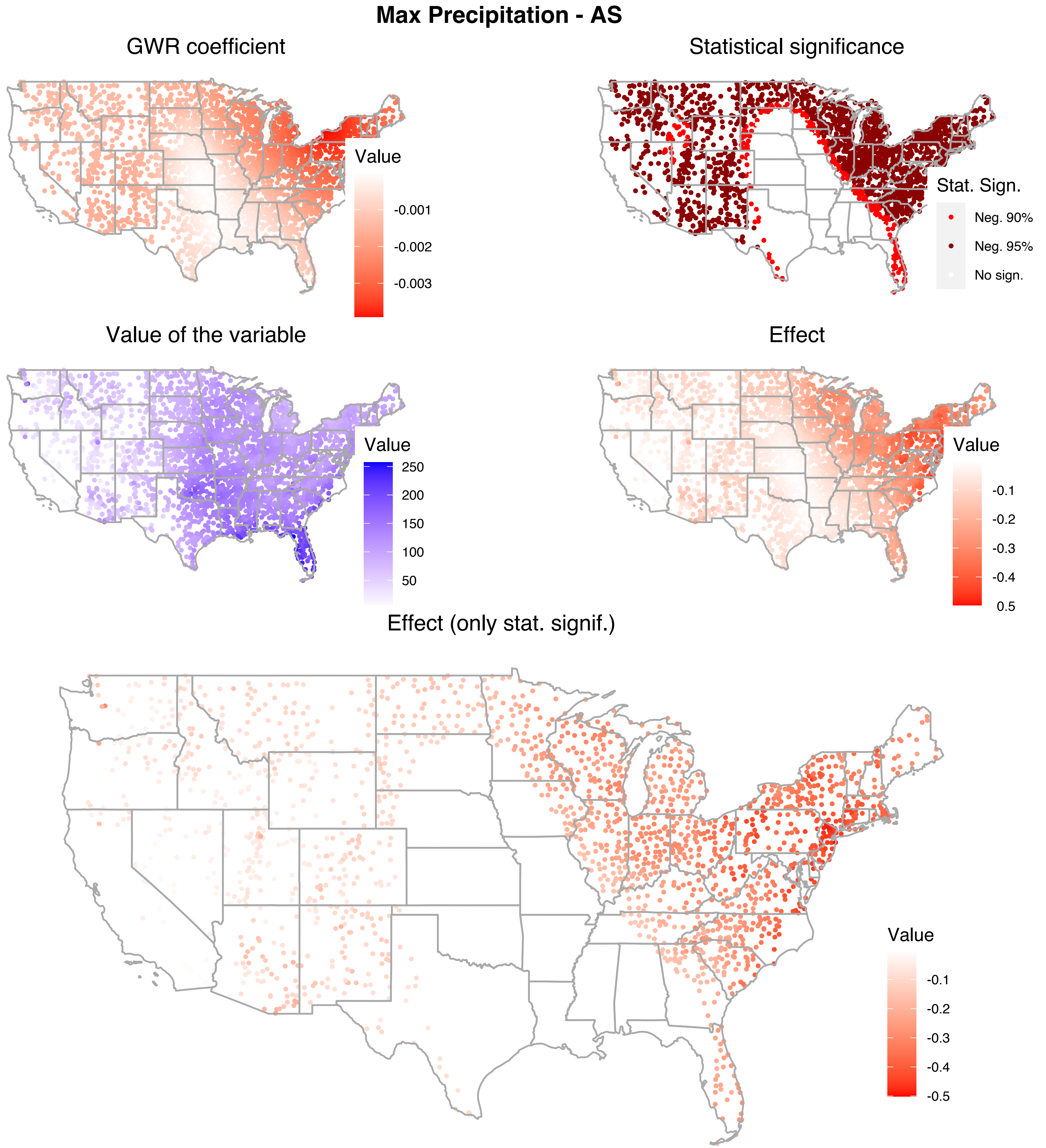
Max Precipitation - AS
GWR coefficient
Value
-0.001
-0.002
-0.003
Statistical significance
Stat. Sign.
Neg. 90%
Neg. 95%
No sign.
Value of the variable
Value
250
200
150
100
50
Effect
Value
-0.1
-0.2
-0.3
-0.4
0.5
Effect (only stat. signif.)
Value
-0.1
-0.2
-0.3
-0.4
-0.5

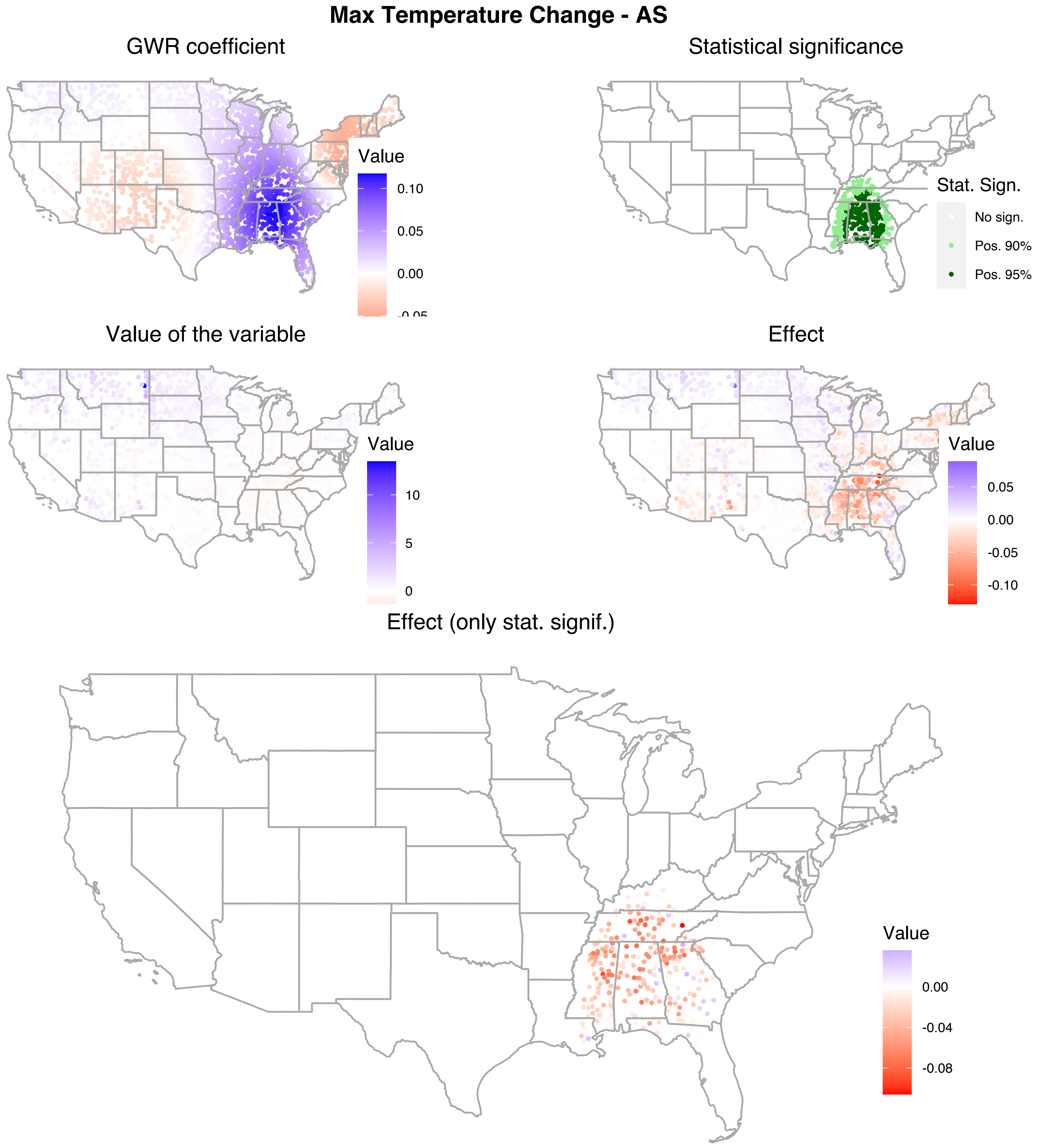

Max Temperature Change - AS
GWR coefficient
Value
0.10
0.05
0.00
-0.05
Statistical significance
Stat. Sign.
No sign.
Pos. 90%
Pos. 95%
Value of the variable
Value
10
5
0
Effect
Value
0.05
0.00
-0.05
-0.10
Effect (only stat. signif.)
Value
0.00
-0.04
-0.08

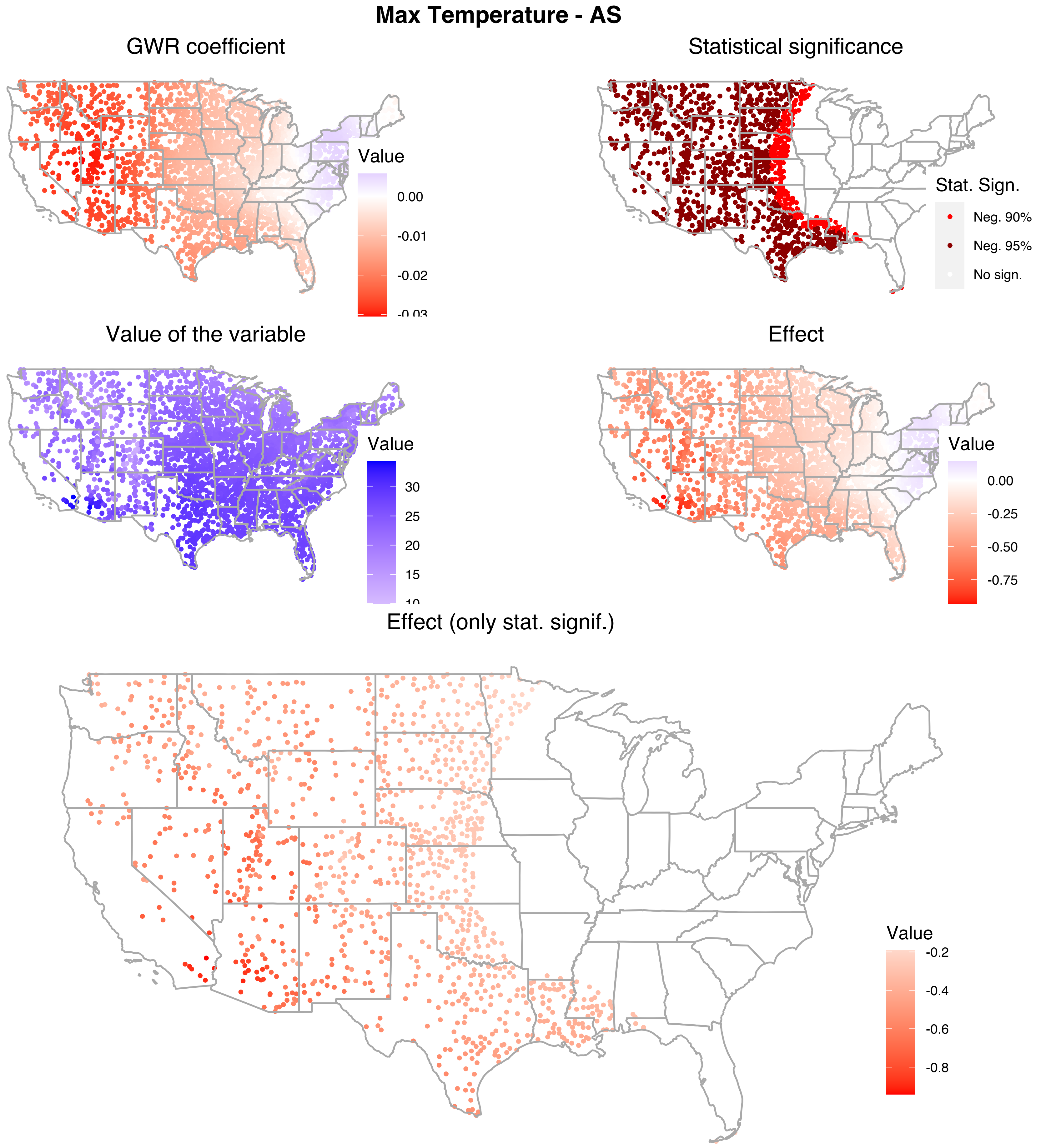

Max Temperature - AS
GWR coefficient
Value
0.00
-0.01
-0.02
Statistical significance
Stat. Sign.
Neg. 90%
Neg. 95%
No sign.
Value of the variable
Value
30
25
20
15
Effect
Value
0.00
-0.25
-0.50
-0.75
Effect (only stat. signif.)
Value
-0.2
-0.4
-0.6
-0.8

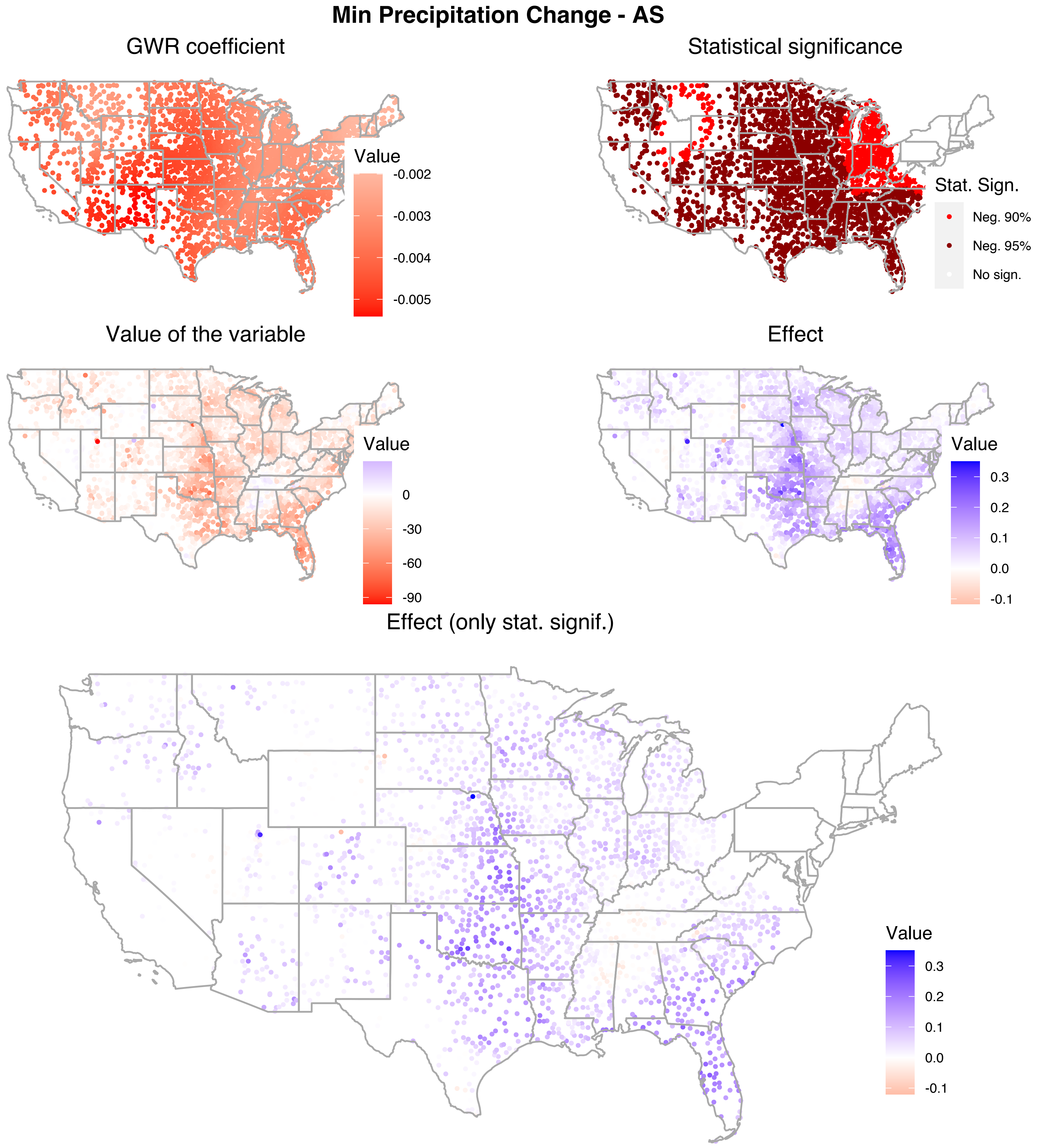

Min Precipitation Change - AS
GWR coefficient
Value
-0.002
-0.003
-0.004
-0.005
Statistical significance
Stat. Sign.
Neg. 90%
Neg. 95%
No sign.
Value of the variable
Value
0
-30
-60
-90
Effect
Value
0.3
0.2
0.1
0.0
-0.1
Effect (only stat. signif.)
Value
0.3
0.2
0.1
0.0
-0.1

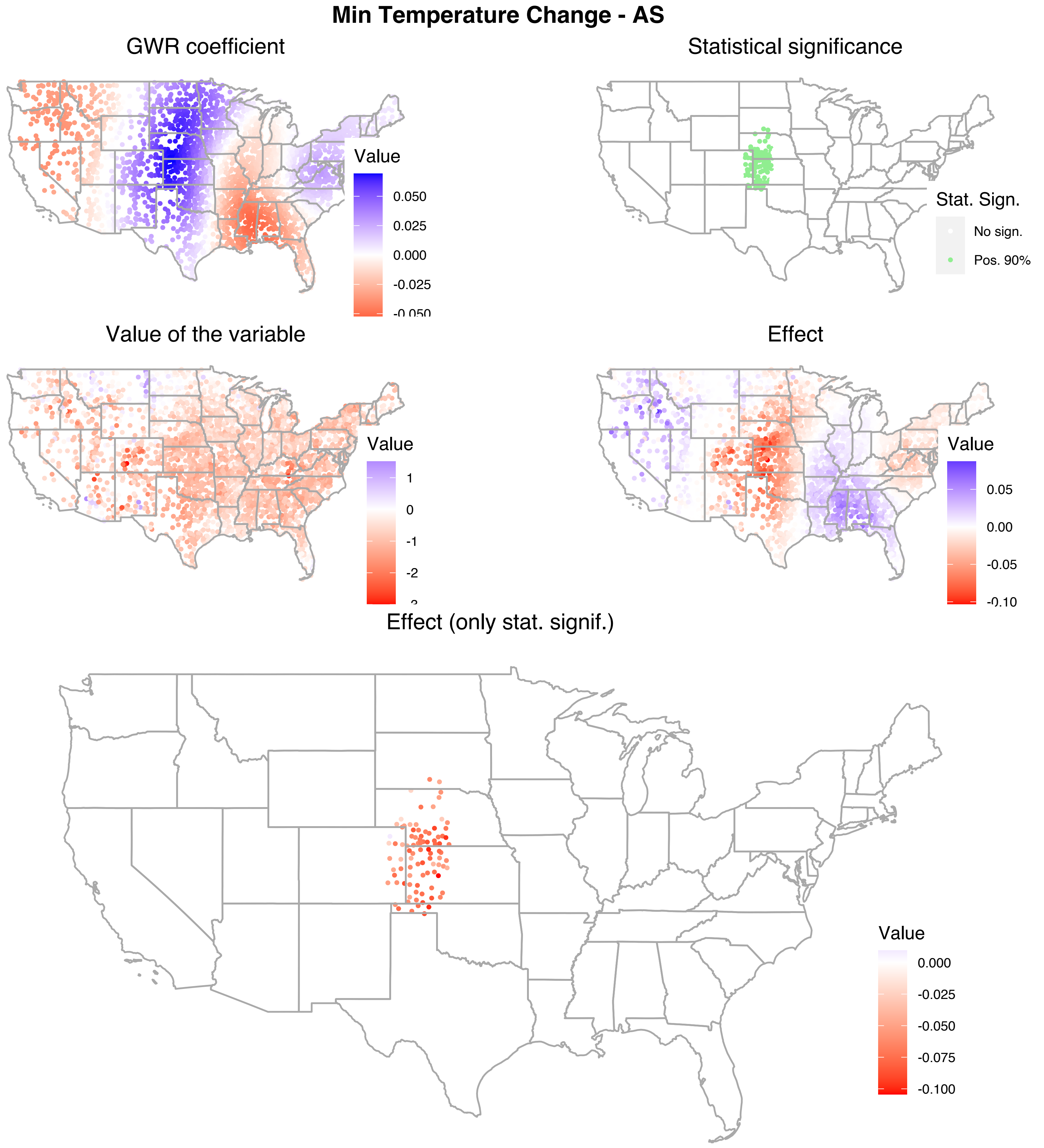
Min Temperature Change - AS
GWR coefficient
Value
0.050
0.025
0.000
-0.025
Statistical significance
Stat. Sign.
No sign.
Pos. 90%
Value of the variable
Value
1
0
-1
-2
Effect
Value
0.05
0.00
-0.05
-0.10
Effect (only stat. signif.)
Value
0.000
-0.025
-0.050
-0.075
-0.100
