# Supplementary material for: Climatic and landscape changes as drivers of environmental feedback that influence rainfall frequency in the United States
Source: Glob Chang Biol. 2021 Sep 23;27(24):6381–93. doi: 10.1111/gcb.15876 (PMC9292682; doi:10.1111/gcb.15876)

Average Precipitation Change - OM

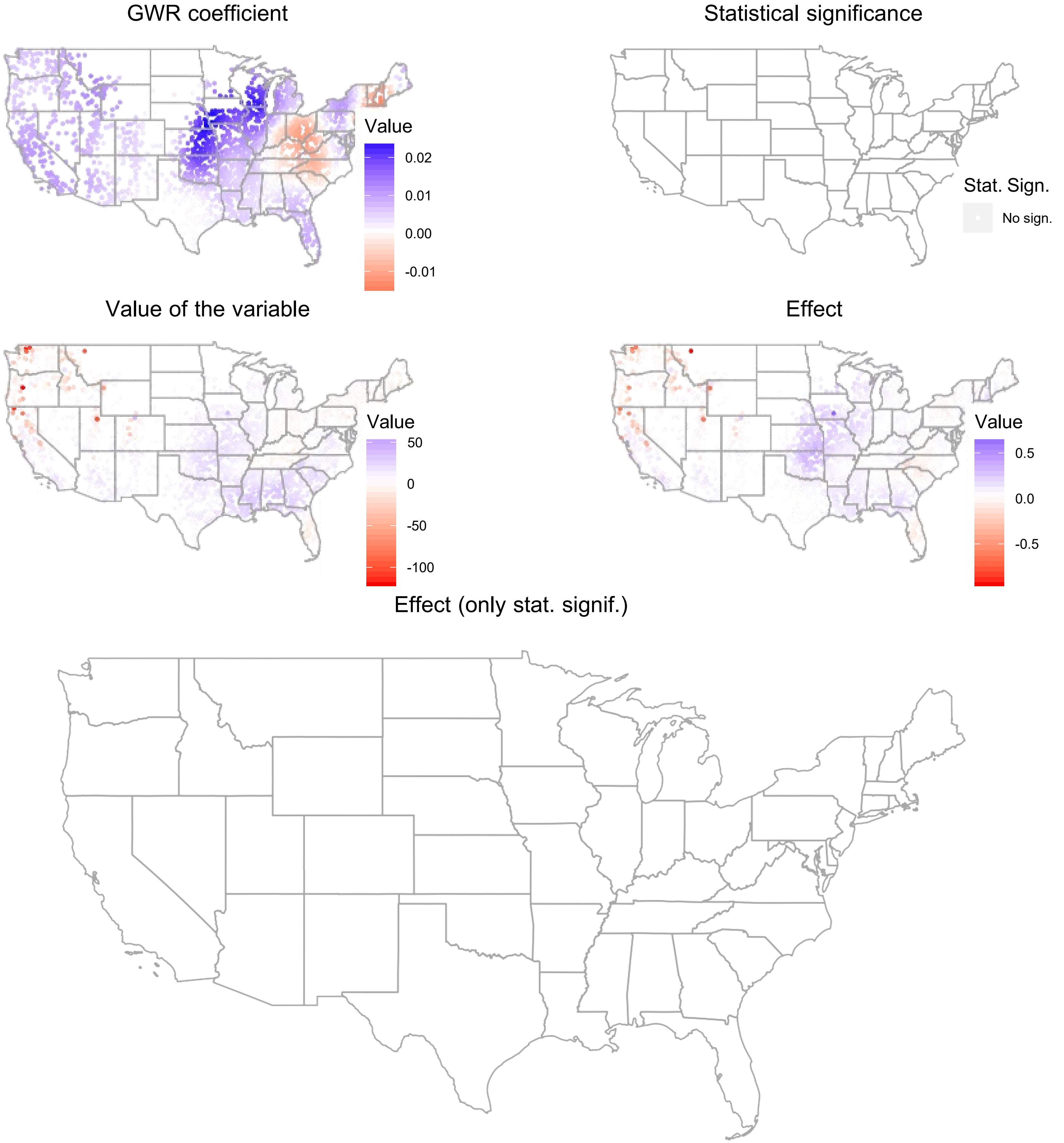

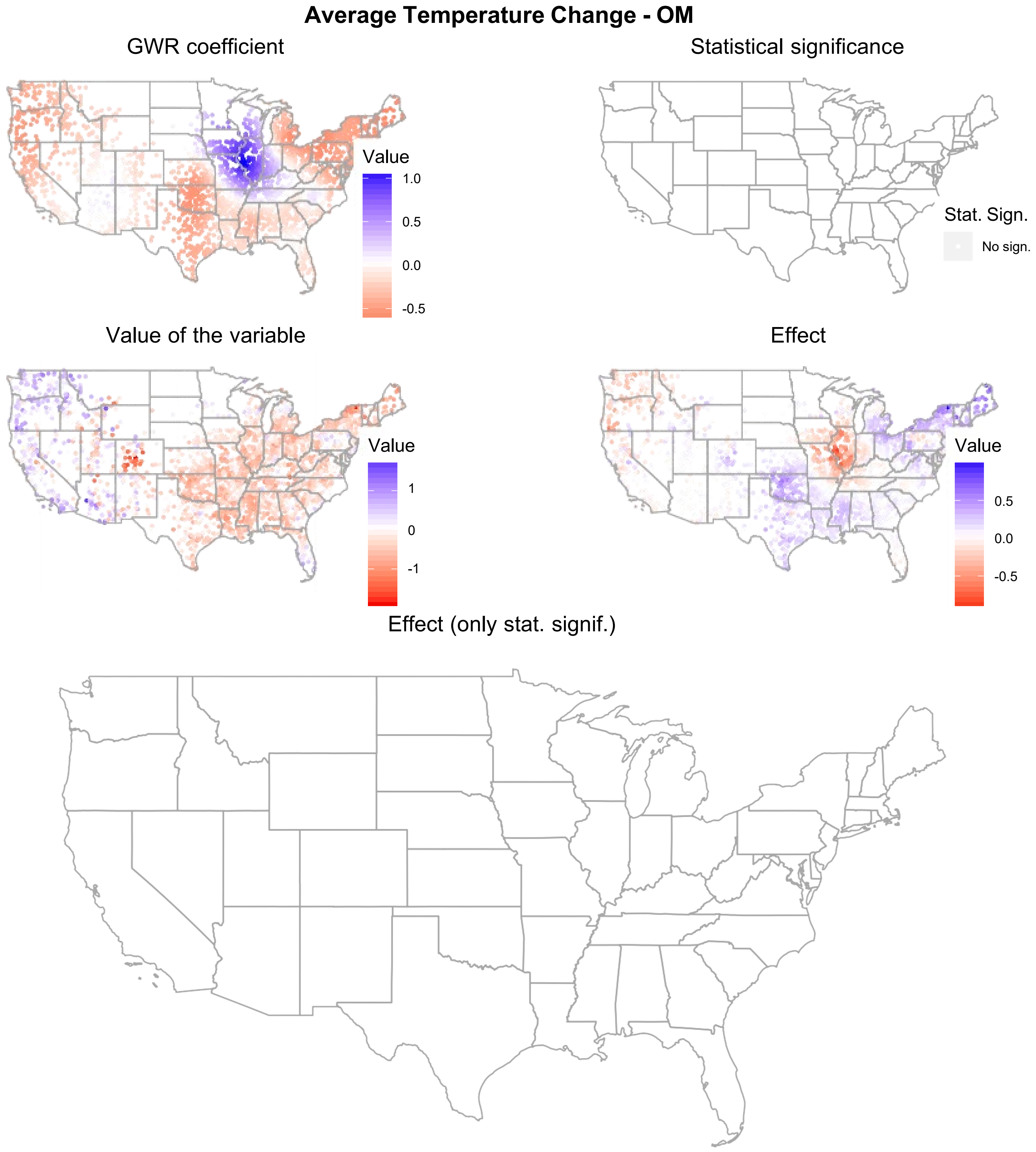
Average Temperature Change - OM
GWR coefficient
Value
1.0
0.5
0.0
-0.5
Statistical significance
Stat. Sign.
No sign.
Value of the variable
Value
1
0
-1
Effect
Value
0.5
0.0
-0.5
Effect (only stat. signif.)

Average Temperature - OM

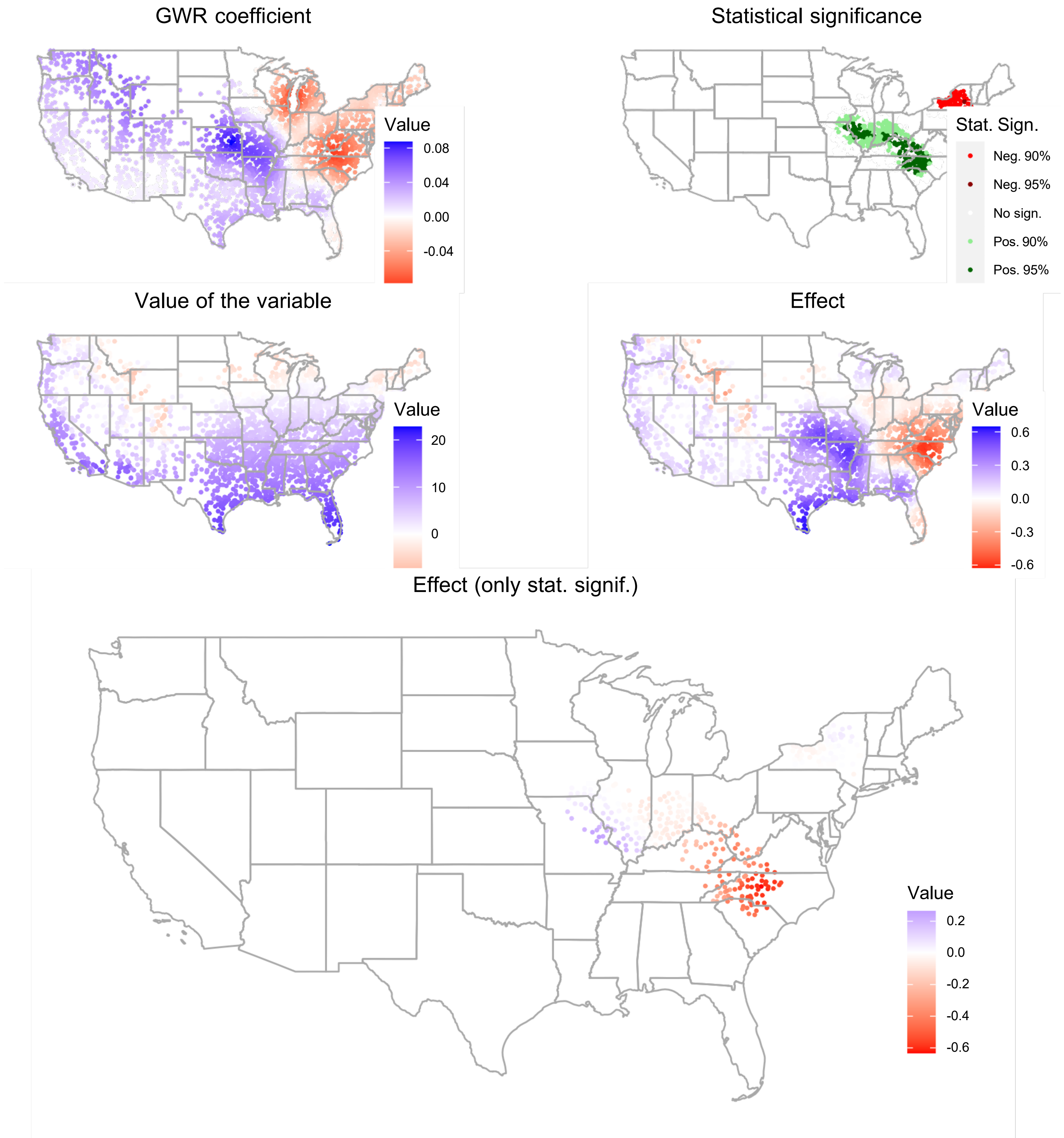

Change LU Agriculture and Herbaceous - OM

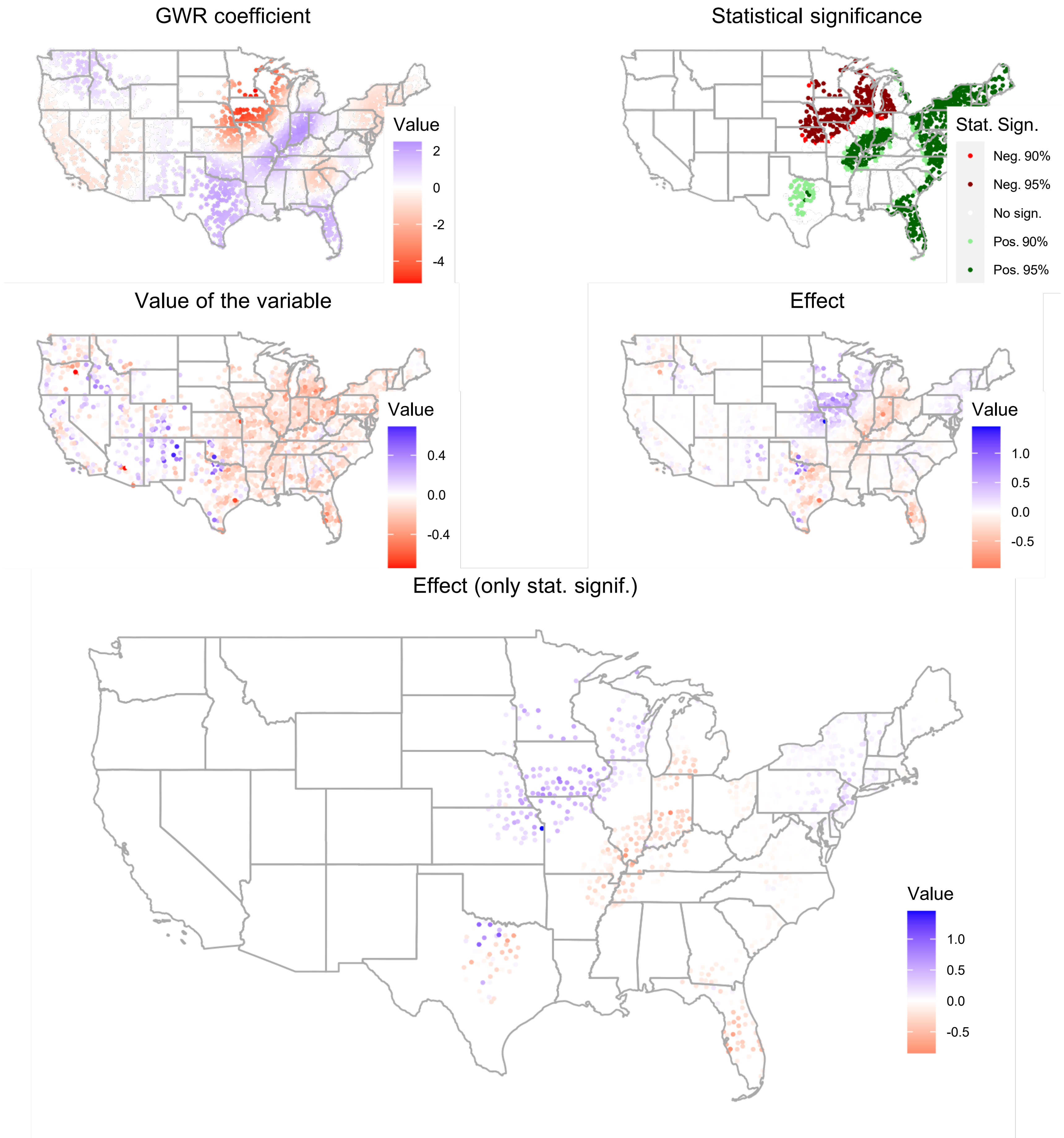

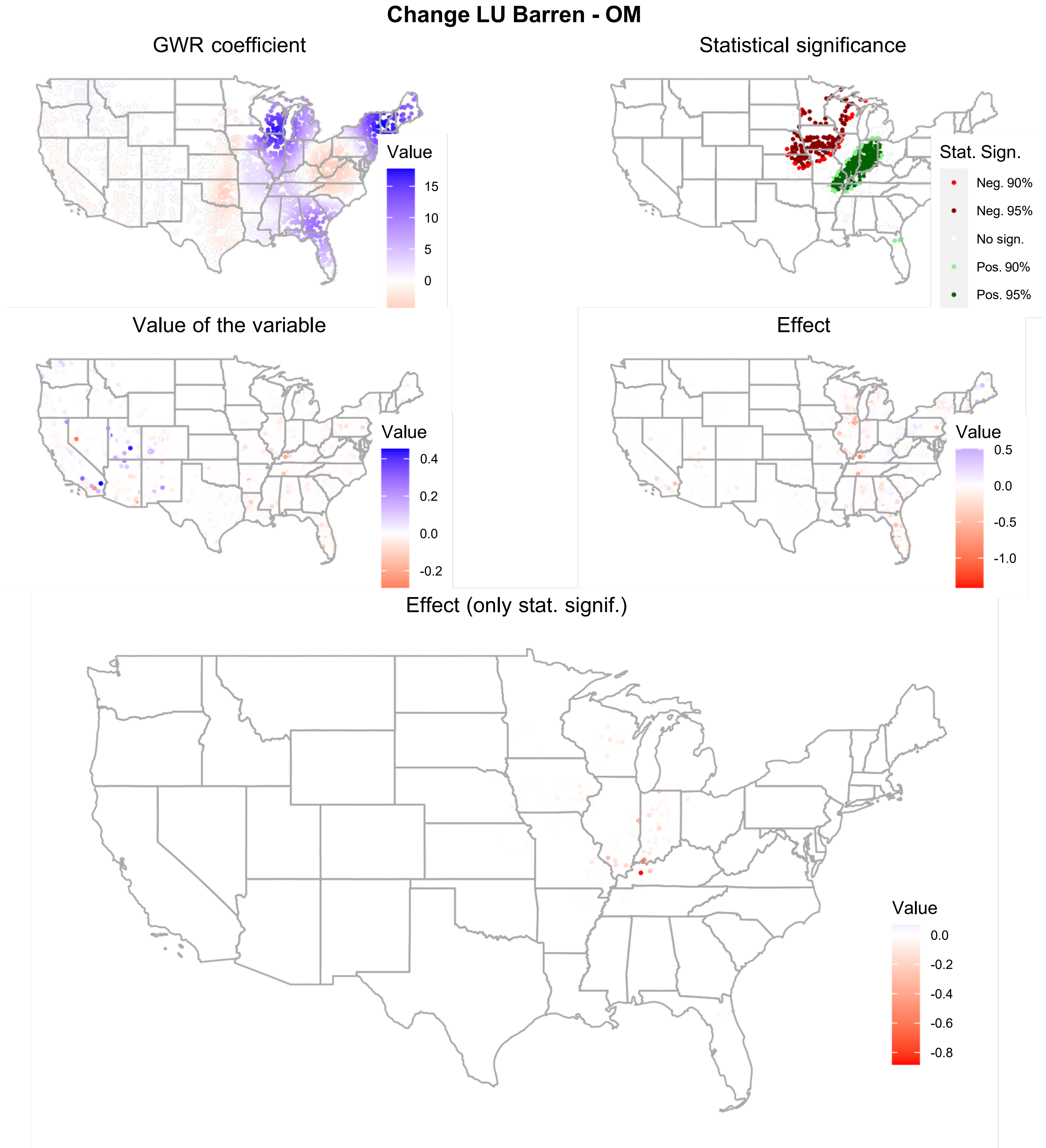

Change LU Barren - OM
GWR coefficient
Value
15
10
5
0
Statistical significance
Stat. Sign.
Neg. 90%
Neg. 95%
No sign.
Pos. 90%
Pos. 95%
Value of the variable
Value
0.4
0.2
0.0
-0.2
Effect
Value
0.5
0.0
-0.5
-1.0
Effect (only stat. signif.)
Value
0.0
-0.2
-0.4
-0.6
-0.8

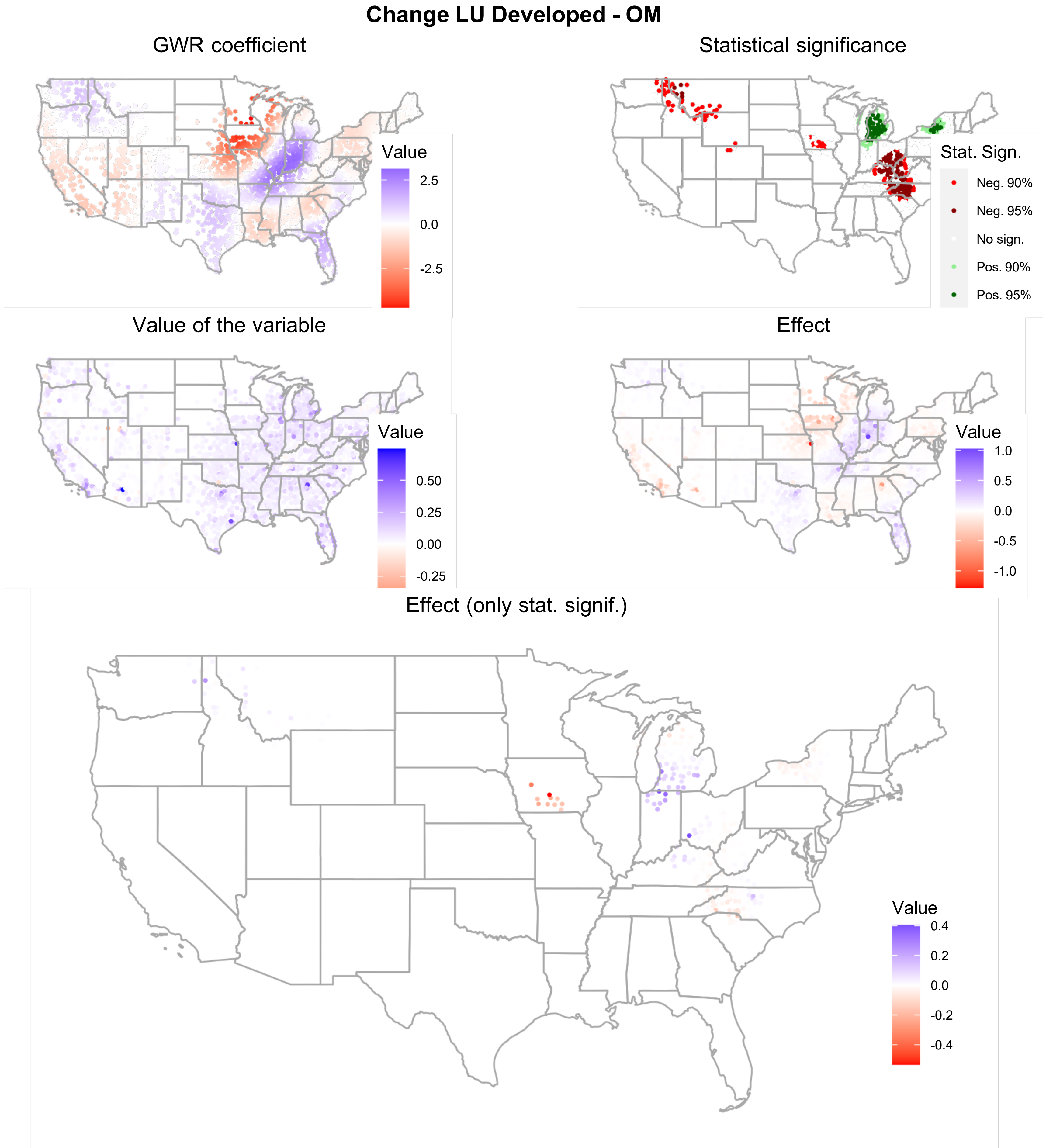

Change LU Developed - OM
GWR coefficient
Value
2.5
0.0
-2.5
Statistical significance
Stat. Sign.
Neg. 90%
Neg. 95%
No sign.
Pos. 90%
Pos. 95%
Value of the variable
Value
0.50
0.25
0.00
-0.25
Effect
Value
1.0
0.5
0.0
-0.5
-1.0
Effect (only stat. signif.)
Value
0.4
0.2
0.0
-0.2
-0.4

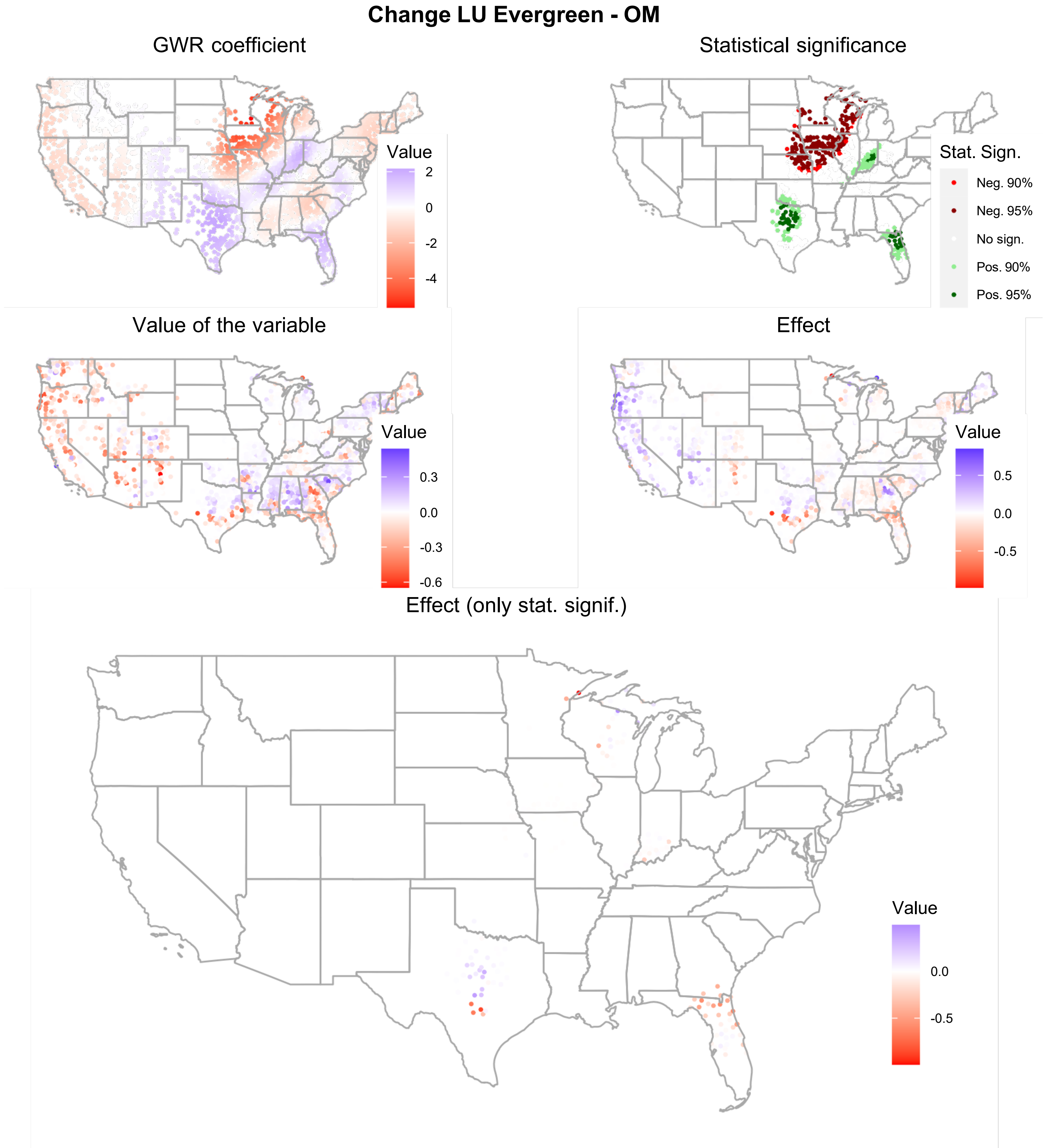
Change LU Evergreen - OM
GWR coefficient
Value
2
0
-2
-4
Statistical significance
Stat. Sign.
Neg. 90%
Neg. 95%
No sign.
Pos. 90%
Pos. 95%
Value of the variable
Value
0.3
0.0
-0.3
-0.6
Effect
Value
0.5
0.0
-0.5
Effect (only stat. signif.)
Value
0.0
-0.5

Change LU Forest - OM

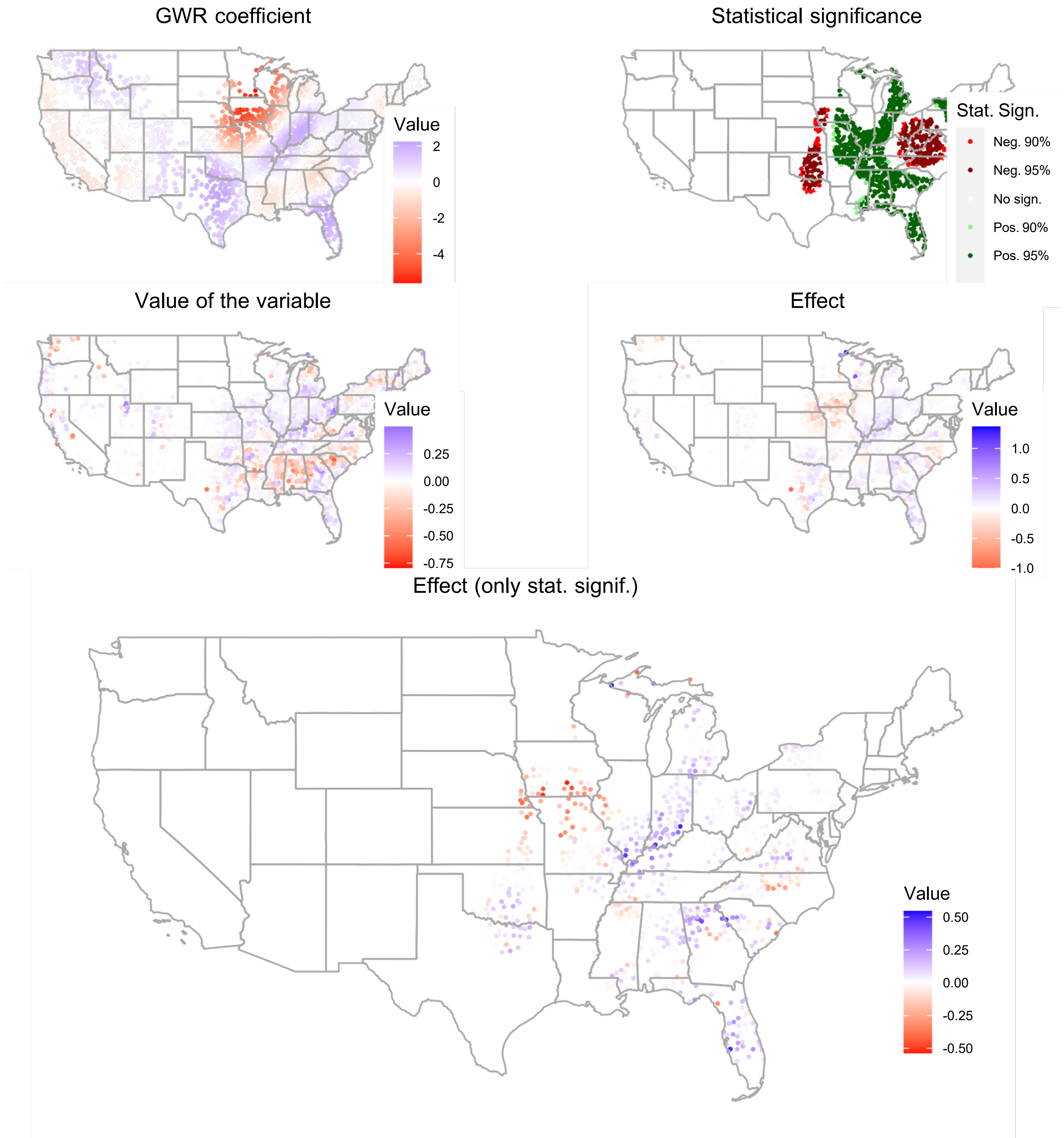

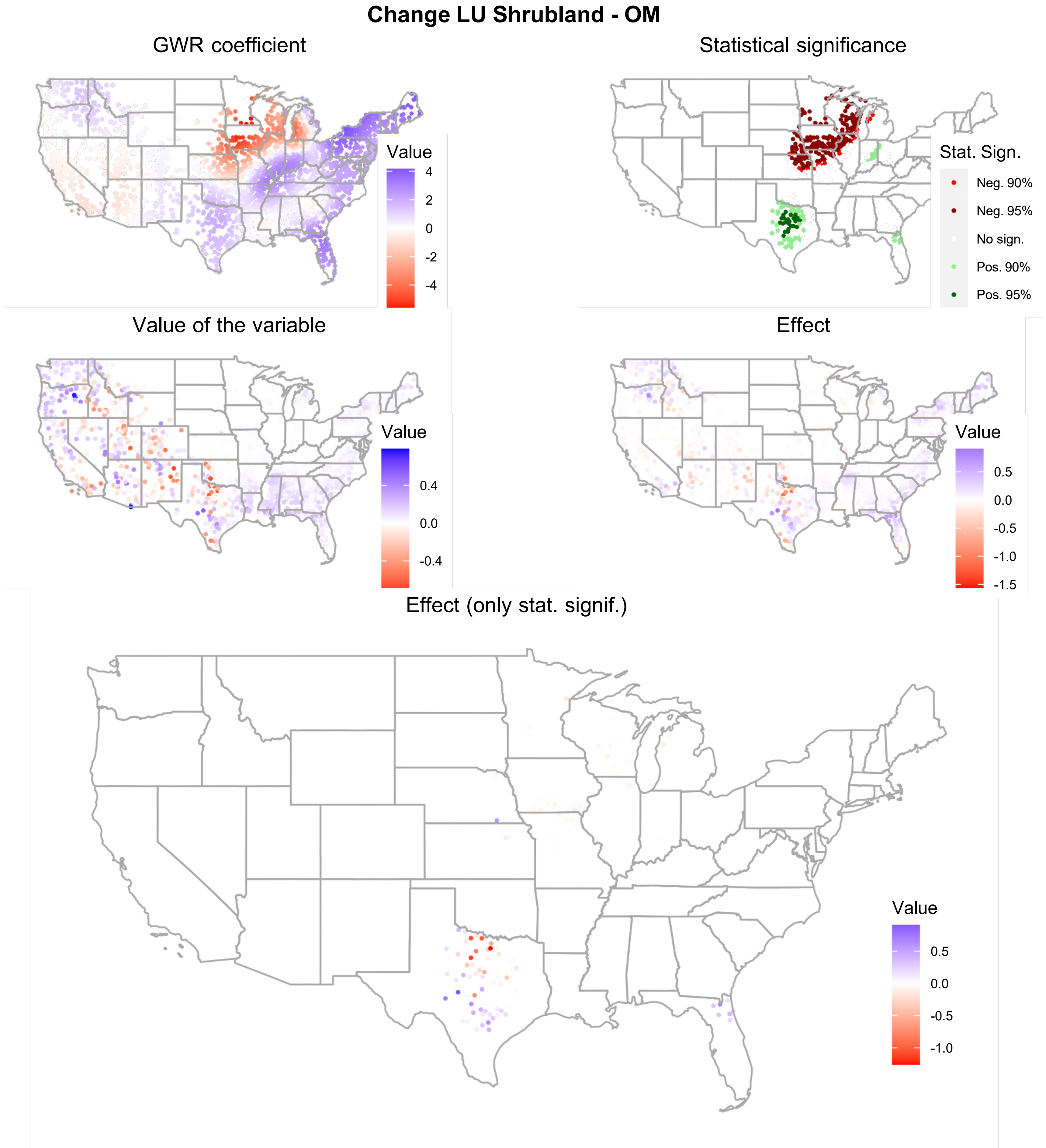
Change LU Shrubland - OM
GWR coefficient
Value
4
2
0
-2
-4
Statistical significance
Stat. Sign.
Neg. 90%
Neg. 95%
No sign.
Pos. 90%
Pos. 95%
Value of the variable
Value
0.4
0.0
-0.4
Effect
Value
0.5
0.0
-0.5
-1.0
-1.5
Effect (only stat. signif.)
Value
0.5
0.0
-0.5
-1.0

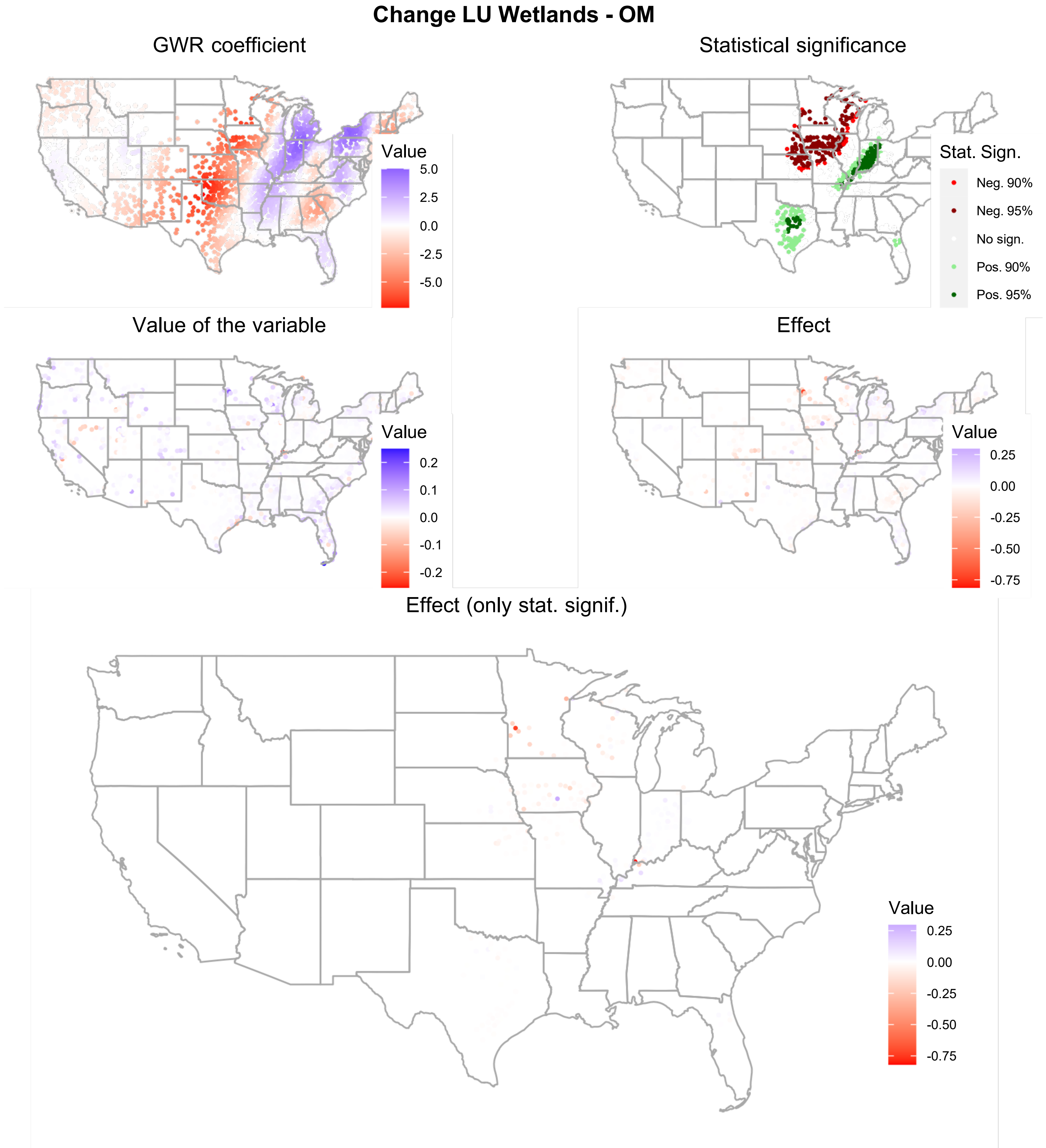
Change LU Wetlands - OM
GWR coefficient
Value
5.0
2.5
0.0
-2.5
-5.0
Statistical significance
Stat. Sign.
Neg. 90%
Neg. 95%
No sign.
Pos. 90%
Pos. 95%
Value of the variable
Value
0.2
0.1
0.0
-0.1
-0.2
Effect
Value
0.25
0.00
-0.25
-0.50
-0.75
Effect (only stat. signif.)
Value
0.25
0.00
-0.25
-0.50
-0.75

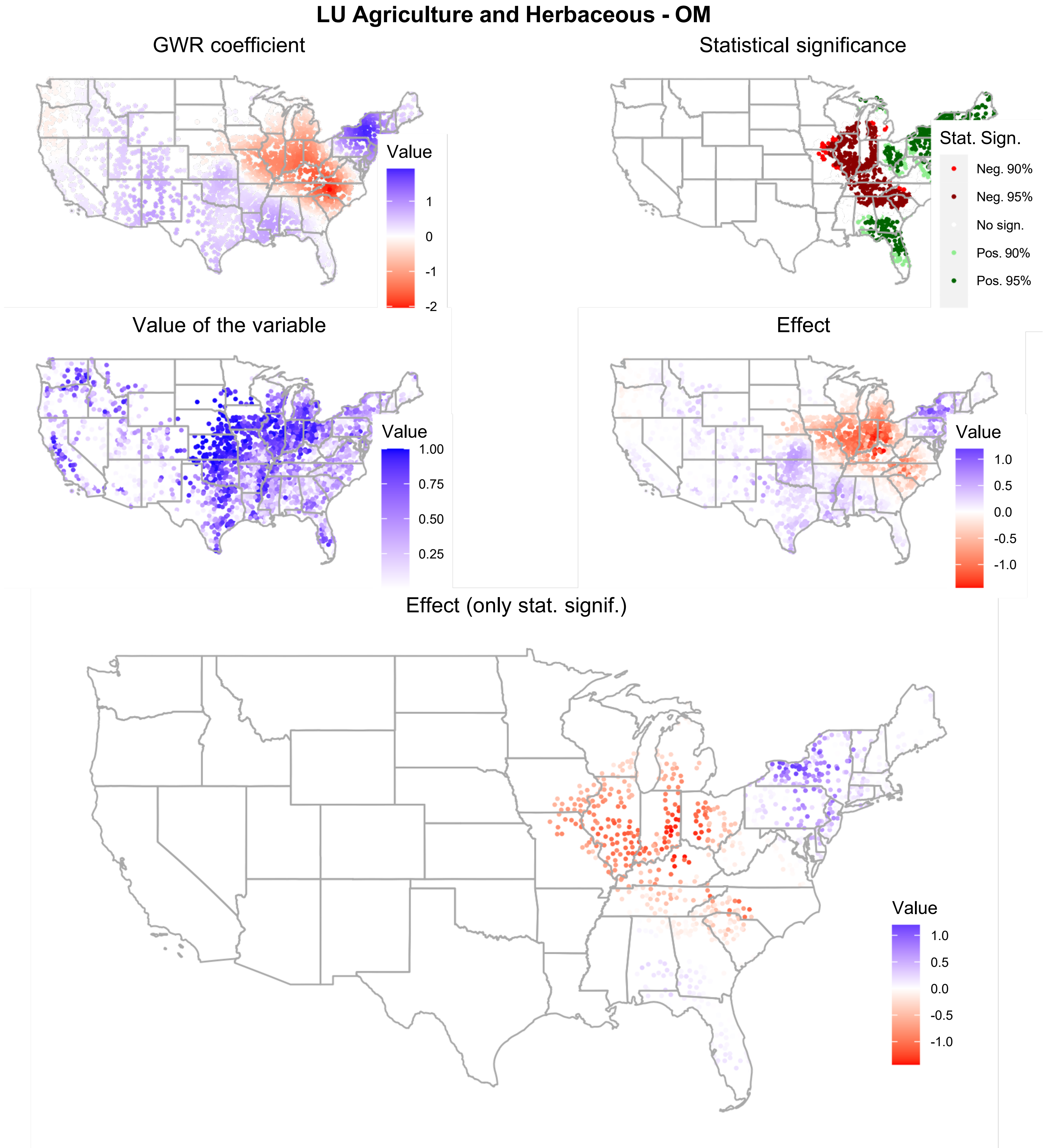

LU Agriculture and Herbaceous - OM
GWR coefficient
Value
1
0
-1
-2
Statistical significance
Stat. Sign.
Neg. 90%
Neg. 95%
No sign.
Pos. 90%
Pos. 95%
Value of the variable
Value
1.00
0.75
0.50
0.25
Effect
Value
1.0
0.5
0.0
-0.5
-1.0
Effect (only stat. signif.)
Value
1.0
0.5
0.0
-0.5
-1.0

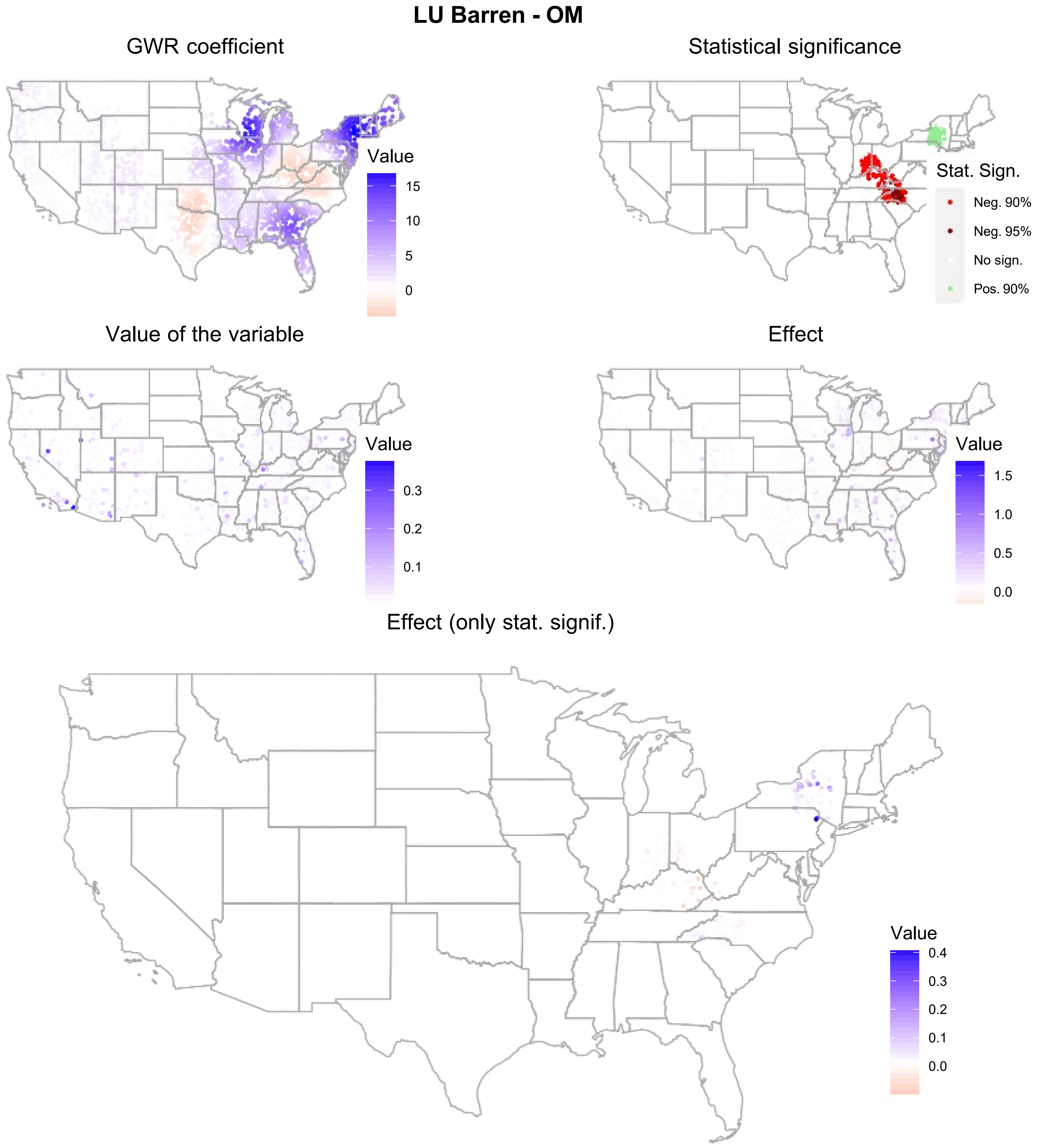

LU Barren - OM
GWR coefficient
Value
15
10
5
0
Statistical significance
Stat. Sign.
Neg. 90%
Neg. 95%
No sign.
Pos. 90%
Value of the variable
Value
0.3
0.2
0.1
Effect
Value
1.5
1.0
0.5
0.0
Effect (only stat. signif.)
Value
0.4
0.3
0.2
0.1
0.0

LU Developed - OM

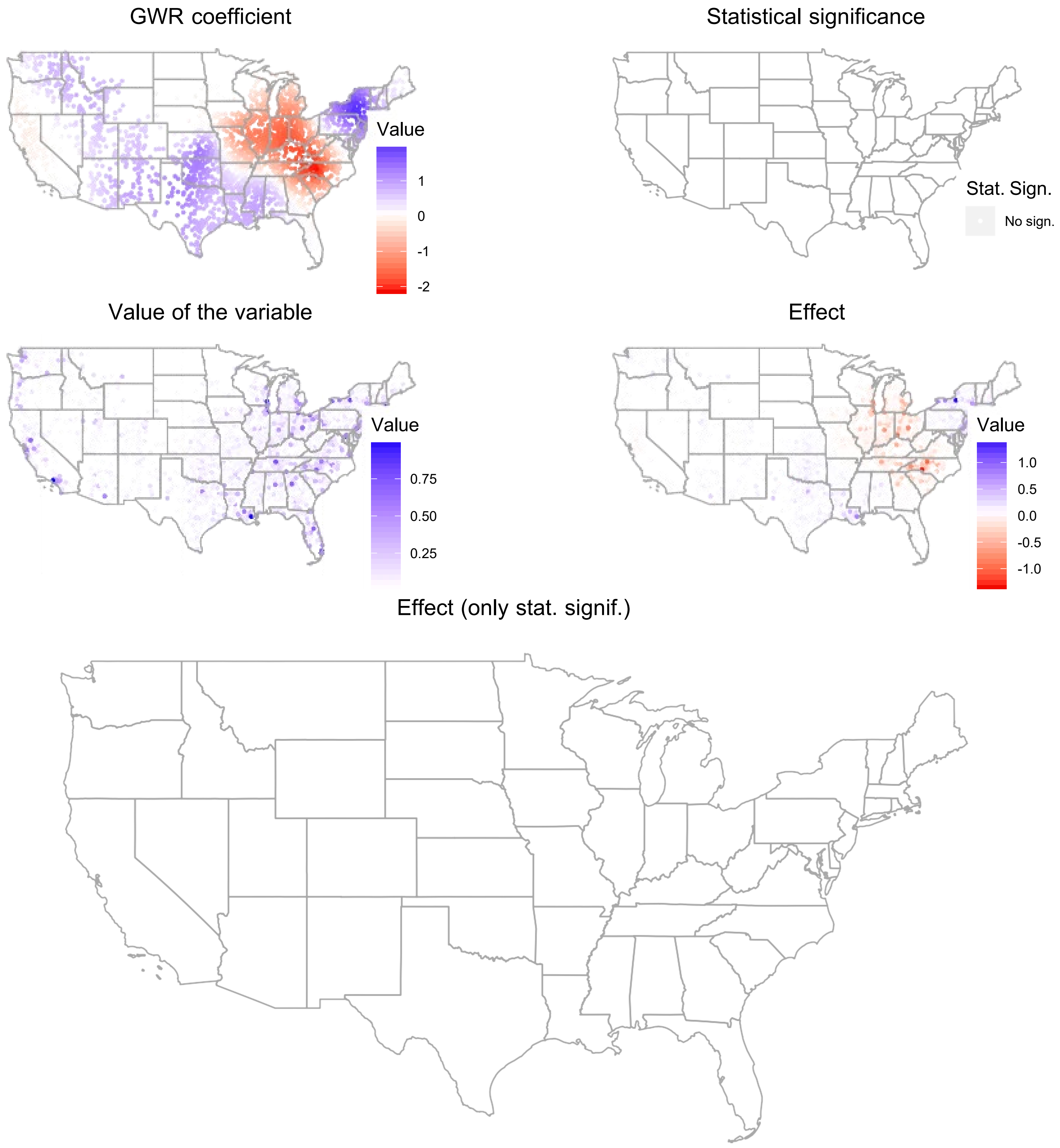

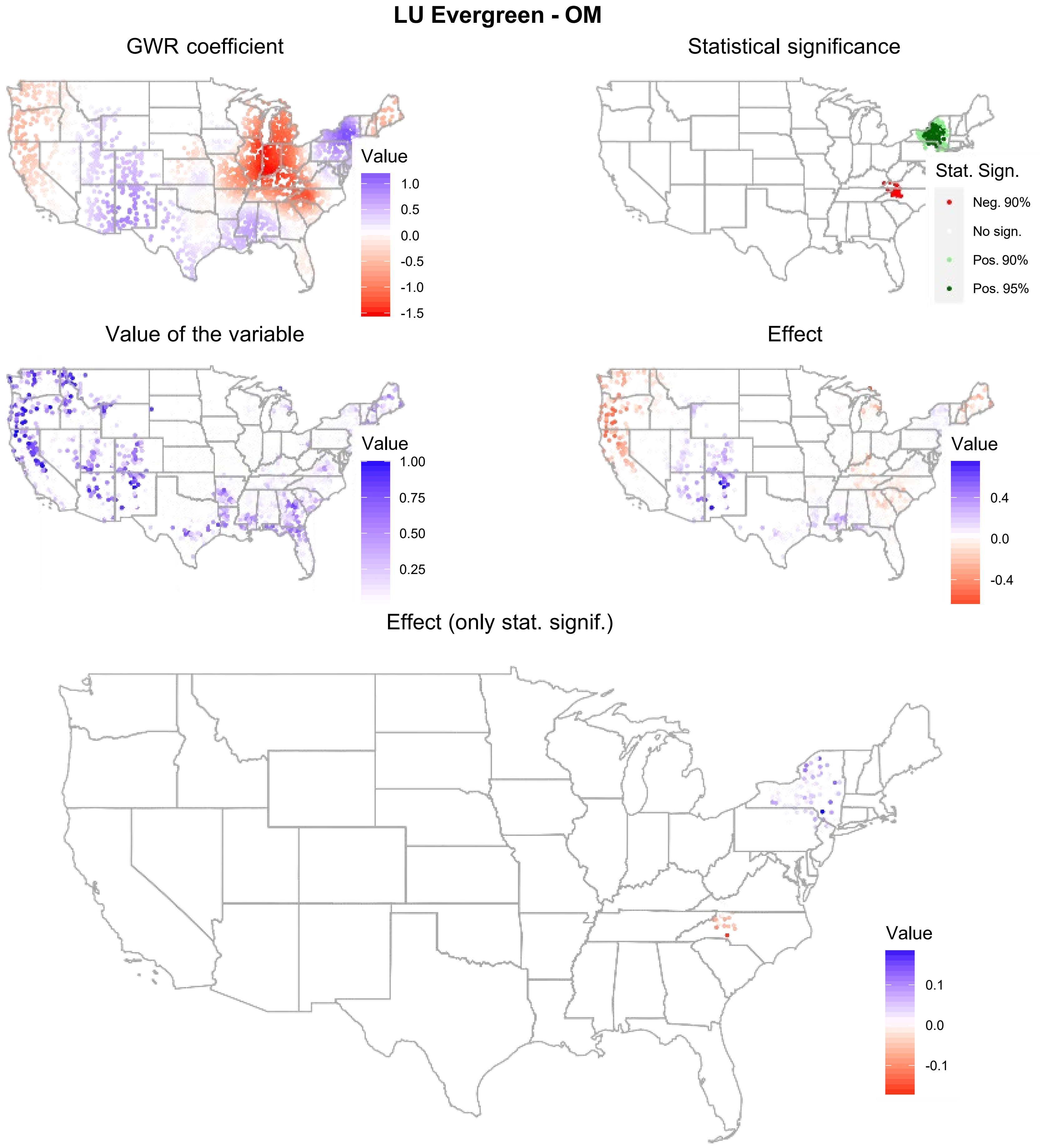
LU Evergreen - OM
GWR coefficient
Value
1.0
0.5
0.0
-0.5
-1.0
-1.5
Statistical significance
Stat. Sign.
Neg. 90%
No sign.
Pos. 90%
Pos. 95%
Value of the variable
Value
1.00
0.75
0.50
0.25
Effect
Value
0.4
0.0
-0.4
Effect (only stat. signif.)
Value
0.1
0.0
-0.1

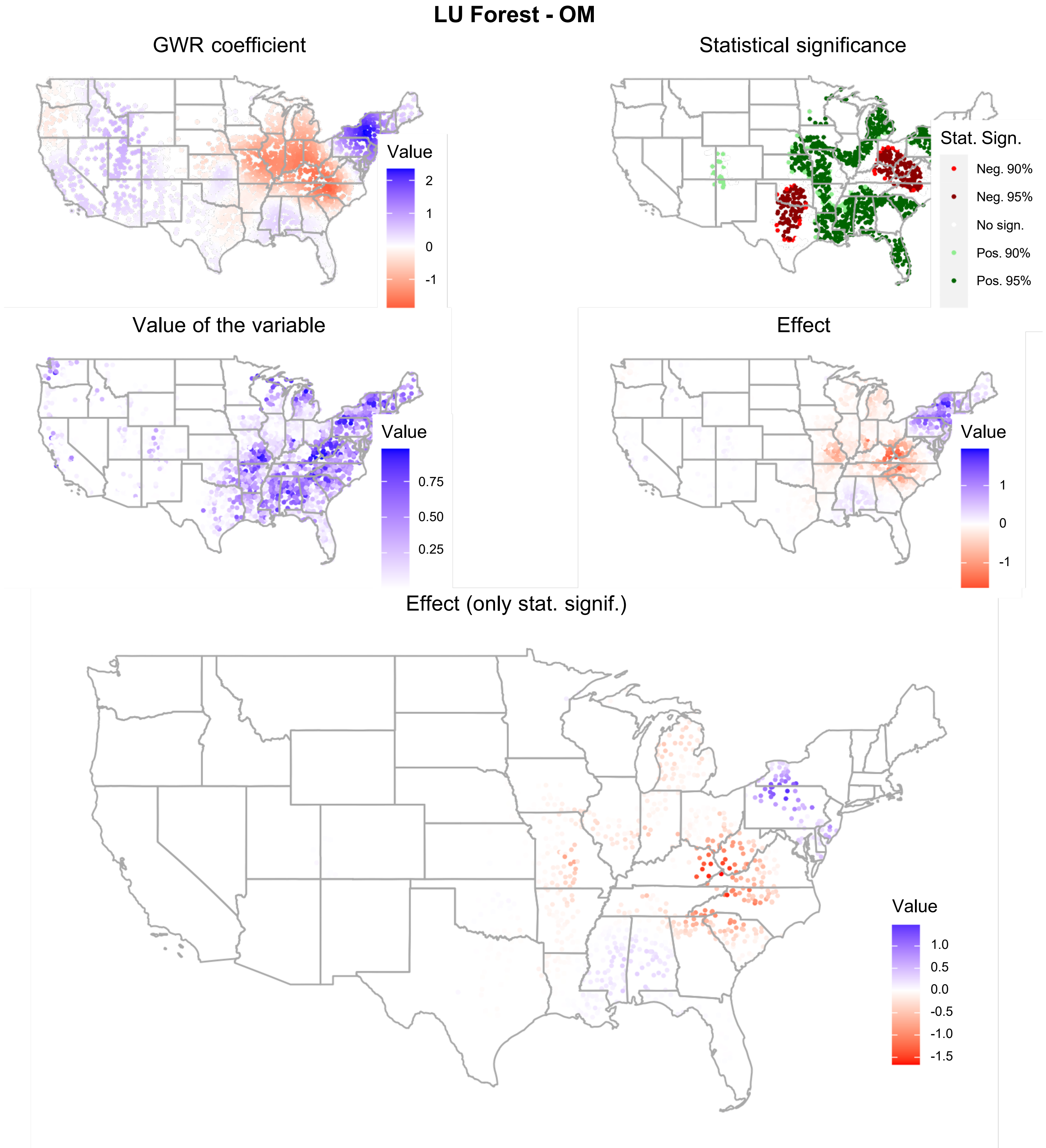

LU Forest - OM
GWR coefficient
Value
2
1
0
-1
Statistical significance
Stat. Sign.
Neg. 90%
Neg. 95%
No sign.
Pos. 90%
Pos. 95%
Value of the variable
Value
0.75
0.50
0.25
Effect
Value
1
0
-1
Effect (only stat. signif.)
Value
1.0
0.5
0.0
-0.5
-1.0
-1.5

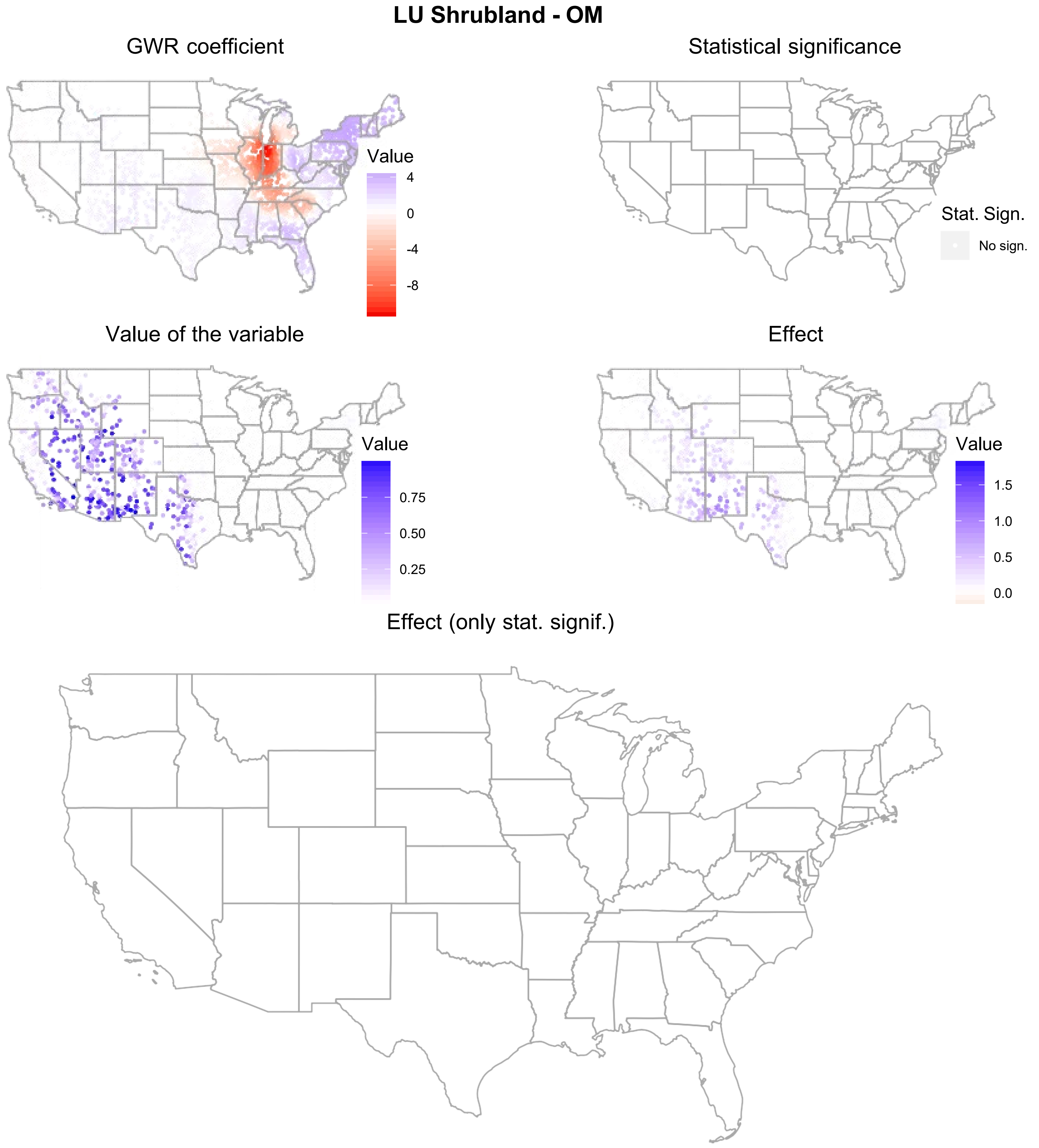
LU Shrubland - OM
GWR coefficient
Value
4
0
-4
-8
Statistical significance
Stat. Sign.
No sign.
Value of the variable
Value
0.75
0.50
0.25
Effect
Value
1.5
1.0
0.5
0.0
Effect (only stat. signif.)

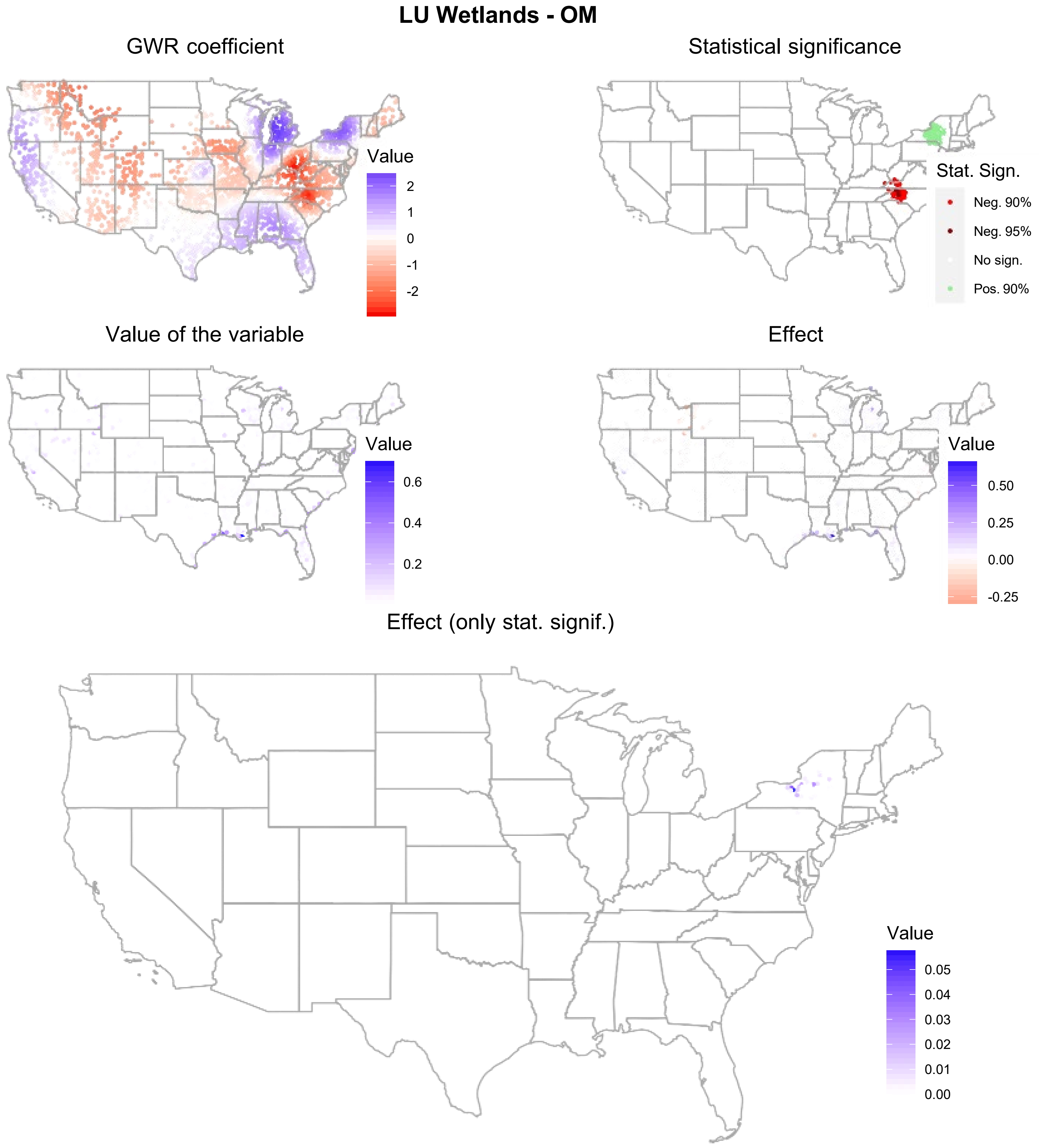
LU Wetlands - OM
GWR coefficient
Value
2
1
0
-1
-2
Statistical significance
Stat. Sign.
Neg. 90%
Neg. 95%
No sign.
Pos. 90%
Value of the variable
Value
0.6
0.4
0.2
Effect
Value
0.50
0.25
0.00
-0.25
Effect (only stat. signif.)
Value
0.05
0.04
0.03
0.02
0.01
0.00

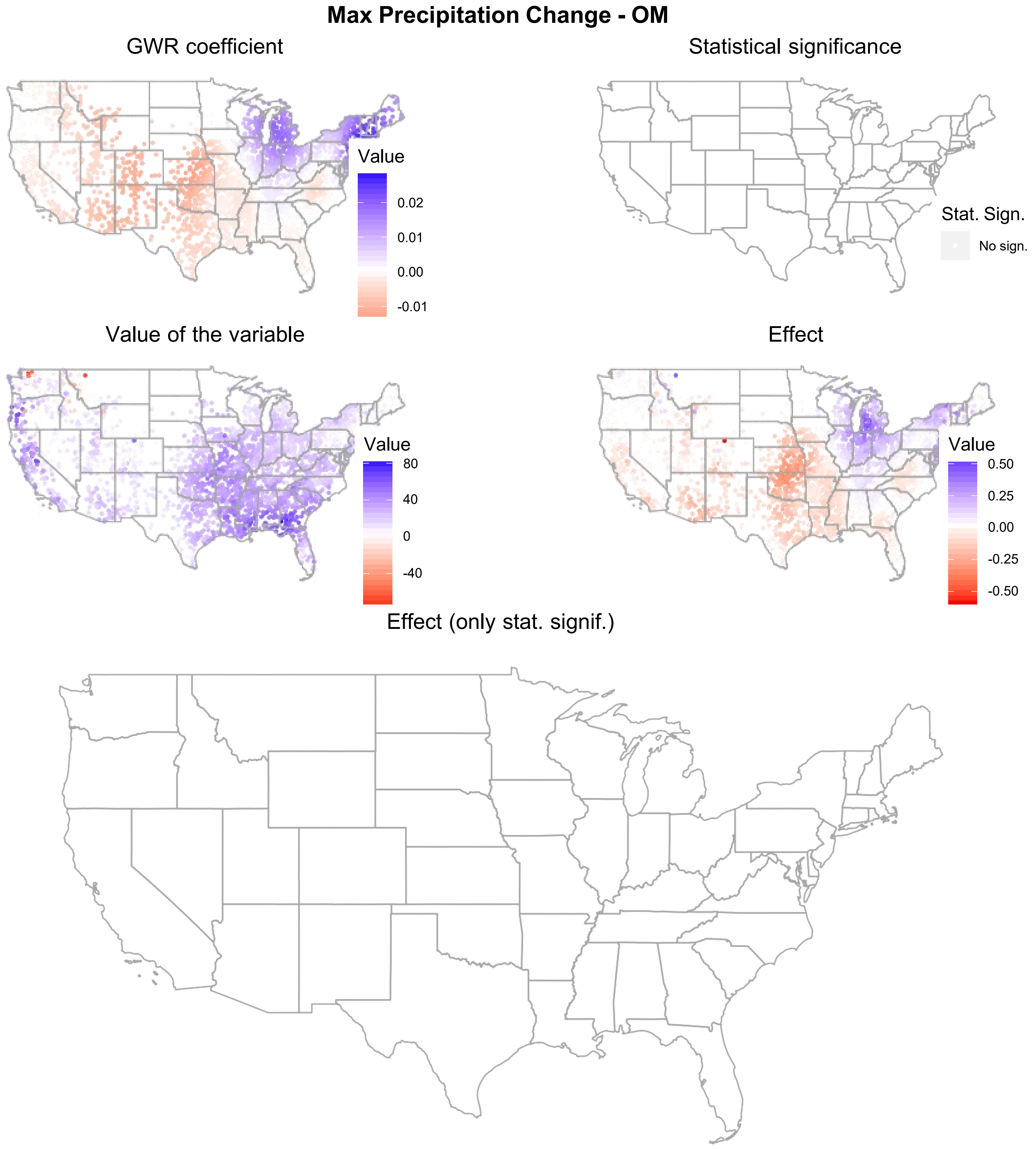
Max Precipitation Change - OM
GWR coefficient
Value
0.02
0.01
0.00
-0.01
Statistical significance
Stat. Sign.
No sign.
Value of the variable
Value
80
40
0
-40
Effect
Value
0.50
0.25
0.00
-0.25
-0.50
Effect (only stat. signif.)

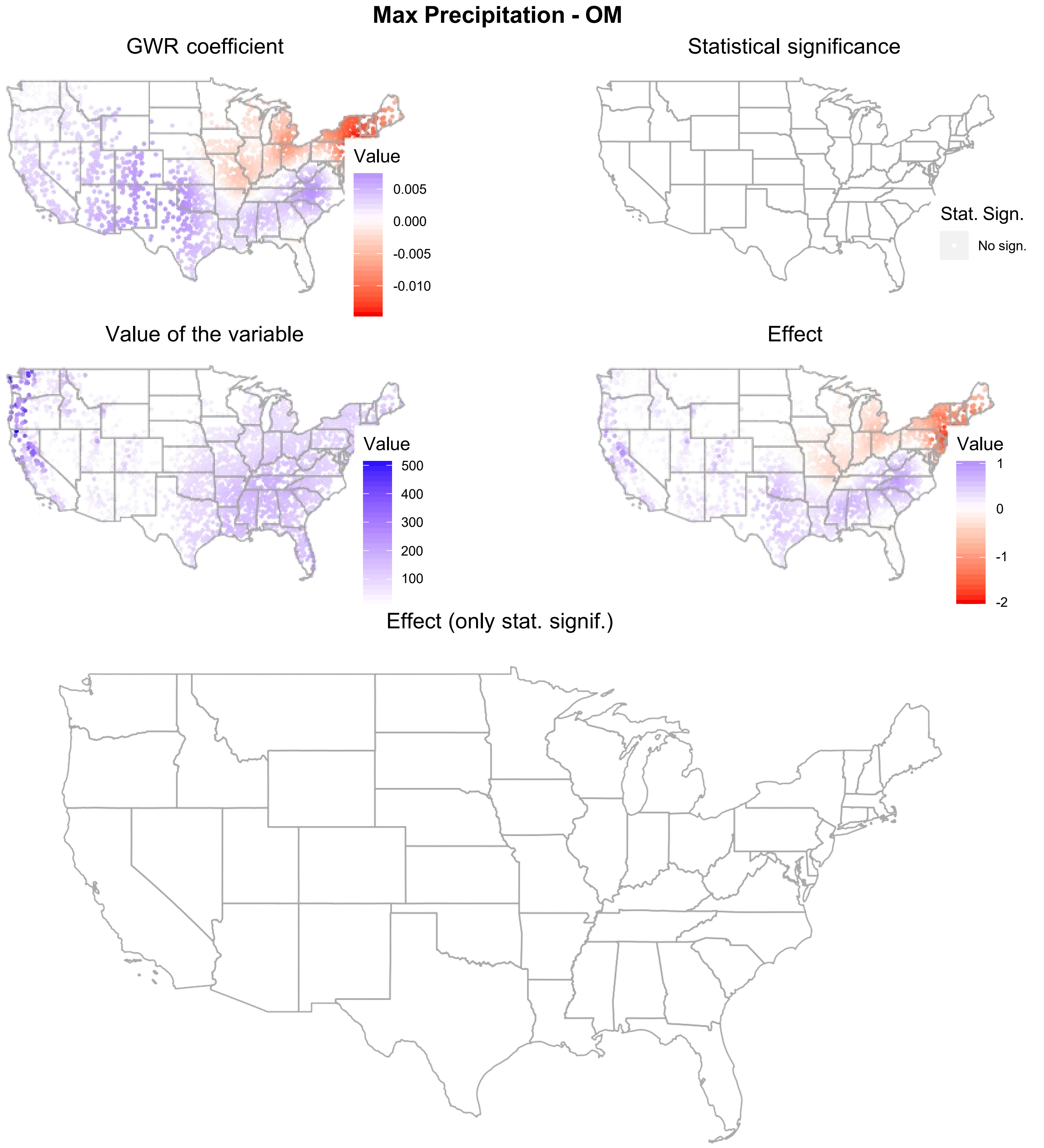
Max Precipitation - OM
GWR coefficient
Value
0.005
0.000
-0.005
-0.010
Statistical significance
Stat. Sign.
No sign.
Value of the variable
Value
500
400
300
200
100
Effect
Value
1
0
-1
-2
Effect (only stat. signif.)

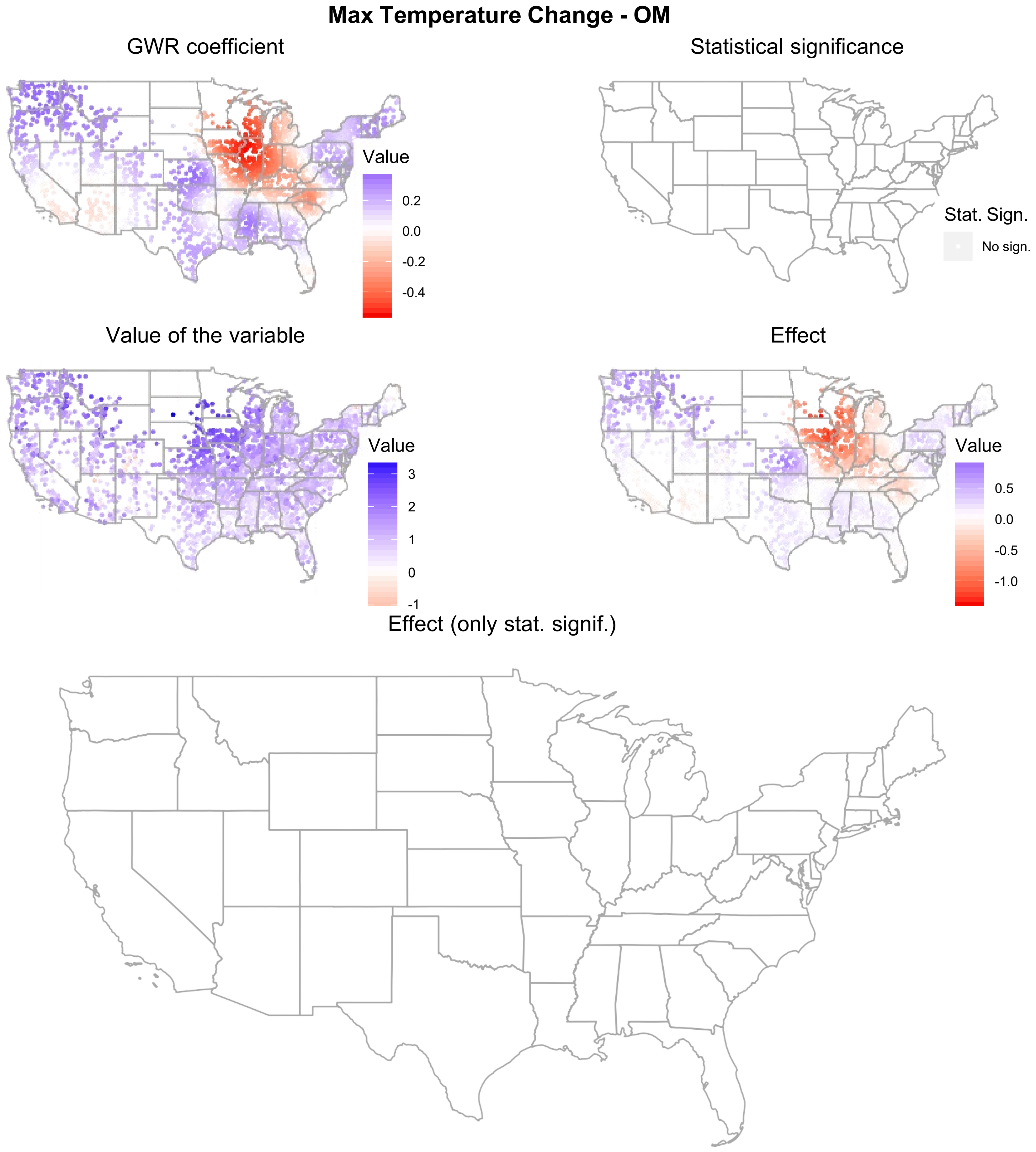

Max Temperature Change - OM
GWR coefficient
Value
0.2
0.0
-0.2
-0.4
Statistical significance
Stat. Sign.
No sign.
Value of the variable
Value
3
2
1
0
-1
Effect
Value
0.5
0.0
-0.5
-1.0
Effect (only stat. signif.)

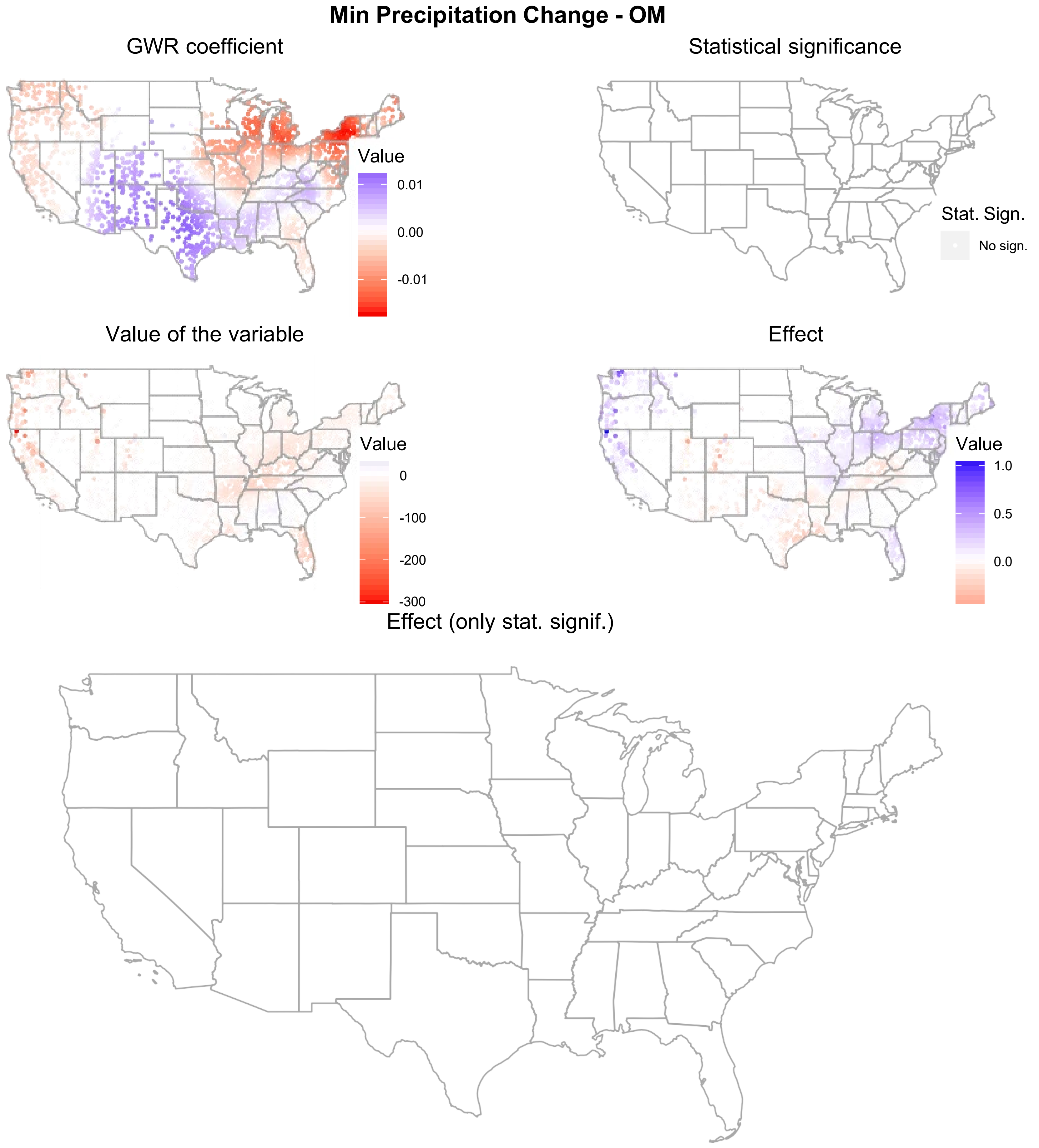
Min Precipitation Change - OM
GWR coefficient
Value
0.01
0.00
-0.01
Statistical significance
Stat. Sign.
No sign.
Value of the variable
Value
0
-100
-200
-300
Effect
Value
1.0
0.5
0.0
Effect (only stat. signif.)

Min Precipitation - OM

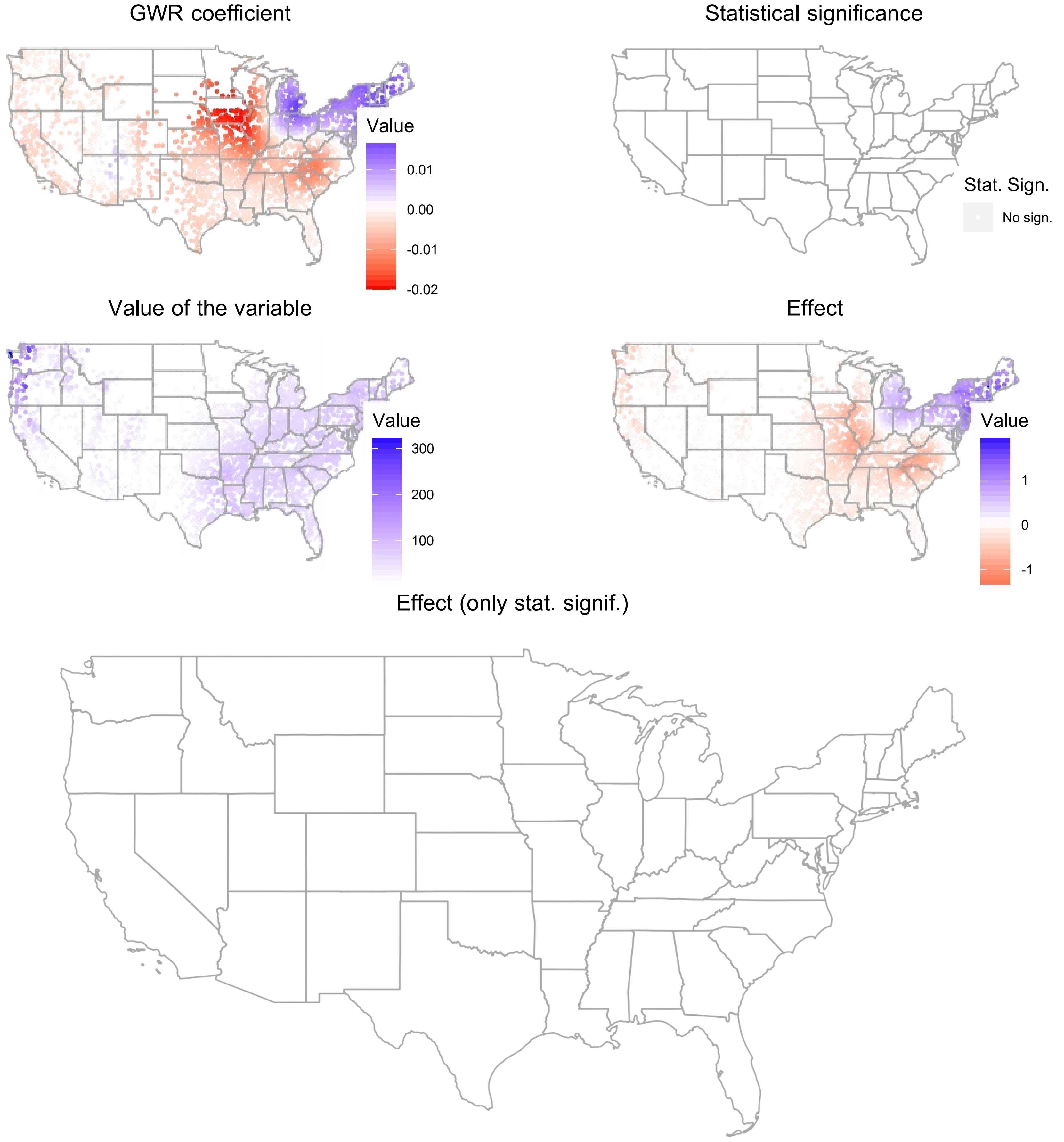

Min Temperature Change - OM

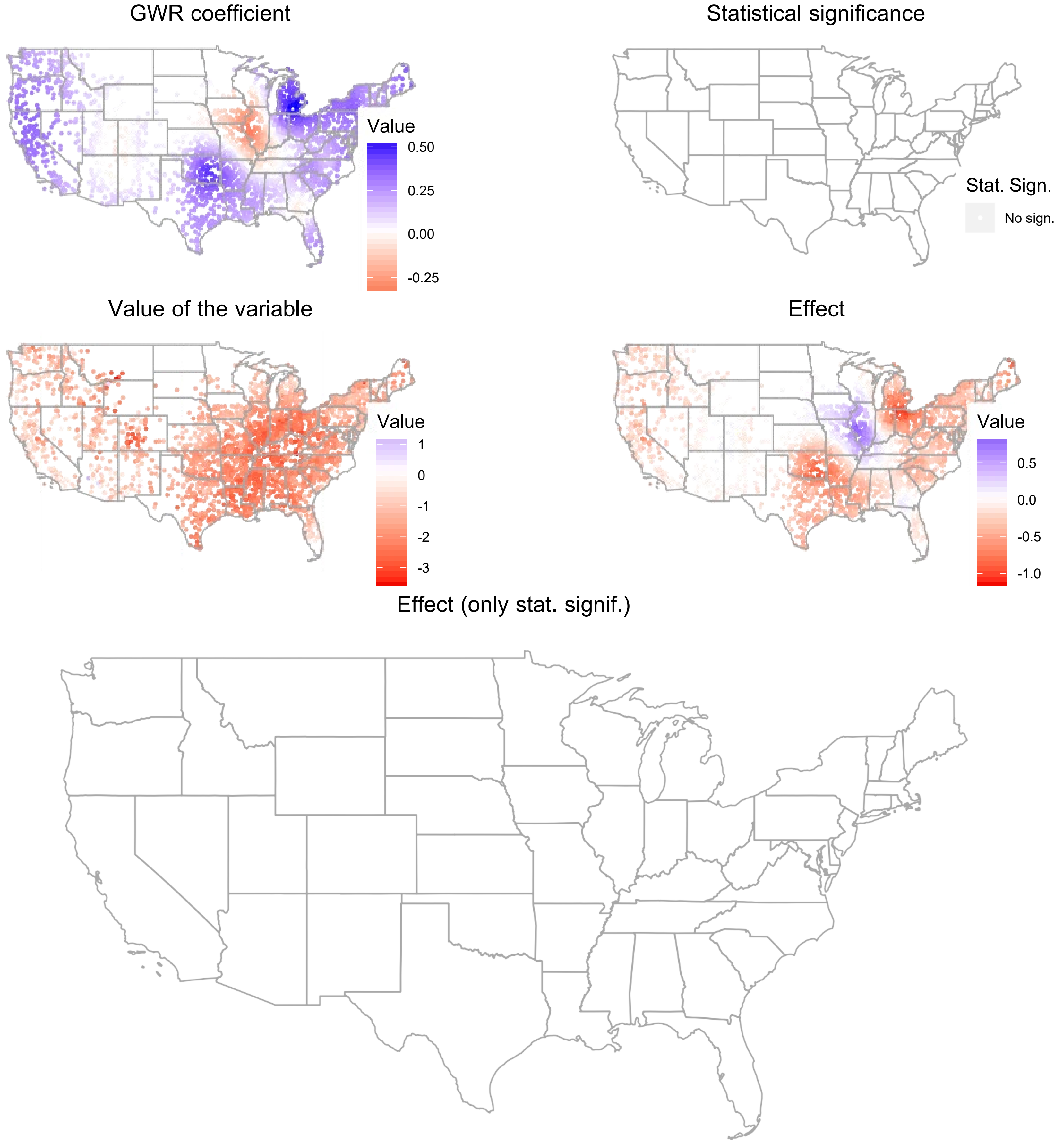

Supplement: Supplementary file 6 — Supplementary Material [file GCB-27-6381-s002.pdf]
